# Cobalt-catalysed C–H carbonylative cyclisation of aliphatic amides†
†Electronic supplementary information (ESI) available. See DOI: 10.1039/c6sc05581h



**DOI:** 10.1039/c6sc05581h

**Published:** 2017-01-10

**Authors:** Patrick Williamson, Alicia Galván, Matthew J. Gaunt

**Affiliations:** a Department of Chemistry , Department of Cambridge , Lensfield Road , Cambridge , CB2 1EW , UK . Email: mjg32@cam.ac.uk

## Abstract

A cobalt-catalysed C–H carbonylation of aliphatic carboxamide derivatives is described, employing commercially available Co(ii)-salts in the presence of a silver oxidant. This operationally simple process utilises an atmospheric pressure of CO and generates a range of substituted succinimide products bearing diverse functional groups that can be successfully accessed *via* this methodology.

## Abstract 2

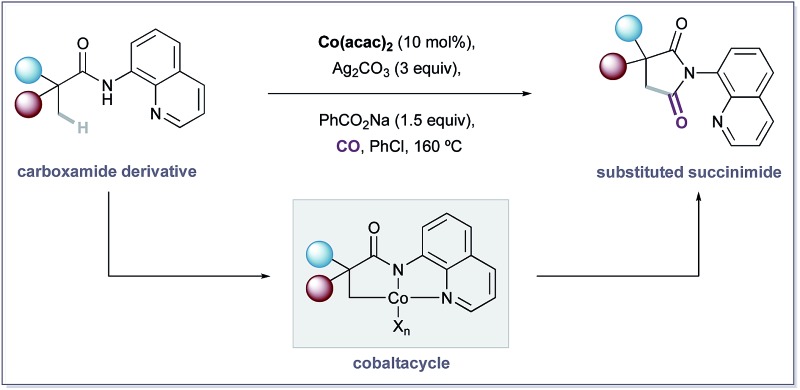
A cobalt-catalysed C–H carbonylation of aliphatic carboxamide derivatives is described, employing commercially available Co(ii)-salts in the presence of a silver oxidant.

The development of novel transition-metal catalysed processes for the selective functionalisation of C–H bonds is an important challenge within synthetic chemistry, enabling efficient and streamlined routes to complex scaffolds.^1^ Whilst noble metals represent the most extensively explored catalyst class for C–H activation, the development of low-cost, 1^st^-row transition-metal alternatives is receiving considerable attention.^2^ Cobalt was the first of these elements to be investigated, with Murahashi *et al.* reporting a successful carbonylative cyclisation procedure of imine and azobenzene substrates as early as 1955 (Scheme 1A).^3^ Since this seminal report, strategies have been developed that directly employ commercially available and bench stable Co(ii) salts for C–H activation reactions.^4^ Monoanionic, bidentate directing groups,^5^ such as the 8-aminoquinoline group first utilized by Daugulis, have been found to be essential for C–H activation to occur *via* these oxidative reaction platforms, and are believed to stabilise postulated high-valent Co-intermediates (Scheme 1A).^6–8^ Whilst this reactivity mode has been widely exploited for the functionalisation of sp^2^-hybridised C–H bonds, analogous C(sp^3^)–H activation procedures remain underdeveloped, with only two known examples reported to date. Ge and co-workers described efficient intra- and intermolecular amidation reactions,^9*a*^ and the Zhang group subsequently developed an alkynylation/cyclisation procedure,^9*b*^ affording substituted pyrrolidinone products in excellent yields (Scheme 1B).^10^ Both processes require the 8-aminoquinoline directing group to promote efficient reaction.

**Scheme 1:**
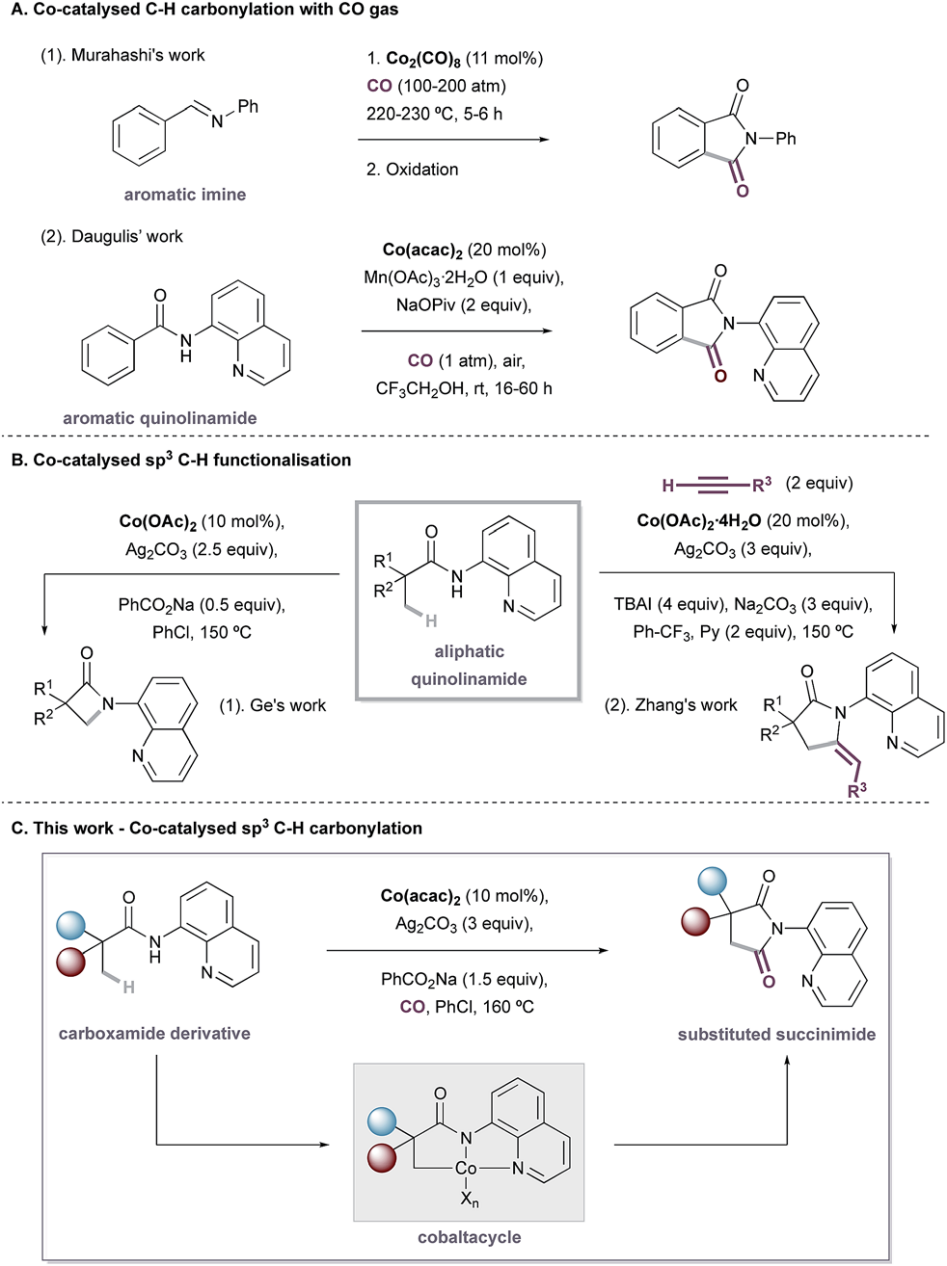
A Co-catalysed C–H carbonylation strategy.

Prompted by our interest in catalytic oxidative C–H carbonylation reactions,^10–12^ we reasoned that its merger with cobalt catalysis, guided by a pyridyl-derived auxiliary, would provide a distinct platform for C(sp^3^)–H activation using earth abundant metals. Herein, we report the development of an oxidative Co-catalysed carbonylative cyclisation procedure of aliphatic quinolinamides to yield a range of substituted succinimide products (Scheme 1C). The reaction proceeds under atmospheric pressures of CO using operationally simple conditions and accommodates a variety of versatile functional groups. During the submission of our manuscript, an elegant study was reported by Sundararaju and co workers, which detailed the same Co-catalyzed carbonylation process.^13^

At the outset of our studies, we prepared a range of pyridyl-derived pivaloylamide substrates (Scheme 2A) and exposed them to reaction conditions based on Ge's C–H amidation process (Co(OAc)_2_, sodium benzoate and Ag_2_CO_3_ in PhCl at 150 °C) under a CO atmosphere.^9^ We observed the formation of the desired C(sp^3^)–H carbonylation product in only one of these cases (Scheme 2B); quinolinamide **1a** was converted to succinimide **7a** with an 86% assay yield. The failure of any of the other substrates to undergo carbonylation highlights an important role of the 8-aminoquinoline group in controlling the C–H activation (Scheme 2B).

**Scheme 2:**
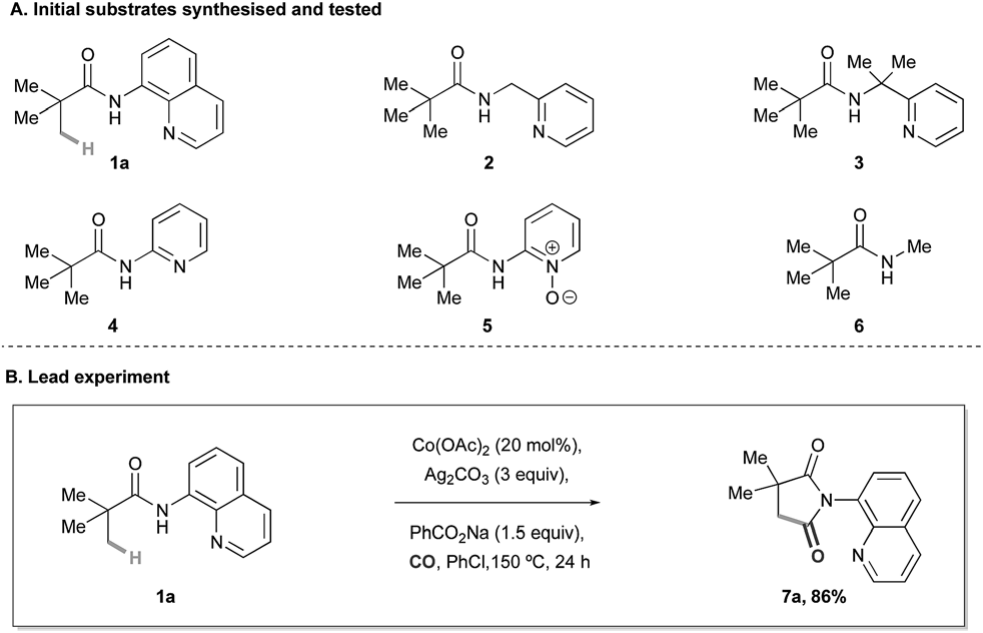
Investigation of substrate structure and lead reaction.

Next, we extensively assessed the reaction parameters of the successful process with the aim of further improving the yield as well as better understanding the role of each of the components. We found that a temperature of at least 150 °C was responsible for an effective reaction, with the yield of the process dramatically reduced upon lowering the temperature by only 15 °C (Table 1, entries 1–4). In the absence of the Co(ii) catalyst, C–H carbonylation did not proceed, confirming the role of the metal species in mediating this process (entry 5). Replacement of sodium benzoate base with sodium acetate, sodium carbonate or sodium phosphate proved detrimental to the efficiency of the process, with succinimide **7a** being formed in diminished 23%, 3% and 2% yield, respectively (entries 6–8). Surprisingly, we found that silver salts were essential for a successful reaction; only traces of the desired product were observed upon replacing Ag(i) oxidants with Mn(OAc)_3_·2H_2_O (entry 9), and complete loss of reactivity was observed when alternative inorganic oxidants were employed (entries 10–11). Although a variety of silver salts were tolerated by the reaction, silver carbonate proved to be the superior oxidant (see ESI†). A range of cobalt(ii) salts were found to be successful pre-catalysts for C–H carbonylation. Whilst a drop in efficiency was observed for CoBr_2_, a modest increase in yield of **7a** to 91% was observed upon employing Co(acac)_2_ (entries 13–14). Lowering the catalyst concentration from 20 mol% to 10 mol% was tolerated by increasing the reaction temperature to 160 °C, allowing succinimide **7a** to be observed in 94% assay yield, which could be isolated in 89% yield (entry 15). Reducing the catalyst loading to 5 mol% did not aid the reaction, and lowering the amount of the silver salt in the reaction was deleterious to the yield (entries 16–18).

**Table 1:** Reaction optimisation^*a*^

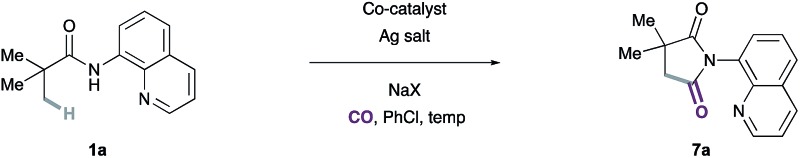					
Entry^*a*^	Co, mol%	Base	Oxidant	*T* °C	Yield^*b*^ (%)
1	Co(OAc)_2_	PhCO_2_Na	Ag_2_CO_3_	150	86
2	Co(OAc)_2_	PhCO_2_Na	Ag_2_CO_3_	140	72
3	Co(OAc)_2_	PhCO_2_Na	Ag_2_CO_3_	135	40
4	Co(OAc)_2_	PhCO_2_Na	Ag_2_CO_3_	130	18
5	No Co	PhCO_2_Na	Ag_2_CO_3_	150	0
6	Co(OAc)_2_	NaOAc	Ag_2_CO_3_	150	23
7	Co(OAc)_2_	Na_2_CO_3_	Ag_2_CO_3_	150	3
8	Co(OAc)_2_	Na_3_PO_4_	Ag_2_CO_3_	150	2
9	Co(OAc)_2_	PhCO_2_Na	Mn(OAc)_3_	150	6
10	Co(OAc)_2_	PhCO_2_Na	NaClO_3_	150	3
11	Co(OAc)_2_	PhCO_2_Na	CAN	150	6
12	Co(OAc)_2_	PhCO_2_Na	No Ag	150	0
13	CoBr_2_	PhCO_2_Na	Ag_2_CO_3_	150	10
14	Co(acac)_2_	PhCO_2_Na	Ag_2_CO_3_	150	91
**15**	**10 mol%**	**PhCO**_**2**_ **Na**	**Ag**_**2**_ **CO**_**3**_	**160**	**94**
16	5 mol%	PhCO_2_Na	Ag_2_CO_3_	160	88
17	5 mol%	PhCO_2_Na	2.0 equiv.	160	78
18	5 mol%	PhCO_2_Na	1.5 equiv.	160	72

^*a*^Starting reaction conditions: substrate **1** (0.2 mmol), Co source (10 mol%), base (1.5 equiv.), oxidant (3.0 equiv.), PhCl (2 mL), CO (1 atm), 21 h.

^*b*^The yield was determined by ^1^H NMR analysis of the crude reaction mixture using 1,1,2,2-tetrachloroethane as the internal standard.

With a set of optimal conditions in hand, we next assessed the scope of the Co-catalyzed C–H carbonylation reaction (Table 2). We found that simple alkyl-derived carboxamides were effective substrates for this reaction affording the corresponding succinimide products in high yields. It is interesting to note that on reaction of **1c** and **1d**, displaying phenyl groups at both the α- and β-position with respect to the carbonyl motif, complete selectivity was observed for C(sp^3^)–H activation at the methyl groups over traditionally more reactive sp^2^-hybridised C–H bonds to form **7c** and **7d**. Although similar selectivities were observed in a related Ni-catalysed C–H carbonylation reaction,^14^ this represents a rare example of competitive aliphatic over aromatic C–H activation. Spirocyclic quinolinamides comprised of a cyclohexane or substituted piperidine moiety also provided synthetically useful yields of the desired succinimide products **7f** and **7g**.

**Table 2:** Reaction scope^*a*^ ^,^ ^*b*^

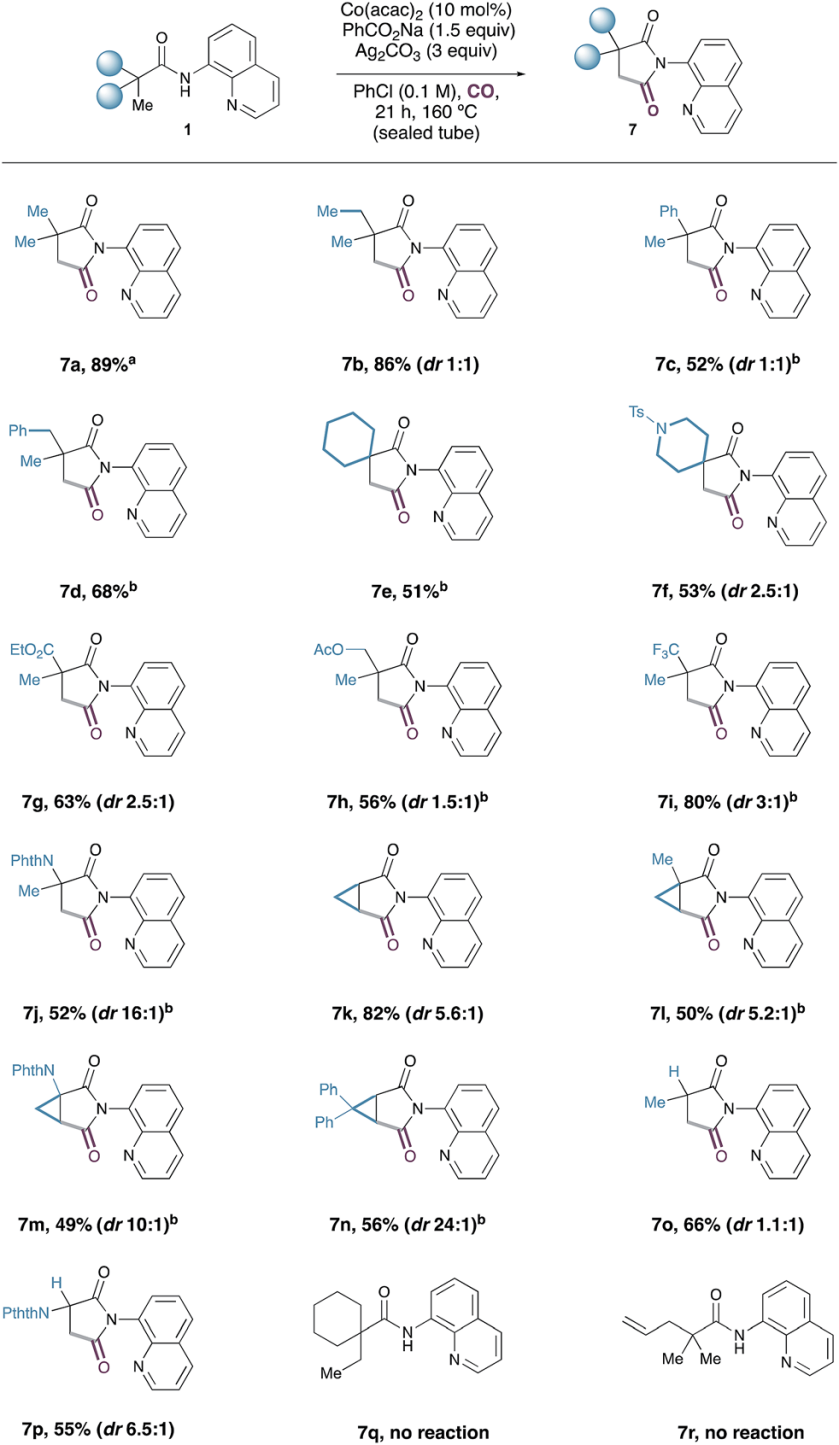

^*a*^80% on 5.00 mmol scale.

^*b*^20 mol% Co(acac)_2_ used.

Substrates containing sensitive ester functionalities, as well as electron-withdrawing trifluoromethyl groups were all accommodated by the reaction, affording the succinimides in 51–80% yield (**7g–7i**). Succinimide **7j** could also be prepared in moderate yield by the C–H carbonylation of a protected α-amino acid derivative. Selective methylene C–H activation onto a cyclopropyl group provided the bicyclic succinimide products in reasonable yields (**7k–7n**). Interestingly, the butyramide-derived substrate,^10^ displaying a partially substituted carbon atom between the carbonyl group and the site of C–H activation successfully underwent C–H carbonylation in 66% yield, to succinimide **7o**; this substrate was unreactive under Ge's Co-catalysed C–H amidation procedure.^9*a*^ Alanine derived **7p**, containing a protected amine group, was also successfully prepared in 55% yield. We found that the Co-catalyzed carbonylation process performed well on a larger, 5 mmol scale to give an 80% yield of the succinimide product **7a**.

Finally, we demonstrated that the succinimide products were compatible with further derivatisation: reductive ring opening of **7c** with LiAlH_4_ generated amino-alcohol **8** in 66% yield; hydrolysis of succinimide **7c** with 3 M aqueous hydrochloric acid led to the formation of substituted succinic acid **9** in 88% yield, with release of the 8-aminoquinoline directing group; and treatment of **7c** with morpholine afforded the ring-opened bis-amide **10** in 77% yield with complete regioselectivity (Scheme 3).

**Scheme 3:**
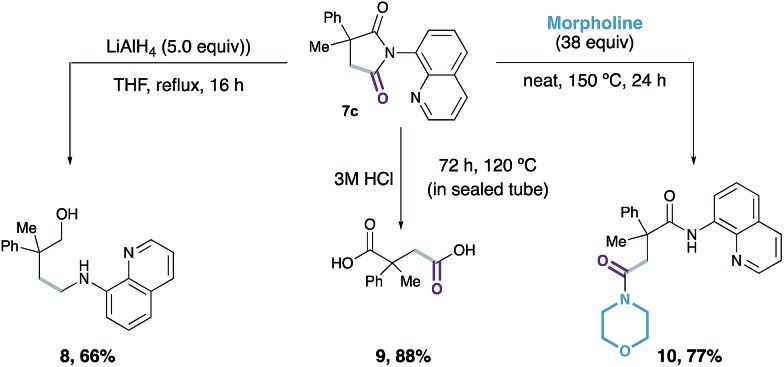
Product derivatisations.

## Conclusions

In summary, we have developed a Co-catalysed carbonylative cyclisation procedure of unactivated, aliphatic C–H bonds. Central to the success of this procedure is the stabilising effect of the quinolinamide directing group. The process tolerates a range of functionalised substrates to generate substituted succinimide products. Importantly, the operationally simple reaction conditions are complemented by the ability utilise an atmospheric pressure of carbon monoxide. While the mechanism of this process remains unclear with respect to the catalytic function of the cobalt salts, an elucidation of the role of the essential stoichiometric Ag additives will also be crucial.^15^ Moreover, to fully realise the full synthetic potential of C–H activation with earth abundant catalysts, an important challenge will be to develop processes that do not rely on precious metal additives and bespoke directing auxiliaries. On going mechanistic studies should reveal further opportunities refine these transformations and lead to the development of new efficient Co-catalyzed C–H activation reactions.
