# Supplementary material for: Cobalt-catalysed C–H carbonylative cyclisation of aliphatic amides
Source: Chem Sci. 2017 Jan 10;8(4):2588–91. doi: 10.1039/c6sc05581h (PMC5431632; doi:10.1039/c6sc05581h)

Electronic Supplementary Material (ESI) for Chemical Science.
This journal is © The Royal Society of Chemistry 2017

Supplementary Materials for Cobalt-catalysed C–H carbonylative cyclisation of aliphatic amides

Table of Contents

	Page
General Information	2
Investigation of Alternative Silver Sources	4
General Procedures for the Synthesis of Starting Materials	4
General Procedure for the Cobalt-Catalysed C-H Activation	18
Product Derivatisations	28
References	29
NMR Spectra	31

General Information

Proton nuclear magnetic resonance (^{1}H NMR) spectra were recorded at ambient temperature on a Bruker AM 400 (400 MHz) or an Avance 500 (500 MHz) spectrometer. Chemical shifts (δ) are reported in ppm and quoted to the nearest 0.01 ppm relative to the residual protons in chloroform-*d* (7.26 ppm), dimethyl sulfoxide- d_6 (2.50 ppm), metanol-d_4 (3.31 ppm) and coupling constants (*J*) are quoted in Hertz (Hz). Data are reported as follows: Chemical shift (multiplicity, coupling constants, number of protons). Coupling constants were quoted to the nearest 0.1 Hz and multiplicity reported according to the following convention: s = singlet, d = doublet, t = triplet, q = quartet, qn = quintet, sext = sextet, sp = septet, m = multiplet, br = broad. Where coincident coupling constants have been observed, the apparent (app) multiplicity of the proton resonance has been reported.

Carbon nuclear magnetic resonance (^{13}C NMR) spectra were recorded at ambient temperature on a Bruker AM 400 (100 MHz) or an Avance 500 (126 MHz) spectrometer. Chemical shift (δ) was measured in ppm and quoted to the nearest 0.1 ppm relative to the residual solvent peaks in chloroform-*d* (77.2 ppm), dimethylsulfoxide-d_6 (39.3 ppm), metanol-d_4 (49.0 ppm). DEPT135, nOe experiments and 2-dimensional experiments (COSY, HMBC and HMQC) were used to support assignments where appropriate.

Fluorine nuclear magnetic resonance (^{19}F NMR) spectra were recorded at ambient temperature on a Bruker AM 400 (396 MHz) spectrometer. Chemical shift (δ) was measured in ppm and quoted to the nearest 0.1 ppm.

High-resolution mass spectra (HRMS) were measured at the EPSRC Mass Spectrometry Service at the University of Swansea. Infrared (IR) spectra were recorded on a Perkin Elmer 1FT-IR Spectrometer fitted with an ATR sampling accessory as either solids or neat films, either through direct application or deposited in chloroform, with absorptions reported in wavenumbers (cm^{-1}).

Analytical thin layer chromatography (TLC) was performed using pre-coated Merck glass backed silica gel plates (Silicagel 60 F254). Flash column chromatography was undertaken on Fluka or Material Harvest silica gel (230–400 mesh) under a positive pressure of

nitrogen unless otherwise stated. Visualization was achieved using ultraviolet light (254 nm) and chemical staining with ceric ammonium molybdate or basic potassium permanganate solutions as appropriate.

Tetrahydrofuran, toluene, hexane, diethyl ether and dichloromethane were dried and distilled using standard methods and chlorobenzene was purchased from Sigma Aldrich. All reagents were purchased at the highest commercial quality and used without further purification. Cobalt (II) acetate and cobalt (II) acetylacetonate were purchased from Sigma Aldrich and silver carbonate was purchased from Alfa Aesar. Reactions were carried out under an atmosphere of nitrogen unless otherwise stated.

Investigation of Alternative Silver Sources

Entry	Silver Source	Yield (%)[a]	Recovered Starting material (%)[a]
1	Ag_2CO_3	94	5
2	AgOAc	55	34
3	Ag_2O	12	50
4	Ag_3PO_4	7	87
5	$AgCO_2CF_3$	10	64
6	$AgIO_3$	23	75
7	AgOTf	4	13
8	$AgNO_3$	25	18
9	$AgCO_2Ph$	41	58
10	$AgClO_4$	24	66

[a] Determined by ^1H NMR using 1,1,2,2-tetrachloroethane as external standard

General Procedures for the Synthesis of Startig Materials

Amide formation from acid chloride

General Procedure A

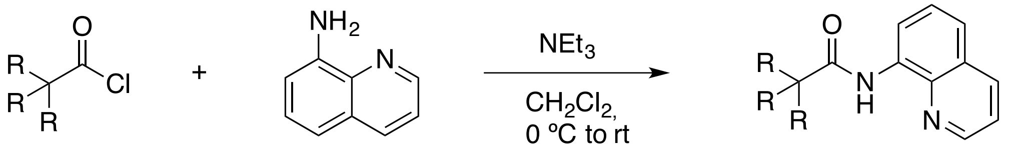

To a solution of acid chloride in CH_2Cl_2 was added a solution of amine and NEt_3 in CH_2Cl_2 at 0 °C. The reaction was allowed to warm to room temperature and stirred. The reaction mixture was diluted with CH_2Cl_2, washed with a saturated aqueous solution of $NaHCO_3$ and water. The organic layer was dried ($MgSO_4$) and concentrated *in vacuo*. The products were purified as described.

***N*-(quinolin-8-yl)pivalamide (1a)**

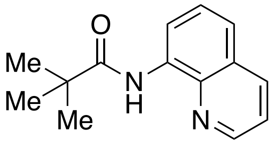

Following General Procedure A, solutions of pivaloyl chloride (2.71 mL, 33 mmol) in CH_2Cl_2 (80 mL) and 8-aminoquinoline (2.88 g, 20 mmol) and NEt_3 (3.07 mL, 22 mmol) in

CH_2Cl_2 (40 mL) yielded product after 30 min, which was purified by silica column chromatography (PE:EtOAc, 10:1) to provide the title compound as a yellow oil (4.46 g, 20 mmol, *quant.*); **^{1}H NMR** (400 MHz, $CDCl_3$) δ: 10.30 (s, 1H), 8.84-8.80 (m, 2H), 8.15 (dd, J = 8.4 Hz, 1.6 Hz, 1H), 7.56-7.48 (m, 2H), 7.44 (dd, J = 8.4 Hz, 4.3 Hz, 1H), 1.45 (s, 9H); **^{13}C NMR** (100 MHz, $CDCl_3$) δ: 177.4, 148.4, 139.0, 136.4, 134.9, 128.1, 127.6, 121.7, 121.4, 116.4, 40.5, 27.9. All data consistent with reported compound.[1]

***N*-(quinolin-8-yl)isobutyramide (1o)**

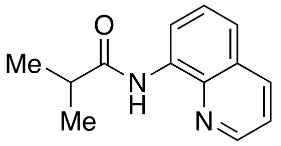

Following General Procedure A, isobutyryl chloride (576 mg, 5.5 mmol) in CH_2Cl_2 (4 mL) and 8-aminoquinoline (720 mg, 5.0 mmol) and NEt_3 (0.77 mL, 5.5 mmol) in CH_2Cl_2 (2 mL) yielded product after 16 h. The crude product was purified by column chromatography (PE:EtOAc, 6:1) to provide the title compound as a colourless oil (960 mg, 4.5 mmol, 90%); **^{1}H NMR** (400 MHz, $CDCl_3$) δ: 9.90 (br. s, 1H), 8.81-8,76 (m, 2H), 8.13 (dd, J= 8.3, 1.7 Hz, 1H), 7.56-7.38 (m, 3H), 2.76 (h, J= 6.9 Hz, 1H), 1.35 (d, J= 6.9 Hz, 6H); **^{13}C NMR** (100 MHz, $CDCl_3$) δ: 175.9, 148.3, 138.6, 136.5, 134.8, 128.1, 127.6, 121.7, 121.4, 116.5, 37.3, 19.9; **HRMS-NSI**: $C_{13}H_{15}N_2O^+$ $[M+H]^+$ requires m/z 215.1179, found 215.1179. All data consistent with reported compound.[2]

Amide formation from carboxylic acid

General Procedure B

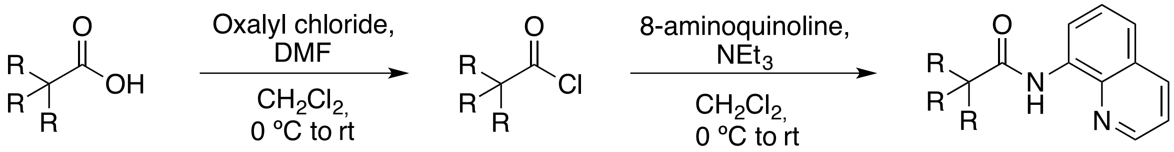

Oxalyl chloride was added drop-wise to a stirred solution of carboxylic acid and 1 drop of DMF in CH_2Cl_2 at 0 °C. The reaction was allowed to warm to room temperature and was stirred for 30 min. The reaction was concentrated *in vacuo*, dissolved in CH_2Cl_2, and the solution was added drop-wise to a solution of amine or ammonium chloride and NEt_3 in CH_2Cl_2 at room temperature. The reaction was stirred for 1 h, concentrated *in vacuo*, diluted with Et_2O, washed with water, brine, dried ($MgSO_4$) and concentrated *in vacuo*. The products were purified as described.

2,2-dimethyl-*N*-(quinolin-8-yl)butanamide (1b)

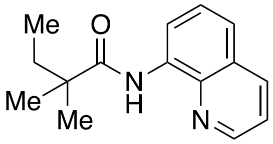

Following General Procedure B, oxalalyl chloride (1.75 mL, 20.0 mmol), 2,2-dimethylbutyric acid (2.10 mL, 16.6 mmol) in CH_2Cl_2 (20 mL) afforded the corresponding acid chloride. Reaction of the acid chloride with 8-amionquinoline (2.87 g, 20.0 mmol) and NEt_3 (7.0 mL, 50.2 mmol) in CH_2Cl_2 (20 mL) yielded product, which was purified by silica column chromatography (PE:EtOAc, 1:0 to 4:1) to provide the title compound as a yellow oil (3.14 g, 12.9 mmol, 78%); **^{1}H NMR** (400 MHz, $CDCl_3$) δ: 10.26 (1H, s), 8.85-8.80 (2H, m), 8.16 (1H, dd, *J* = 8.4 Hz), 7.57-7.48 (2H, m), 7.46 (1H, dd, *J* = 8.4 Hz, 4.3 Hz), 1.79 (2H, q, *J* = 7.5 Hz), 1.41 (6H, s), 0.98 (3H, t, *J* = 7.5 Hz); **^{13}C NMR** (100 MHz, $CDCl_3$) δ: 176.8, 148.4, 139.0, 136.4, 134.9, 128.1, 127.6, 121.7, 121.3, 116.43, 44.2, 34.3, 25.3, 9.5. All data consistent with reported data[1].

2,2-dimethyl-3-phenyl-*N*-(quinolin-8-yl)propanamide (1d)

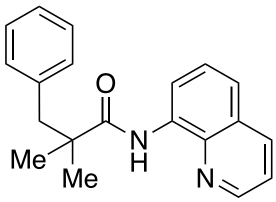

Following General Procedure B, 2,2-dimethyl-3-phenylpropanoic acid (1.98 g, 11.1 mmol) and oxalyl chloride (1.13 mL, 13.3 mmol) in CH_2Cl_2 (12 mL) afforded the corresponding acid chloride. Reaction of the acid chloride with 8-aminoquinoline (1.92 g, 13.3 mmol) and NEt_3 (3.0 mL, 21.8 mmol) in CH_2Cl_2 (20 mL) yielded product, which was purified by silica column chromatography (PE:EtOAc, 4:1) to provide the title compound as a yellow oil (2.12 g, 6.8 mmol, 63%); **^{1}H NMR** (400 MHz, $CDCl_3$) δ: 10.24 (s, 1H), 8.92 (d, *J*= 7.9 Hz, 1H), 8.79-8.76 (m, 1H), 8.13 (d, *J*= 7.9 Hz, 1H), 7.59 (t, *J*= 7.9 Hz, 1H), 7.53-7.89 (m, 1H), 7.45-7.40 (m, 1H), 7.27-7.17 (m, 4H), 3.11 (s, 2H), 1.48 (s, 6H); **^{13}C NMR** (100 MHz, $CDCl_3$) δ: 176.1, 148.2, 138.8, 138.0, 136.2, 134.5, 130.3, 128.0, 127.9, 127.4, 126.4, 121.5, 232.4, 226.3, 46.9, 45.9, 25.3. All data consistent with reported compound.[1]

2,2-diphenyl-*N*-(quinolin-8-yl)cyclopropane-1-carboxamide (1n)

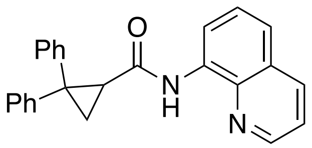

Following General Precedure B, 2,2-diphenylcyclopropane-1-carboxylic acid (298 mg, 1.25 mmol) and oxalyl chloride (0.13 mL, 1.50 mmol) in CH_2Cl_2 (5 mL) afforded the corresponding acid chloride. Reaction of the acid chloride with 8-aminoquinoline (217 mg, 1.50 mmol) and NEt_3 (0.41 mL, 3.00 mmol) in CH_2Cl_2 (5 mL) yielded product, which was purified by silica column chromatography (PE:EtOAc 1:3 – 1:1) to afford the title compound as a white, microcrystalline solid (0.206 g, 0.56 mmol, 45%); **melting point**: 148-150 °C; **IR** ν max/cm^{-1} (neat film): 2361, 2337, 1687, 1523, 1485, 1424, 1336; ^{1}**H NMR** (400 MHz, $CDCl_3$) δ: 10.07 (s, 1H), 8.83 (dd, *J*= 4.4 Hz, 1.7 Hz, 1H), 8.55 (dd, *J*= 6.9 Hz, 2.1 Hz, 1H), 8.15 (dd, 8.2 Hz, 1.7 Hz, 1H), 7.48-7.39 (m, 4H), 7.33-7.14 (m, 9H), 2.71 (dd, *J*= 8.6 Hz, 5.9 Hz, 1H), 2.38 (dd, *J*= 5.9 Hz, 5.0 Hz, 1H), 1.78 (dd, 8.6 Hz, 5.0 Hz, 1H); ^{13}**C NMR** (100 MHz, $CDCl_3$) δ: 167.8, 148.2, 145.4, 139.9, 138.4, 136.6, 134.9, 130.3, 128.7, 128.6, 128.1, 127.6, 127.4, 127.2, 126.6, 121.7, 121.3, 116.5, 40.0, 32.3, 20.6; **HRMS-NSI**: $C_{25}H_{21}ON_2^+$ $[M+H]^+$ requires m/z 365.1648, found 365.1642.

General Procedure C

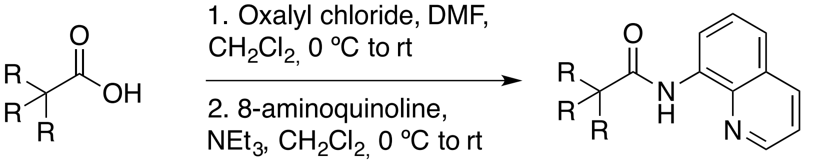

Oxalyl chloride (1.1 equiv) was added drop-wise to a stirred solution of carboxylic acid (1 equiv) and a drop of DMF in CH_2Cl_2 (0.5 M) under argon at 0 °C. The reaction was allowed to warm to room temperature and was stirred for 3 h. The reaction was cooled to 0 ºC and a solution of 8-aminoquinoline (1 equiv) and NEt_3 (3 equiv) in CH_2Cl_2 (0.5 M) was added drop-wise. The reaction was stirred for 16 h at room temperature, quenched with $NaHCO_3$, extract with CH_2Cl_2 (x3), dried ($MgSO_4$) and concentrated *in vacuo*. The products were purified as described.

1-methyl-*N*-(quinolin-8-yl)cyclohexane-1-carboxamide (1e)

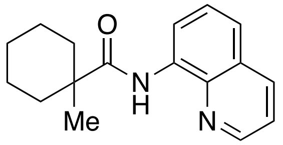

Following General Procedure C, 1-methylcyclohexane-1-carboxylic acid (1.56 g, 11.0 mmol) afforded crude product, which was purified by column chromatography (PE:EtOAc 10:1) to provide the title compound as a yellow oil. (1.13 g, 4.2 mmol, 84%); **^{1}H NMR** (400 MHz, $CDCl_3$) δ: 10.29 (s, 1H), 8.84-8.80 (m, 2H), 8.15 (dd, *J*= 8.3, 1.7 Hz, 1H), 7.55-7.41 (m, 3H), 2.24-2.16 (m, 2H), 1.71-1.38 (m, 8H), 1.36 (s, 3H); **^{13}C NMR** (100 MHz, $CDCl_3$) δ: 176.8, 148.4, 139.0, 136.5, 135.0, 128.1, 127.6, 121.7, 121.3, 116.4, 44.5, 36.0, 26.8, 26.8, 23.2.; **HRMS-NSI**: $C_{17}H_{21}N_2O^+$ $[M+H]^+$ requires m/z 269.1648, found 269.1650. All data consistent with reported compound.[1]

3,3,3-trifluoro-2,2-dimethyl-*N*-(quinolin-8-yl)propanamide (1i)

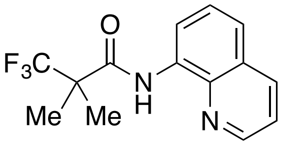

Following General Procedure C, 3,3,3-trifluoro-2,2-dimethylpropanoic acid (1.56 g, 10.0 mmol) afforded crude product, which was purified by column chromatography (PE:EtOAc 6:1) to provide the title compound as a colourless oil. (2.40 g, 8.5 mmol, 85%); **^{1}H NMR** (400 MHz, $CDCl_3$) δ: 10.53 (br. s, 1H), 8.82 (dd, *J*= 4.3, 1.7 Hz, 1H), 8.78-8.73 (m, 1H), 8.14 (dd, *J*= 8.3, 1.7, 1H), 7.55-7,50 (m, 2H), 7.44 (dd, *J*= 8.3, 4.3 Hz, 1H), 1.64 (d, $J_{H\text{-}F}$= 0.7 Hz); **^{13}C NMR** (100 MHz, $CDCl_3$) δ: 167.7, 148.7, 138.8, 136.5, 134.2, 128.0, 127.5 (q, $J_{C\text{-}F}$= 283.0 Hz), 127.4, 122.3, 121.9, 116.8, 49.8 (q, $J_{C\text{-}F}$= 25.3 Hz), 20.19 ($J_{C\text{-}F}$= 2.3 Hz); **^{19}F NMR** (376 MHz, $CDCl_3$) δ: -75.1; **HRMS-NSI**: $C_{14}H_{14}F_3N_2O^+$ $[M+H]^+$ requires m/z 283.1053, found 283.1053. All data cosistent with reported compound.[1]

***N*-(quinolin-8-yl)cyclopropanecarboxamide (1k)**

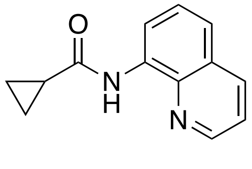

Following General Procedure C, cyclopropanecarboxylic acid (0.64 mL, 8.0 mmol) afforded crude product, which was purified by column chromatography (PE:EtOAc 5:1) to provide the title compound as a white solid (1.25 g, 6 mmol, 74%); **^{1}H NMR** (400 MHz, $CDCl_3$) δ: 10.02 (br. s, 1H), 8.80 (dd, *J*= 4.3, 1.6 Hz, 1H), 8.74 (dd, *J*= 7.4, 1.6 Hz, 1H), 8.13 (dd, *J*= 8.3, 1.6 Hz, 1H), 7.53-7.40 (m, 3H), 1.82-1.76 (m, 1H), 1.18-1.13 (m, 2H), 0.93-0.87 (m, 2H); **^{13}C NMR** (100 MHz, $CDCl_3$) δ: 172.4, 148.2, 138.4, 136.5, 134.9,

128.1, 127.6, 121.7, 121.3, 116.5, 16.3, 8.3; **HRMS-NSI**: $C_{13}H_{13}N_2O^+$ $[M+H]^+$ requires m/z 213.1022, found 213.1023. All data consistent with reported compound.[3]

1-methyl-*N*-(quinolin-8-yl)cyclopropane-1-carboxamide (1l)

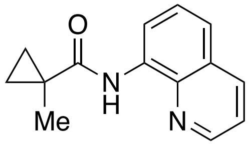

Following General Procedure C, 1-methylcyclopropane-1-carboxylic acid (1.0 g, 10.0 mmol) afforded crude product, which was purified by column chromatography (PE:EtOAc, 5:1) to provide the title compound as a white solid (2.15 g, 9.5 mmol, 95%); **^{1}H NMR** (400 MHz, $CDCl_3$) δ: 10.37 (br. s, 1H), 8.82 (dd, *J*= 4.3, 1.7 Hz), 7.75 (dd, *J*= 7.3, 1.7 Hz, 1H), 8.14 (dd, *J*= 8.3, 1.7 Hz, 1H), 7.55-7.42 (m, 3H), 1.64 (s, 3H), 1.39 (app. q, *J*= 3.8 Hz, 1H), 0.74 (app. q, *J*= 3.8 Hz, 1H); **^{13}C NMR** (100 MHz, $CDCl_3$) δ: 173.9, 148.4, 138.8, 136.4, 134.9, 128.1, 127.6, 121.7, 121.3, 116.3, 20.8, 19.9, 16.9; **HRMS-NSI**: $C_{14}H_{15}N_2O^+$ $[M+H]^+$ requires m/z 227.1179, found 227.1181. All data consistent with reported compound.[4]

General Procedure D

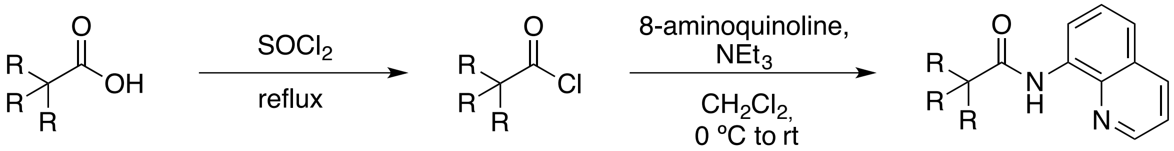

Thionyl chloride (14 equiv) was added to a solution carboxylic acid (1.1 equiv) in CH_2Cl_2 (0.5 M) at room temperature. The reaction was stirred at reflux for 3 h. The reaction was allowed to cool to room temperature, concentrated *in vacuo* and dissolved in CH_2Cl_2 (0.5 M). A solution of 8-aminoquinoline (1 equiv) and NEt_3 (1.1 equiv) in CH_2Cl_2 (0.5 M) was added drop-wise at 0 ºC. The reaction was allowed to warm to room temperature and stirred for 16 h. The mixture was quenched with $NaHCO_3$, extracted with CH_2CL_2 (x3), dried ($MgSO_4$) and concentrated *in vacuo*. The products were purified as described.

2-methyl-2-phenyl-N-(quinolin-8-yl)propanamide (1c)

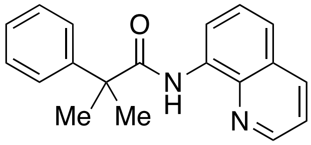

Following General Procedure D, 2-methyl-2-phenylpropanoic acid (1.81 g, 11.0 mmol) afforded crude product, which was purified by column chromatography (PE:EtOAc, 7:1) to provide the title compound as a white solid (2.88 g, 9.9 mmol, 99%); **^{1}H NMR** (400 MHz, $CDCl_3$) δ: 9.87 (s, 1H), 8.78-8.76 (m, 1H), 8.61-8.59 (m, 1H), 8.09-8.06 (m, 1H), 7.58-.48 (m, 3H), 7.46-7.36 (m, 3H), 7.37-7.27 (m, 2H), 1.79 (s, 6H); **^{13}C NMR** (100 MHz, $CDCl_3$) δ: 176.0, 148.3, 145.1, 138.8, 136.3, 134.9, 128.9, 128.0, 127.5, 127.2, 126.5, 121.6, 121.4, 116.2, 48.6, 27.2; **HRMS-NSI**: $C_{19}H_{19}N_2O^+$ $[M+H]^+$ requires m/z 291.1492, found 291.1492. All data cosistent with reported compound.[1]

Procedure for the Synthesis of 4-methyl-*N*-(quinolin-8-yl)-1-tosylpiperidine-4-carboxamide (1f)

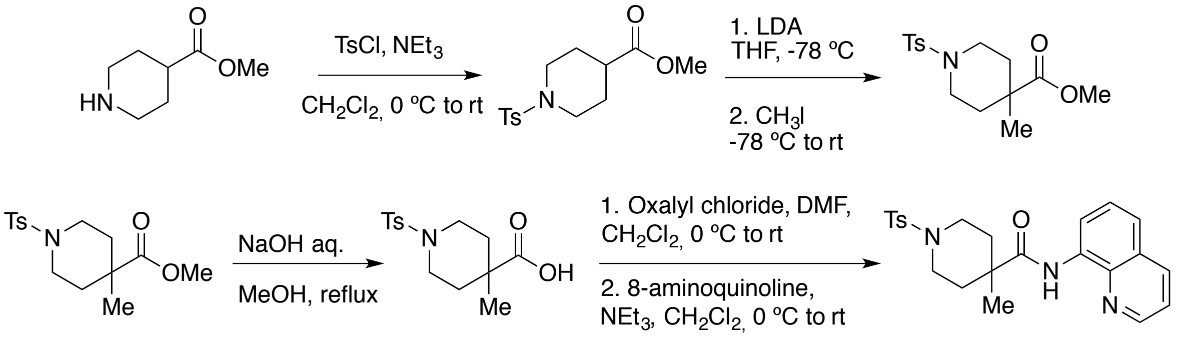

To a stirred solution of methyl piperidine-4-carboxylate (2.86 g, 20 mmol) in CH_2Cl_2 (80 mL) were added NEt_3 (5.58 mL, 40 mmol) and tosyl chloride (4.00 g, 21 mmol) at 0 ºC. The reaction mixture was stirred at room temperature for 3 h. H_2O (80 mL) was added, the organic layer was separated and the aqueous phase was extracted with CH_2Cl_2 (3x 30 mL), dried ($MgSO_4$) and concentrated *in vacuo*. The crude was purified by column chromatography (PE:CH_2Cl_2 3:1 – 0:1), to afford methyl 1-tosylpiperidine-4-carboxylate.

To a solution of DIPA (4.70 mL, 34 mmol) in THF (28 mL) was added a solution of ^{n}BuLi (17.5 mL, 28 mmol, 1.6 M in THF) dropw-wise at 0 °C. The reaction was stirred at 0 °C for 15 min, allowed to warm to room temperature and stirred for 1 h. The resulting solution was added dropw-wise to stirred slurry of methyl 1-tosylpiperidine-4-carboxylate (4.16 g, 14 mmol) in THF (28 mL) −78 ºC. The solution was stirred 1 h at −78 ºC, allowed to warm to room temperature and stirred for 15 min. The solution was cooled to −78 ºC and CH_3I (1.31 mL, 28 mmol) was added dropw-wise. The reaction mixture was stirred at room

temperature for 16 h. A saturated aqueous solution of NH_4Cl was added at 0 ºC and the crude was extracted with CH_2Cl_2 (3 × 30 mL), dried (Na_2SO_4) to afford methyl 4-methyl-1-tosylpiperidine-4-carboxylate that was used in the next step without purification.

To a 4M aqueous solution of NaOH (14 mL, 56 mmol) and MeOH (14 mL) was added 4-methyl-1-tosylpiperidine-4-carboxylate and the reaction was heated at reflux for 24 h. The mixture was cooled to room temperature and the MeOH was removed under *in vacuo*. The aqueous phase was washed with EtOAc, and the pH adjusted to 2-3 with 3M HCl. The aqueous phase was extracted with EtOAc (3 × 20 mL), dried (Na_2SO_4) and concentrated *in vacuo* to afford 4-methyl-1-tosylpiperidine-4-carboxylic acid that was used in the next step without purification. This carboxylic acid was converted to the corresponding amide by the use of General Procedure C and purified by column chromatography (EtOAc:MeOH, 10:1) to provide the title compound as a yellow solid (1.82 g, 4.3 mmol, 43%, 4 steps); **melting point**: 132-134 °C; **IR** ν max/cm^{-1} (neat film): 3351, 2986, 2853,1668, 1528, 1476, 1338, 1321, 1160, 1122, 1091, 1052; **^{1}H NMR** (400 MHz, $CDCl_3$) δ: 10.16 (br. s, 1H), 8.80 (dd (*J*=, 4.3, 1.6 Hz, 1H), 8.67-8.61 m, 1H), 8.16 (dd, *J*= 8.3, 1.6 Hz, 1H), 7.61 (d, *J*= 8.3 Hz, 2H), 7.52-7.43 (m, 3H), 7.24 (d, *J*= 8.3 Hz), 3.52-3.40 (m, 2H), 2.93-2.82 (m, 2H) 2.40-2.32 (m, 2H), 2.36 (s, 3H), 1.85-1.74 (m, 2H), 1.36 (s, 3H); **^{13}C NMR** (100 MHz, $CDCl_3$) δ: 176.8, 148.4, 139.0, 136.5, 135.0, 128.1, 127.6, 121.7, 121.3, 116.4, 44.5, 36.0, 26.8, 26.8, 23.2; **HRMS-NSI**: $C_{23}H_{26}N_3O_3S^+$ $[M+H]^+$ requires m/z 424.1689, found 424.1688.

Procedure for the Synthesis of ethyl 2,2-dimethyl-3-(methyl(quinolin-8-yl)amino)-3-oxopropanoate (1g).

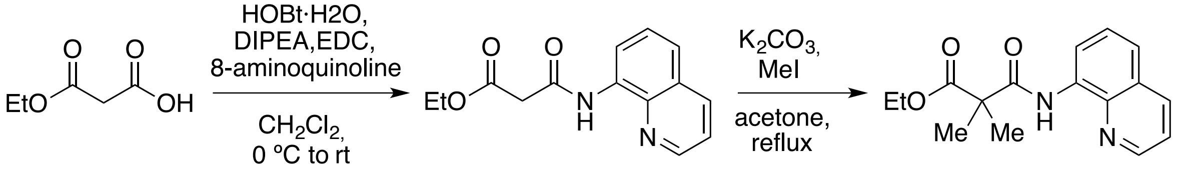

A mixture of 3-ethoxy-3-oxopropanoic acid (1.32 g, 10.0 mmol), 8-aminoquinoline (0.72 mg, 5.0 mmol), $HOBt{\cdot}H_2O$ (0.84 g, 5.5 mmol) and DIPEA (0.96, 5.5 mmol) in anhydrous CH_2Cl_2 (20 mL) was stirred at 0 ºC for 15 min. EDC (1.05g, 5.5 mmol) was added, the mixture was stirred for 15 min at 0 ºC and for 16 h at room temperature. H_2O (50 mL) was added and the mixture was extracted with CH_2Cl_2 (50 mL). The organic layer was washed with water and brine, dried (Na_2SO_4) and concentrated *in vacuo*. The residue was purified by flash column chromatography on silica gel (PE:EtOAc, 2:1), to afford ethyl 3-oxo-3-

(quinolin-8-ylamino)propanoate.

K_2CO_3 (2.57g ,18.56 mmol) was added portion-wise to a solution of ethyl 3-oxo-3-(quinolin-8-ylamino)propanoate (1.20 g, 4.64 mmol) in acetone (10 mL), and the mixture was heated to reflux. Iodomethane (1.16 mL, 18.56 mmol) was added, and the reaction was heated to reflux for 16 h. The solvent was reduced to 1/5 of the original volume *in vacuo*, quenched with ice (10 g) and extracted with CH_2Cl_2 (3 × 20 mL). The combined organic layers were dried (Na_2SO_4) and concentrated *in vacuo*. The residue was purified by column chromatography (PE:EtOAc 4:1), to afford ethyl 2,2-dimethyl-3-(methyl(quinolin-8-yl)amino)-3-oxopropanoate **1g** as a colourless oil (931 mg, 3.3 mmol, 65% two steps); **^{1}H NMR** (400 MHz, $CDCl_3$) δ: 10.61 (s, 1H), 8.83 (dd, *J*= 4.2, 1.5 Hz, 1H), 8.75 (dd, *J*= 6.8, 2.1 Hz, 1H), 8.17 (dd, *J*= 8.3, 1.4 Hz, 1H), 7.57-7.49 (m, 2H), 7.45 (dd, *J*= 8.3, 4.3 Hz, 1H), 4.28 (q, *J*= 7.1 Hz, 2H), 1.66 (s, 3H), 1.31 (t, *J*= 7.1 Hz, 3H); **^{13}C NMR** (100 MHz, $CDCl_3$) δ: 174.1, 170.7, 148.3, 138.7, 136.8, 134.5, 128.1, 127.6, 121.9, 121.7, 117.1; **HRMS-NSI**: $C_{16}H_{19}N_2O_3^+$ $[M+H]^+$ requires m/z 287.1390, found 287.1391. All data consistent with reported compound.[1]

Procedure for the Synthesis of 2,2-dimethyl-3-oxo-3-(quinolin-8-ylamino)propyl acetate (1h)

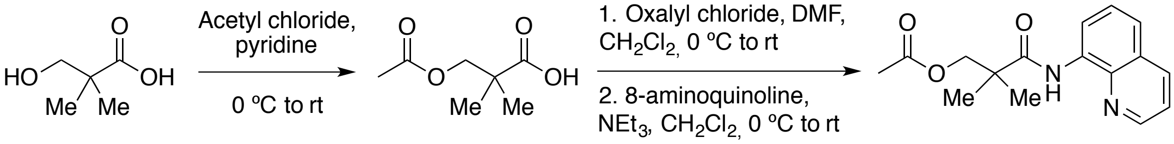

2,2-Dimethyl-3-hydroxypropionic acid (3.5 g, 30 mmol) was dissolved in pyridine (20 mL), and the reaction solution was cooled to 0 °C. Acetyl chloride (0.79 g, 10 mmol) was added dropw-wise, and the reaction was stirred at room temperature for 12 h. 1N HCl (20 mL) was added to adjust the pH of the solution to pH 3, and the reaction mixture was extracted with EtOAc (3 × 10 mL). The organic layer was washed with 1N HCl (4 × 10 mL), dried ($MgSO_4$), concentrated *in vacuo* to afford 2,2-

dimethyl-3-acetyloxypropionic acid. This carboxylic acid was converted to the corresponding amide following General Procedure C. The crude product was purified by column chromatography (PE:EtOAc, 4:1) to provide the title compound as colourless oil. (1.86 g, 6.5 mmol, 65%, two steps); **^{1}H NMR** (400 MHz, $CDCl_3$) δ: 10.45 (br. s, 1H), 8.82-

8.78 (m, 2H), 8.17 (dd, *J*= 8.3, 1.6 Hz, 1H), 7.56-7.44 (m, 3H), 4.26 (s, 2H), 2.15 (s, 3H), 1.44 (s, 6H); **^{13}C NMR** (100 MHz, $CDCl_3$) δ: 174.2, 171.0, 148.3, 139.0, 136.5, 134.8, 128.1, 127.6, 121.8, 121.7, 116.7, 70.5, 43.8, 23.0, 21.1; **HRMS-NSI**: $C_{16}H_{19}N_2O_3^+$ $[M+H]^+$ requires m/z 287.1390, found 287.1392. All data consistent with reported compound.[5]

Procedure for the Synthesis of 2-(1,3-dioxoisoindolin-2-yl)-2-methyl-*N*-(quinolin-8-yl)propanamide (1j) and 2-(1,3-dioxoisoindolin-2-yl)-*N*-(quinolin-8-yl)propanamide (1p)

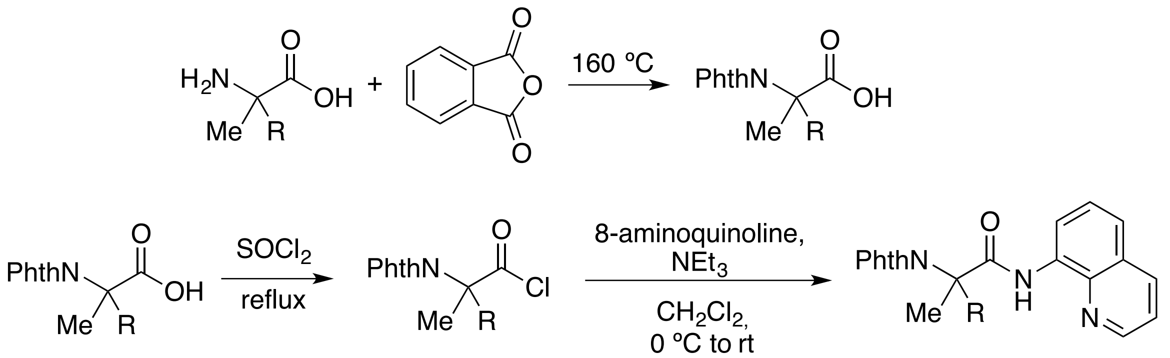

The corresponding carboxylic acid (15 mmol) and phthalic anhydride (2.22g, 15 mmol) were stirred at 160 ºC for 2 h. The reaction was cooled to room temperature, dissolved in EtOAc (30 mL) washed with H_2O (3 × 20 mL), brine (20 mL), dried (Na_2SO_4) and concentrated *in vacuo* to afford the phthalamide protected carboxylic acid. This compound was converted to the corresponding amide following General Procedure D.

2-(1,3-dioxoisoindolin-2-yl)-2-methyl-*N*-(quinolin-8-yl)propanamide (1j)

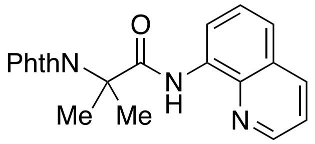

The crude product was purified by column chromatography (PE:EtOAc, 2:1) to provide the title compound as a light brown solid (2.95 g, 8.2 mmol, 55% two steps); **^{1}H NMR** (400 MHz, $CDCl_3$) δ: 10.32 (brs, 1H), 8.75 (dd, *J*= 7.3, 1.4 Hz, 1H), 8.64-8.59 (m, 1H), 8.12-8.06 (m, 1H), 7.80-7.74 (m, 2H), 7.70-7.64 (m, 2H), 7.55-7.44 (m, 2H), 7.39-7.33 (m, 1H), 2.08 (s, 6H); **^{13}C NMR** (100 MHz, $CDCl_3$) δ: 171.2, 168.8, 148.3, 138.8, 136.4, 134.3, 134.2, 132.0, 128.0, 127.5, 123.3, 121.8, 121.7, 116.6, 62.7, 25.1; **HRMS-NSI**: $C_{21}H_{17}N_3O_3^+$ $[M+H]^+$ requires m/z 360.1343, found 360.1344. All data consistent with

reported compound.[6]

2-(1,3-dioxoisoindolin-2-yl)-*N*-(quinolin-8-yl)propanamide (1p)

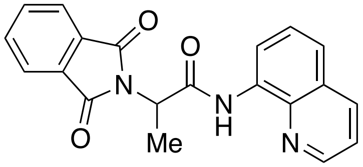

The crude product was purified by column chromatography to provide the title compound as a white solid. (2.33 g, 6.75 mmol, 55%); **^{1}H NMR** (400 MHz, $CDCl_3$) δ: 10.32 (br. s, 1H), 8.72 (dd, *J*=5.4, 3.6 Hz, 1H), 8.68 (dd, *J*= 4.2, 1.6 Hz, 1H), 8.13 (dd, *J*= 8.3, 1.6 Hz, 1H), 7.89 (m, 2H), 7.75 (m, 2H), 7.54-7.48 (m, 2H), 7.41 (dd, *J*= 8.3, 4.3 Hz, 1H), 5.27 (q, *J*= 7.3 Hz), 1.97 (d, *J*= 7.3 Hz, 3H); **^{13}C NMR** (100 MHz, $CDCl_3$) δ: 168.0, 167.4, 148.5, 138.7, 136.5, 124.4, 134.0, 132.2, 128.0, 127.5, 123.7, 122.1, 121.8, 116.8, 50.2, 15.5; **HRMS-NSI**: $C_{20}H_{16}N_3O_3^+$ $[M+H]^+$ requires m/z 346.1188, found 346.1186. All data consistent with reported compound.[7]

Procedure for the Synthesis of Starting Material 1-(1,3-dioxoisoindolin-2-yl)-*N*-(quinolin-8-yl)cyclopropane-1-carboxamide (1m)

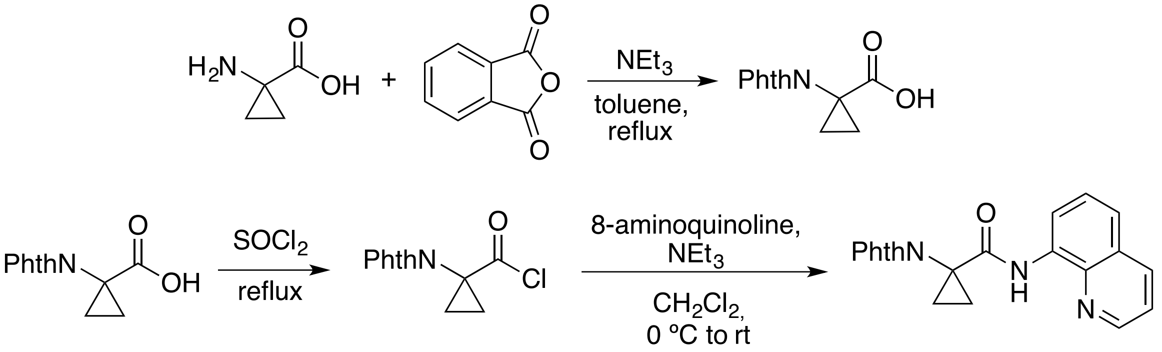

A mixture of 1-aminocyclopropane-1-carboxylic acid (1.01g, 10 mmol), phthalic anhydride (1.48 g, 10 mmol) and NEt_3 (1.39 mL, 10 mmol) in toluene (20 mL) was heated at reflux for 16 h. The reaction was cooled to room temperature, the solvent was removed *in vacuo* and the mixture was disolved in CH_2Cl_2 (20 mL). Concentrated HCl (0.4 mL) was added and the organic phase was washed with H_2O (3 × 20 mL), dried (Na_2SO_4) and concentrated *in vacuo* to afford 1-(1,3-dioxoisoindolin-2-yl)cyclopropane-1-carboxylic acid. This carboxylic acid was converted to the corresponding amide following General Procedure D. The crude product was purified by recrystallisation from hexane to provide the title compound as a yellow solid (2.64 g, 7.4 mmol, 74% over two steps); **melting point**: 232-

234 °C; **IR** ν max/cm^{-1} (neat film): 3318, 1779, 1724, 1675, 1537, 1388, 1330; **^{1}H NMR** (400 MHz, DMSO-d$_6$) δ: 10.3 (br. s, 1H), 8.45 (dd, *J*= 7.6, 1.3 Hz, 1H), 8.40 (dd, *J*= 4.3, 1.7 Hz, 1H), 8.38 (dd, 8.4, 1.7 Hz, 1H), 8.04-7.95 (m, 4H), 7.68 (dd, *J*= 8.4, 1.3 Hz, 1H), 7.59 (t, *J*= 7.9 Hz, 1H), 7.52 (dd, *J*= 8.3, 4.3 Hz, 1H), 1.80 (app. q, *J*= 4.8 Hz, 2H), 1.58 (app. q, *J*= 4.4 Hz, 2H); **^{13}C NMR** (100 MHz, DMSO-d$_6$) δ: 167.9, 149.0, 137.7, 136.8, 135.0, 133.1, 131.3, 127.7, 127.0, 123.4, 122.4, 122.3, 116.1, 33.6, 16.0; **HRMS-NSI**: $C_{21}H_{16}N_3O_3^+$ [M+H]$^+$ requires m/z 358.1186, found 358.1188.

***N*-(pyridin-2-ylmethyl)pivalamide (2)**

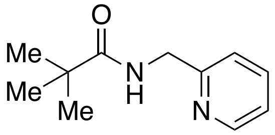

Following General Procedure A, solutions of pivaloyl chloride (1.48 mL, 12 mmol) in CH_2Cl_2 (2 mL) and pyridin-2-ylmethanamine (1.03 mL, 10 mmol) and NEt_3 (1.5 mL, 11 mmol) in CH_2Cl_2 (20 mL) afforded product after 2 h, which was purified by silica column chromatography (PE:EtOAc 1:1 - 0:1) to afford the title compound as a yellow oil (1.96 g, 10 mmol, *quant.*); **^{1}H NMR** (400 MHz, $CDCl_3$) δ: 8.56-8.52 (m, 1H), 7.68-7.63 (m, 1H), 7.28-7.18 (m, 2H), 7.04 (s, 1H), 4.54 (d, *J* = 4.75 Hz, 2H), 1.27 (s, 9H); **^{13}C NMR** (100 MHz, $CDCl_3$) δ: 178.6, 156.8, 149.0, 136.8, 122.3, 122.0, 44.5, 38.8, 27.7. All data in agreement with literature compound.[8]

***N*-(2-(pyridin-2-yl)propan-2-yl)pivalamide (3)**

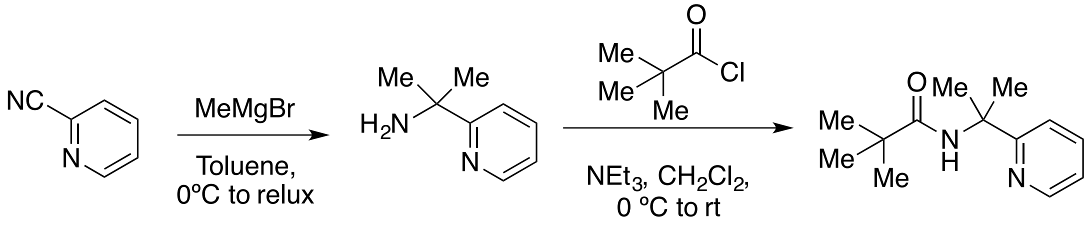

To a stirred solution of picolinonitrile (1.04g, 10 mmol) in toluene (15 mL) was added MeMgBr (3.2 M in 2-methyltetrahydrofuran, 30 mmol) drop-wise at 0 ºC. The reaction was heated to reflux for 16 h, cooled to room temperature and a saturated solution of NH_4Cl (5 mL) was added drop-wise. The mixture was adicified to pH 2 with 3N HCl and washed with EtOAc (3 × 20 mL). The aqueous phase was extracted with CH_2Cl_2 (3 × 20 mL), dried (Na_2SO_4) and concentrated *in vacuo*. The crude was purified by distillation to afford 2-(pyridin-2-yl)propan-2-amine that was converted to the corresponding amide following

General Procedure A. The crude product was purified by column chromatography (PE:EtOAc 2:1) to provide the title compound as a light yellow solid (709 mg, 3.2 mmol, 32% over 2 steps); **^{1}H NMR** (400 MHz, $CDCl_3$) δ: 8.48 (d, *J*= 4.8 Hz, 1H), 8.16 (br. s, 1H), 7.69 (td, *J*= 7.8, 1.7 Hz, 1H), 7.37 (d, *J*= 8.0 Hz, 1H), 7.16 (ddd, *J*= 7.4, 4.8, 1.0 Hz, 1H), 1.72 (s, 6H), 1.23 (s, 9H); **^{13}C NMR** (100 MHz, $CDCl_3$) δ: 177.8, 165.2, 147.7, 137.2, 121.9, 119.6, 56.1, 39.7, 27.9, 27.5; **HRMS-NSI**: $C_{13}H_{21}N_2O^+$ $[M+H]^+$ requires m/z 221.1648, found 221.1648. All data in agreement with literature compound.[9]

***N*-(pyridin-2-yl)pivalamide (4)**

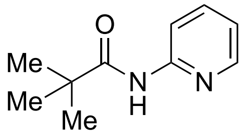

Following General Procedure A, pivaloyl chloride (4.06 mL, 33 mmol), pyridin-2-amine (2.82 g, 33 mmol) and NEt_3 (5.01 mL, 36 mmol) in CH_2Cl_2 (200 mL) afforded product after 16 h, which was purified by column chromatography (PE: EtOAc, 4:1) to provide the title compound as a white solid. (1.69 g, 9.5 mmol, 95%); **^{1}H NMR** (400 MHz, $CDCl_3$) δ: 8.26-8.18 (m, 2H), 8.01 (br. s, 1H), 7.66 (dt, *J*= 7.1, 8.1 Hz, 1H), 6.98-6.97 (m, 1H), 1.30 (s, 9H); **^{13}C NMR** (100 MHz, $CDCl_3$) δ: 177.2, 151.7, 147.8, 138.5, 119.8, 114.1, 39.9, 27.6; **HRMS-NSI**: $C_{10}H_{15}N_2O^+$ $[M+H]^+$ requires m/z 179.1179, found 179.1176. All data in agreement with literature compound.[10]

2-pivalamidopyridine 1-oxide (5)

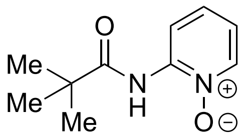

To a stirred solution of *N*-(pyridin-2-yl)pivalamide **4** (1.78g, 10 mmol) in CH_2Cl_2 (50 mL) was added *m*-CPBA (2.42 g, 14 mmol) at 0 ºC. The reaction mixture was stirred for 16 h at room temperature, diluted with CH_2Cl_2 (80 mL), washed with an aqueous saturated solution of of $NaHCO_3$ (20 mL), dried (Na_2SO_4) and concentrated *in vacuo*. The crude product was purified by column chromatography (CH_2Cl_2:acetone 3:1) to provide the title compound as a white solid (1.85 g, 9.5 mmol, 95%); **^{1}H NMR** (400 MHz, $CDCl_3$) δ: 10.30 (br. s, 1H), 8.35 (dd, *J*= 8.5, 1.6 Hz, 1H), 8.17 (dd, *J*= 6.5, 1.6 Hz, 1H), 7.24 (td, *J*= 8.5, 1.6 Hz, 1H), 6.89 (td, *J*= 6.5, 1.6 Hz, 1H), 1.25 (s, 9H); **^{13}C NMR** (100 MHz, $CDCl_3$) δ:

177.5, 144.5, 136.9, 128.3, 118.4, 114.5, 40.5, 27.3; **HRMS-NSI**: $C_{10}H_{14}N_2O_2^+$ $[M+H]^+$ requires m/z 195.1128, found 195.1126. All data in accordance with literature compound.[11]

***N*-methylpivalamide (6)**

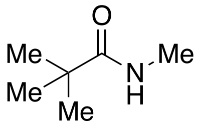

To a solution of pivaloyl chloride (370 μL, 3.0 mmol) in THF (30 mL) was added a solution of methylamine in EtOH (1.1 mL, 33%, 12.0 mmol) drop-wise at 0 °C with stirring. The reaction was allowed to warm to room temperature, and was stirred for 12 h. The mixture was diluted with EtOAc (100 mL), washed with water (3 × 30 mL), brine (30 mL) and dried ($MgSO_4$). The product was purified by silica column chromatography (EtOAc) and recrystallisation from EtOAc to afford the title compound as a white, crystalline solid (315 mg, 2.73 mmol, 91% yield); **melting point:** 87-88 °C; **^{1}H NMR** (400 MHz, $CDCl_3$) δ: 5.65 (br. s., 1H), 2.80 (d, *J*= 4.8 Hz, 3H), 1.19 (s, 9H); **^{13}C NMR** (100 MHz, $CDCl_3$) δ: 179.3, 38.8, 27.8, 26.7. All data in agreement with literature compound.[12]

General Procedure for the Cobalt-Catalysed C-H Activation

General Procedure E. A suspension of quinolinamide (0.20 mmol), $Co(acac)_2$, Ag_2CO_3 (165 mg, 0.60 mmol), $PhCO_2Na$ (43 mg, 0.30 mmol) in PhCl (2 mL) was made up in a microwave vial and purged for 5 min with a balloon of 100% CO. The vial was capped and heated to 160 °C for 21 h. The reaction was allowed to cool to room temperature, diluted with CH_2Cl_2 (10 mL), filtered through celite and concentrated *in vacuo*. The products were purified as described.

3,3-dimethyl-1-(quinolin-8-yl)pyrrolidine-2,5-dione (7a)

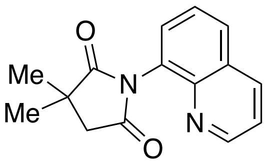

Following General Procedure E, *N*-(quinolin-8-yl)pivalamide **1a** (46 mg, 0.2 mmol) and $Co(acac)_2$ (5 mg, 0.02 mmol) yielded product, which was purified by column chromatography (PE:EtOAc 7:3 to 1:0) to afford the title compound as a white, microcrystalline solid (45 mg, 0.19 mmol, 89% yield); **melting point:** 138-140 °C; **^{1}H NMR** (400 MHz, $CDCl_3$): δ: 8.85 (dd, *J* = 4.3 Hz, 1.7 Hz, 1H), 8.18 (dd, *J* = 8.3 Hz, 1.7 Hz, 1H), 7.93-7.89 (m, 1H), 7.65-7.59 (m, 2H), 7.42 (dd, *J* = 8.4 Hz, 4.2 Hz, 1H), 2.95 (d, *J* = 18.0 Hz, 1H), 2.82 (d, *J* = 18.0 Hz, 1H), 1.61 (s, 3H), 1.50 (s, 3H); **^{13}C NMR** (100 MHz, $CDCl_3$) δ: 183.2 , 175.9, 151.1, 143.8, 136.2, 130.5, 129.8, 129.5, 129.4, 126.2, 122.1, 44.4, 41.2, 26.4, 25.6. All data consistent with reported compound.[1]

3-ethyl-3-methyl-1-(quinolin-8-yl)pyrrolidine-2,5-dione (7b)

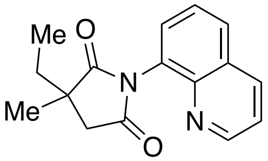

Following General Procedure E, 2,2-dimethyl-*N*-(quinolin-8-yl)butanamide **1b** (49 mg, 0.2 mmol) and $Co(acac)_2$ (7 mg, 0.02 mmol) yielded product, which was purified by column chromatography (PE:EA 1:0 – 3:7) to afford the title compound as a yellow oil and as a mixture of diastereomers in ratio 1:1 (A:B) (46 mg, 0.17 mmol, 65% yield); **^{1}H NMR** (400 MHz, $CDCl_3$) δ: 8.85 (ddd, *J*=8.9 Hz, 4.2 Hz, 1.8 Hz, 1H, diast. A and B), 8.18 (dt, *J*= 8.4 Hz, 2.1 Hz, 1H, diast. A and B), 7.95-7.89 (m, 1H, diast. A and B), 7.65-7.57 (m, 2H, diast.

A and B) 7.45-7.40 (m, 1H, diast A and B), 3.03 (d, *J* = 18.2 Hz, 1H, diast. A), 2.89 (d, *J* = 18.3 Hz, 1H, diast. B), 2.82 (d, J = 18.3 Hz, 1H, diast. B), 2.67 (d, *J* = 18.2 Hz, 1H, diast. A), 2.06-1.71 (m, 2H, diast. A and B), 1.58 (s, 3H, diast. B), 1.47 (s, 3H, diast. A), 1.17 (t, *J* = 7.5 Hz, 3H, diast. A), 1.06 (t, *J* = 7.4 Hz, 3H, diast B); **^{13}C NMR** (100 MHz, $CDCl_3$) 182.8 (diast. A), 182.7 (diast. B), 176.2 (diast. A), 176.2 (diast. B), 151.2 (diast. A), 151.0 (diast. B), 143.8 (diast. A), 143.8 (diast. B), 136.2 (diast. A), 136.2 (diast. B), 130.7 (diast. A), 130.5 (diast. B), 129.8 (diast. A), 129.7 (diast. B), 129.6 (diast. A), 129.5 (diast. B), 129.4 (diast. A), 129.4 (diast. B), 126.2 (diast. A), 126.2 (diast. B), 122.1 (diast. A), 122.1 (diast. B), 45.2 (diast. A), 45.2 (diast. B), 31.6 (diast. A), 31.3 (diast. B), 24.7 (diast. A), 24.2 (diast. B), 9.1 (diast. A), 9.1 (diast. B). All data consistent with reported compound.[1]

3-methyl-3-phenyl-1-(quinolin-8-yl)pyrrolidine-2,5-dione (7c)

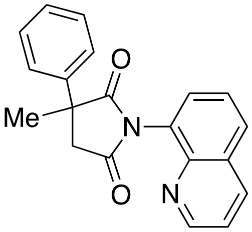

Following general procedure E, 2-methyl-2-phenyl-*N*-(quinolin-8-yl)propanamide **1c** (58 mg, 0.20 mmol) and $Co(acac)_2$ (10 mg, 0.04 mmol) yielded product, which was purified by column chromatography (EtOAc) to afford the title compound as a white solid and as a mixture of diastereoisomers in ratio 1:1 (A:B). (33 mg, 0.10 mmol, 52%); **^{1}H NMR** (400 MHz, $CDCl_3$) δ: 8.93-8.87 (m, 1H, diast. A and B), 8.20 (d, *J*= 8.3 Hz, 1H, diast. A and B), 7.97-7.90 (m, 1H, diast. A and B), 7.78 (d, *J*=7.6, 1H, diast. A and B), 7.71-7.60 (m, 2H, diast. A and B), 7.56 (d, *J*=7.6, 1H, diast. A and B), 7.48-7.40 (m, 3H, diast. A and B), 7.37-7.30 (m, 1H, diast. A and B), 3.40 (d, *J*= 18.3 Hz, diast. A), 3.38 (d, *J*= 18.0 Hz, 1H, diast. B), 3.28 (d, *J*= 18.0 Hz, 1H, diast. B), 3.13 (d, *J*=18.3 Hz, diast. A), 2.02 (s, 3H, diast. A), 1.93 (s, 3H, diast. B); **^{13}C NMR** (100 MHz, $CDCl_3$) diast. A and B δ: 181.1, 180.8, 175.6, 151.2, 151.1, 143.8, 142.7, 142.3, 136.3, 130.6, 130.5, 130.0, 129.9, 129.7, 129.6, 129.4, 129.2, 129.0, 128.8, 127.7, 127.6, 127.5, 126.6, 126.3, 126.0, 122.2, 49.0, 48.8, 46.8, 45.8, 26.6, 25.2; **HRMS-NSI**: $C_{20}H_{17}N_2O_2^+$ $[M+H]^+$ requires m/z 317.1285, found 317.1288. All data consistent with reported compound.[1]

3-benzyl-3-methyl-1-(quinolin-8-yl)pyrrolidine-2,5-dione (7d)

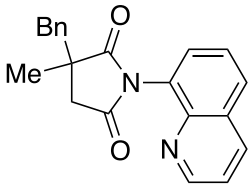

Following General Procedure E, 2,2-dimethyl-3-phenyl-*N*-(quinolin-8-yl)propanamide **1d** (61 mg, 0.20 mmol) and $Co(acac)_2$ (5 mg, 0.02 mmol) yielded product, which was purified by column chromatography (PE:EtOAc 4:1 – 0:1) to afford the title compound as a yellow oil and as a mixture of diastereomers in ratio 1:0.4 (A:B) (35 mg, 0.11 mmol, 53%); **^{1}H NMR** (400 MHz, $CDCl_3$) δ: 8.85 (dd, *J*= 4.3 Hz, 1.6 Hz, 1H, diast. A), 8.79 (dd, *J*= 4.2 Hz, 1.2 Hz, 1H, diast. B), 8.20-8.14 (m, 1H, diast. A and B), 7.94-7.91 (m, 1H, diast. B), 7.88 (dd, *J*= 8.3 Hz, 1.3 Hz, 1H, diast. A), 7.65-7.60 (m, 2H, diast. B), 7.56-7.53 (m, 1H, diast. A), 7.44-7.26 (m, 6H, diast. A and B), 7.16 (dd, *J*= 7.3 Hz, 1.3 Hz, diast. A); **^{13}C NMR** (100 MHz, $CDCl_3$) δ: 182.5 (diast. B), 182.0 (diast. A), 175.8 (diast. B), 175.6 (diast. A), 151.2 (diast. A), 151.0 (diast. B), 143.8 (diast. A), 143.8 (diast. B), 136.6 (diast. A), 136.6 (diast. B), 136.2 (diast. A), 136.1 (diast. B), 130.8 (diast. A), 130.4 (diast. B), 130.3 (diast. B), 129.8 (diast. B), 129.7 (diast. A), 129.5 (diast. A), 129.5 (diast. B), 129.3 (diast. B), 128.9 (diast. A), 128.6 (diast. A), 127.6 (diast. A), 127.1 (diast. B), 126.2 (diast. A), 126.2 (diast. B), 122.1 (diast. A), 122.1 (diast. B), 46.3 (diast. A), 46.0 (diast. B), 44.2 (diast. A), 43.4 (diast. B), 40.8 (diast. B), 40.4 (diast. A), 25.9 (diast. A), 24.9 (diast. B). All data consistent with reported compound.[1]

2-(quinolin-8-yl)-2-azaspiro[4.5]decane-1,3-dione (7e)

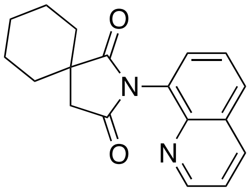

Following General Procedure E, 1-methyl-*N*-(quinolin-8-yl)cyclohexane-1-carboxamide **1e** (54 mg, 0.20 mmol) and $Co(acac)_2$ (10 mg, 0.04 mmol) yielded product, which was purified by column chromatography (EtOAc containing 1% of NEt_3) to provide the title compound as a white solid (40 mg, 0.14 mmol, 68%); **^{1}H NMR** (400 MHz, $CDCl_3$) δ: 8.84 (dd, *J*= 4.2, 1.6 Hz, 1H), 8.17 (dd, *J*= 8.3, 1.5 Hz, 1H), 7.92-7.87 (m, 1H), 7.64-7.58 (m, 2H), 7.40 (dd, *J*= 8.3, 4.2 Hz, 1H), 2.97 (d, *J*= 18.1 Hz, 1H), 2.80 (d, *J*= 18.1 Hz, 1H),

2.05-1.94 (m, 3H), 1.90-1.82 (m, 2H), 1.79-1.68 (m, 2H), 1.52-1.34 (m, 3H); **^{13}C NMR** (100 MHz, $CDCl_3$) δ: 182.8, 176.2, 151.1, 143.7, 136.2, 130.4, 129.7, 129.6, 129.4, 122.0, 45.9, 40.8, 33.9, 33.7, 25.2, 22.3; **HRMS-NSI**: $C_{18}H_{19}N_2O_2^+$[M+H]$^+$ requires m/z 295.1441, found 295.1443. All data consistent with reported compound.[1]

2-(quinolin-8-yl)-8-tosyl-2,8-diazaspiro[4.5]decane-1,3-dione (7f)

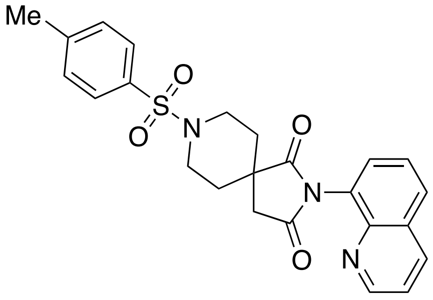

Following General Procedure E, 4-methyl-*N*-(quinolin-8-yl)-1-tosylpiperidine-4-carboxamide **1f** (85 mg, 0.20 mmol) and $Co(acac)_2$ (10 mg, 0.04 mmol) yielded product, which was purified by column chromatography (EtOAc:MeOH 10:1) to provide the title compound as an colourless oil (62 mg, 0.14 mmol, 68%); **IR** ν max/cm^{-1} (neat film): 3052, 3022, 3962, 1708, 1596, 1500, 1473, 1398, 1342, 1324; **^{1}H NMR** (400 MHz, $CDCl_3$) δ: 8.80 (dd, *J*= 4.2, 1.6 Hz, 1H), 7.91 (dd, 7.7, 1.9 Hz, 1H), 7.66 (d, *J*= 8.3 Hz, 2H), 7.63-7.53 (m, 2H), 7.42 (dd, *J*= 8.3, 4.2 Hz, 1H), 7.31 (d, *J*= 8.3 Hz, 2H), 3.62-3.50 (m, 2H), 3.06-2.93 (m, 2H), 2.83 (d, *J*= 18.0 Hz, 1H), 2.69 (d, *J*= 18.0 Hz, 1H), 2.46-2.35 (m, 1H), 2.41 (s, 3H), 2.33-2.22 (m, 1H), 2.13-2.02 (m, 1H), 1.95-1.84 (m, 1H); **^{13}C NMR** (100 MHz, $CDCl_3$) δ: 180.9, 174.7, 151.2, 143.9, 143.5, 136.3, 133.3, 130.0, 129.9, 129.5, 129.4, 127.8, 126.2, 122.2, 42.8, 42.7, 42.5, 41.3, 33.9, 33.1, 21.7; **HRMS-NSI**: $C_{24}H_{24}N_3O_4S^+$ [M+H]$^+$ requires m/z 450.1482, found 450.1479.

Ethyl 3-methyl-2,5-dioxo-1-(quinolin-8-yl)pyrrolidine-3-carboxylate (7g)

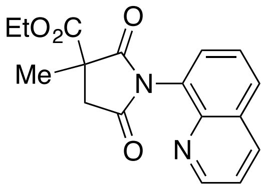

Following General Procedure E, ethyl 2,2-dimethyl-3-oxo-3-(quinolin-8-ylamino)propanoate **1g** (57 mg, 0.20 mmol) and $Co(acac)_2$ (5mg, 0.02 mmol) yielded product, which was purified by column chromatography to provide the title compound as a colourless oil and as a mixture of diastereoisomers in ratio 2.5:1 (A:B) (39 mg, 0.13 mmol,

63%); **^{1}H NMR** (400 MHz, $CDCl_3$) δ: 8.89-8.81 (m, 1H, diast. A and B), 8.23-8.14 (m, 1H, diast. A and B), 7.96-7.89 (m, 1H, diast. A and B), 7.68-7.58 (m, 1H, diast. A and B), 7.47-7.39 (m, 1H, diast. A and B), 4.36-4.25 (m, 1H, diast. A and B), 3.66 (d, *J*= 18.0 Hz, 1H, diast. B), 3.42 (d, *J*= 18.0 Hz, 1H, diast. A), 2.98 (d, *J*= 18.0 Hz, diast. A), 2.80 (d, *J*= 18.0 Hz, 1H, diast. B), 1.85 (s, 3H, diast A.), 1.76 (s, 3H, diast. B), 1.35 (t, *J*= 7.1, diast. B), 1.34 (t, *J*= 7.2 Hz, diast. A); **^{13}C NMR** (100 MHz, $CDCl_3$) diast A δ: 176.3, 174.8, 170.5, 151.2, 143.7, 136.4, 130.1, 129.6, 129.4, 126.3, 122.2, 62.6, 51.7, 41.7, 21.0, 14.2; **^{13}C NMR** (100 MHz, $CDCl_3$) diast B δ: 176.0, 174.6, 170.0, 151.0, 143.5, 136.1, 130.0, 129.6, 129.4, 126.1, 122.2, 62.7, 51.6, 41.4, 21.5, 14.2; **HRMS-NSI**: $C_{17}H_{17}N_2O_4^+$ $[M+H]^+$ requires m/z 313.1183, found 313.1183. All data consistent with reported compound.[1]

(3-methyl-2,5-dioxo-1-(quinolin-8-yl)pyrrolidin-3-yl)methyl acetate (7h)

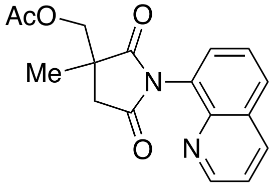

Following General Procedure E, 2,2-dimethyl-3-oxo-3-(quinolin-8-ylamino)propyl acetate **1h** (57 mg, 0.20 mmol) and $Co(acac)_2$ (10 mg, 0.04 mmol) yielded product, which was purified by column chromatography (EtOAc) to provide the title compound as a white solid and as a mixture of diastereoisomers in ratio 1.5:1 (A:B) (35 mg, 0.11 mmol, 56%); **melting point**: 130-132 °C; **IR** ν max/cm^{-1} (neat film): 3010, 2925, 2852, 1744, 1710, 1402, 1231, 1209, 1044, 794; **^{1}H NMR** (400 MHz, $CDCl_3$) δ: 8.87 (dd, *J*= 4.2, 1.7 Hz, 1H, diast. A), 8.84 (dd, *J*= 4.2, 1.7 Hz, 1H, diast. B), 8.20 (td, *J*= 8.4, 1.7, 1H, diast. A and B), 7.95-7.91 (m, 1H, diast. A and B), 7.66-7.59 (m, 2H, diast. A and B), 7.45 (dd, *J*= 8.4, 4.2 Hz, 1H, diast. B), 7.43 (dd, *J*= 8.4, 4.2 Hz, 1H, diast. A), 4.52 (d, *J*= 10.8 Hz, 1H, diast. B), 4.37 (d, *J*= 10.9 Hz, 1H, diast. A), 4.26 (d, *J*= 10.9 Hz, 1H, diast. A), 4.25 (d, *J*= 10.8 Hz, 1H, diast. B), 3.23 (d, *J*= 18.2 Hz, 1H, diast. B), 3.04 (d, *J*= 18.0 Hz, 1H, diast. A), 2.90 (d, *J*= 18.0 Hz, 1H, diast. A), 2.74 (d, *J*= 18.2 Hz, 1H, diast. B), 2.18 (s, 3H, diast. B), 2.13 (s, 3H, diast. A), 1.61 (s, 3H, diast. A), 1.51 (s, 3H, diast. B); **^{13}C NMR** (100 MHz, $CDCl_3$) diast A δ: 180.1, 175.3, 170.6, 151.0, 143.7, 136.3, 130.0, 129.5, 129.4, 126.2, 122.2, 68.0, 44.8, 39.6, 21.3, 20.9; **^{13}C NMR** (100 MHz, $CDCl_3$) diast B δ: 180.0, 175.2, 170.8, 151.1, 143.5, 136.5, 130.2, 130.1, 129.6, 129.4, 126.3, 122.2, 67.6, 44.9, 39.7, 21.2, 20.9; **HRMS-**

NSI: $C_{17}H_{16}N_2O_4$+[M+H]+ requires m/z 313.1183, found 313.1186.

3-methyl-1-(quinolin-8-yl)-3-(trifluoromethyl)pyrrolidine-2,5-dione (7i)

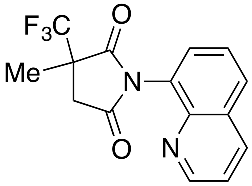

Following General Procedure E, 3,3,3-trifluoro-2,2-dimethyl-*N*-(quinolin-8-yl)propanamide **1i** (56 mg, 0.20 mmol) and $Co(acac)_2$ (5mg, 0.02 mmol) yielded product, which was purified by column chromatography (EtOAc) to provide the title compound as a colourless oil and as a mixture of diastereoisomers in ratio 3:1 (A:B). (49 mg, 0.16 mmol, 80%); **^{1}H NMR** (400 MHz, $CDCl_3$) δ: 8.87 (dd, *J*= 4.2, 1.7 Hz, 1H, diast. B), 8.55 (dd, *J*= 4.2, 1.7 Hz, 1H, diast. A), 8.23-8.16 (m, 1H, diast. A and B), 7.98-7.91 (m, 1H, diast. A and B), 7.67-7.60 (m, 2H, diast. A and B), 7.47-7.41 (m, 1H, diast. A and B), 3.47 (d, *J*= 18.4 Hz, 1H, diast. B), 3.32 (d, *J*= 18.4 Hz, 1H, diast. A), 2.98 (d, *J*= 18.4 Hz, 1H, diast. A), 2.84 (d, *J*= 18.4 Hz, 1H, diast. B), 1.86 (s, 3H, diast. A), 1.76 (s, 3H, diast. B); **^{13}C NMR** (100 MHz, $CDCl_3$) diast A δ: 173.5 (q, J_{C-F}= 1.8 Hz), 173.1, 151.3, 143.4, 136.4, 130.3, 129.7, 129.4, 129.2, 126.3, 125.6 (q, J_{C-F}= 280.0 Hz), 122.3, 49.9 (q, J_{C-F}= 27.7 Hz), 38.2, 19.0 (q, J_{C-F}= 2.0 Hz); **^{19}F NMR** (376 MHz, $CDCl_3$) diast A δ: -75.2; **^{13}C NMR** (100 MHz, $CDCl_3$) diast B δ: 173.5 (q, J_{C-F}= 1.8 Hz), 172.9, 151.5, 143.5, 136.0, 130.3, 129.6, 129.3, 126.0, 129.3, 126.0, 125.4 (q, J_{C-F}=282.0 Hz), 122.3, 49.9 (q, J_{C-F}= 28.3 Hz), 38.2, 19.3 (q, J_{C-F}= 2.5 Hz); **^{19}F NMR** (376 MHz, $CDCl_3$) diast B δ: -75.6. **HRMS-NSI**: $C_{15}H_{12}F_3N_2O_2^+$ $[M+H]^+$ requires m/z 309.0845, found 309.0845. All data consistent with reported compound.[1]

2-(3-methyl-2,5-dioxo-1-(quinolin-8-yl)pyrrolidin-3-yl)isoindoline-1,3-dione (7j)

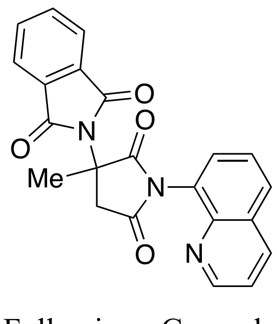

Following General Procedure E, 2-(1,3-dioxoisoindolin-2-yl)-2-methyl-*N*-(quinolin-8-yl)propanamide**1j** (72 mg, 0.20 mmol) and $Co(acac)_2$ (10 mg, 0.04 mmol) yielded product,

which was purified by column chromatography (EtOAc:MeOH 10:1) to provide the title compound as a white solid and as a mixture of diastereoisomers in ratio 16:1 (A:B) (40 mg, 0.10 mmol, 52%); **melting point**: 227-229 °C; **IR** ν max/cm^{-1} (neat film): 3069, 2924, 2852, 1779, 1712, 1501, 1373, 1208, 793, 718; **^{1}H NMR** (400 MHz, $CDCl_3$) diast. A δ: 8.89 (dd, *J*= 4.2, 1.4 Hz, 1H), 8.22 (d, *J*= 8.0 Hz, 1H), 7.96 (dd, *J*= 8.3, 1.0 Hz, 1H), 7.91 (d, *J*= 7.2 Hz, 1H), 7.86 (dd, *J*= 5.5, 3.1 Hz, 2H), 7.52 (dd, *J*= 5.5, 3.1 Hz, 2H), 7.69 (t, *J*= 8.0 Hz, 1H), 7.46 (dd, *J*= 8.3, 4.2 Hz, 1H), 3.39 (s, 2H), 2.44 (s, 3H); **^{13}C NMR** (100 MHz, $CDCl_3$) diast A δ: 176.6, 173.0, 168.5, 151.1, 143.7, 136.6, 134.7, 131.7, 130.2, 130.0, 129.7, 129.3, 126.6, 123.7, 122.2, 60.2, 43.8, 23.2.

3-(quinolin-8-yl)-3-azabicyclo[3.1.0]hexane-2,4-dione (7k)

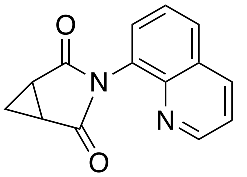

Following General Procedure E, *N*-(quinolin-8-yl)cyclopropanecarboxamide **1k** (42 mg, 0.20 mmol) and $Co(acac)_2$ (5 mg, 0.02 mmol) yielded product, which was purified by column chromatography (PE:EtOAc 1:1 – 0:1) to provide the title compound as a white solid and as a mixture of diastereoisomers in ratio 5.6:1 (A:B) (39 mg, 0.164 mmol, 82%); **melting point**: 192-193 °C; **IR** ν max/cm^{-1} (neat film): 3069, 2924, 2852, 1777, 1705, 1498, 1395, 1182, 827, 791; **^{1}H NMR** (400 MHz, $CDCl_3$) diast. A and B δ: 8.93 (dd, *J*= 4.2, 1.6 Hz, 1H, diast. B), 8.83 (dd, *J*= 4.2, 1.6 Hz, 1H, diast. A), 8.21 (d, *J*= 8.3 Hz, 1H, diast. B), 8.16 (dd, *J*= 8.3, 1.6 Hz, 1H, diast. A), 7.92 (d, *J*= 8.3 Hz, 1H, diast. B), 7.88 (dd, *J*= 8.2, 1.4 Hz, 1H, diast. A), 7.67 (dd, *J*= 7.3, 1.5 Hz, 1H, diast. A), 7.64-7.55 (m, 1H diast. A and 1H diast. B), 7.48-7.43 (m, 2H, diast. B), 7.41 (dd, *J*= 8.3, 4.2 Hz, 1H, diast. A), 2.78 (dd, *J*= 8.1, 3.6 Hz, 2H, diast. B), 2.69 (dd, *J*= 8.1, 3.6 Hz, 2H, diast. A), 2.45 (app. q, *J*= 3.6 Hz, 1H, diast. A), 1.78-1.70 (m, 2H, diast. B), 1.68 (td, *J*= 8.1, 4.3 Hz, 1H, diast. A); **^{13}C NMR** (100 MHz, $CDCl_3$) diast A and B δ: 175.0, 151.2, 151.0, 143.6, 136.3, 130.8, 130.1, 129.5, 129.3, 129.2, 129.1, 126.3, 122.2, 122.1, 21.5, 21.1, 21.0, 20.2; **HRMS-NSI**: $C_{14}H_{11}N_2O_2^+$ $[M+H]^+$ requires m/z 239.0815, found 239.0816.

1-methyl-3-(quinolin-8-yl)-3-azabicyclo[3.1.0]hexane-2,4-dione (7l)

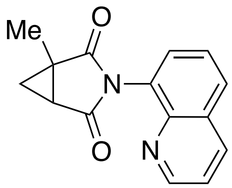

Following General Procedure E, 1-methyl-*N*-(quinolin-8-yl)cyclopropane-1-carboxamide **1l** (45 mg, 0.20 mmol) and Co(acac)$_2$ (10 mg, 0.04 mmol) yielded product, which was purified by column chromatography (PE:EtOAc 1:1 – 0:1) to afford the title compound as a yellow oil and as a mixture of diastereoisomers in ratio 5.2:1 (A:B) (25 mg, 0.10 mmol, 50%); **IR** ν max/cm^{-1} (neat film): 3069, 2924, 2852, 1778, 1710, 1397, 1150, 899, 793; **^{1}H NMR** (400 MHz, CDCl$_3$) δ: 8.91 (dd, 4.2, 1.6 Hz, 1H, diast. B), 8.83 (dd, *J*= 4.2, 1.6 Hz, 1H, diast. A), 8.19 (dd, *J*= 8.0, 1.5 Hz, 1H, diast. B), 8.16 (dd, *J*= 8.3, 1.5 Hz, 1H, diast. A), 7.93-7.89 (m, 1H, diast. B), 7.88 (dd, *J*= 8.2, 1.5 Hz, 1H, diast. A), 7.69-7.55 (m, 2H diast. A and 1H diast. B), 7.47-7.40 (m, 2H, diast. B), 7.40 (dd, *J*= 8.3, 4.2 Hz, 1H, diast. A), 2.58 (dd, *J*= 8.1, 3.4 Hz, 1H, diast. B), 2.56 (app. t, *J*= 3.7 Hz, 1H, diast. A), 2.49 (dd, *J*= 8.1, 3.4 Hz, 1H, diast. A), 1.87-1.83 (m, 1H, diast. B), 1.67 (s, 3H, diast. B), 1.64 (s, 3H, diast. A), 1.57-1.53 (m, 1H, diast. B), 1.53 (dd, *J*= 8.0, 4.1 Hz, 1H, diast. A); **^{13}C NMR** (100 MHz, CDCl$_3$) diast A and B δ: 177.3, 177.2, 175.2, 175.1, 151.3, 150.9, 143.8, 136.4, 136.3, 130.7, 130.1, 130.0, 129.6, 129.5, 129.3, 129.0, 126.2, 122.1, 122.0, 27.9, 27.8, 27.6, 27.2, 27.0, 26.8, 13.3, 13.1.

HRMS-NSI: $C_{15}H_{13}N_2O_2^+$ $[M+H]^+$ requires m/z 253.0974, found 253.0972.

2-(2,4-dioxo-3-(quinolin-8-yl)-3-azabicyclo[3.1.0]hexan-1-yl)isoindoline-1,3-dione (7m)

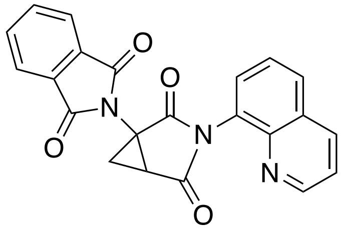

Following General Procedure E, 1-(1,3-dioxoisoindolin-2-yl)-*N*-(quinolin-8-yl)cyclopropane-1-carboxamide **1m** (71 mg, 0.20 mmol) and Co(acac)$_2$ (10 mg, 0.04 mmol) yielded product, which was purified by column chromatography (CH_2Cl_2:MeOH 20:1) to afford the title compound as a white solid and as a mixture of diastereomers in ratio 10:1 (38 mg, 0.098 mmol, 48%); **melting point**: 208-212 °C; **IR** ν max/cm^{-1} (neat film): 3103, 1781, 1716, 1498, 1477, 1427, 1399, 1358, 1313, 1247; **^{1}H NMR** (400 MHz,

$CDCl_3$) δ: 8.86 (dd, *J*= 4.3 Hz, 1.6 Hz), 1H, 8.17 (dd, *J*= 8.4 Hz, 1.6 Hz, 1H), 7.94-7.87 (m, 3H), 7.80-7.75 (m, 3H), 7.64-7.59 (m, 1H), 7.45-7.40 (m, 2H), 3.19-3.15 (m, 1H), 2.99-2.94 (m, 1H), 2.48 (dd, *J*= 8.8 Hz, 5.8 Hz, 1H); **^{13}C NMR** (100 MHz, $CDCl_3$) δ: 172.4, 171.3, 167.4, 151.1, 143.5, 136.3, 134.9, 131.7, 130.9, 129.8, 192.2, 128.9, 126.3, 124.1, 122.2, 37.8, 25.6, 24.4;

6,6-diphenyl-3-(quinolin-8-yl)-3-azabicyclo[3.1.0]hexane-2,4-dione (7n)

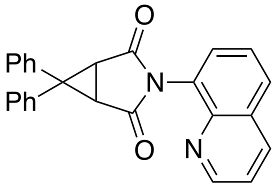

Following General Procedure E, 2,2-diphenyl-*N*-(quinolin-8-yl)cyclopropane-1-carboxamide **1n** (73 mg, 0.20 mmol) and $Co(acac)_2$ (10 mg, 0.04 mmol) yielded product, which was purified by column chromatography (PE:EtOAc 4:1 – 7:3) to afford the title compound as a white solid and as a mixture of diastereomers in ratio 24:1 (44 mg, 0.11 mmol, 56%); **melting point:** 140-142 °C; **IR** ν max/cm^{-1} (neat film): 2927, 2851, 1715, 1462, 1275; **^{1}H NMR** (400 MHz, $CDCl_3$) δ: 8.85 (dd, *J*= 4.3 Hz, 1.7 Hz, 1H), 8.06 (dd, *J*= 8.3 Hz, 1.7 Hz, 1H), 7.70 (dd, *J*= 8.3 Hz, 0.8 Hz, 1H), 7.61-7.56 (m, 2H), 7.42-7.37 (m, 3H), 7.36-7.32 (m, 1H), 7.30-7.23 (m, 4H), 7.22-7.17 (m, 2H), 5.53 (dd, *J*= 7.2 Hz, 1.3 Hz, 1H), 3.40 (s, 2H); **^{13}C NMR** (100 MHz, $CDCl_3$) δ: 172.7, 151.3, 144.7, 141.5, 137.0, 136.4, 130.3, 129.8, 129.6, 129.3, 129.2, 129.2, 128.6, 128.3, 128.0, 127.2, 126.2, 122.0; **HRMS-NSI**: $C_{26}H_{19}O_2N_2$+ [M+H]+ requires m/z 391.1441, found 391.1439.

3-methyl-1-(quinolin-8-yl)pyrrolidine-2,5-dione (7o)

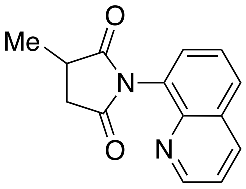

Following General Procedure E, *N*-(quinolin-8-yl)isobutyramide **1o** (43 mg, 0.20 mmol) and $Co(acac)_2$ (5 mg, 0.02 mmol) yielded product, which was purified by column chromatography (PE:EtOAc 1:1 – 0:1) to provide the title compound as a white powder and as a mixture of diastereoisomers in ratio 1.1:1 (A:B) (32 mg, 0.13 mmol, 66%); **melting point**: 122-124 °C; **IR** ν max/cm^{-1} (neat film): 2965, 2927, 2852, 1701, 1496, 1474, 1397, 1200, 1177; **^{1}H NMR** (400 MHz, $CDCl_3$) δ: 8.89 (m, 1H, diast. A and B), 8.22-8.15 (m,

1H, diast. A and B), 7.95-7.89 (m, 1H, diast. A and B), 7.66-7.59 (m, 2H, diast. A and B), 7.46-7.40 (m, 1H, diast. A and B), 3.37-3.26 (m, 2H, diast. A), 3.20-3.08 (m, 2H, diast. B), 2.81-2.69 (m, 1H, diast. B), 2.65-2.55 (m, 1H, diast. A), 1.59 (d, *J*= 7.1 Hz, 3H, diast. B), 1.51 (d, *J*= 7.0, 3H, diast. A); **^{13}C NMR** (100 MHz, $CDCl_3$) diast. A and B δ: 180.5, 180.4, 176.5, 176.2, 151.2, 151.0, 143.7, 143.6, 136.5, 136.3, 130.5, 130.3, 129.9, 129.8, 129.7, 129.6, 129.5, 126.3, 126.3, 122.1, 37.3, 37.3, 35.8, 35.6, 17.4, 17.0; **HRMS-NSI**: $C_{14}H_{13}N_2O_2^+$ $[M+H]^+$ requires m/z 241.0972, found 241.0969.

2-(2,5-dioxo-1-(quinolin-8-yl)pyrrolidin-3-yl)isoindoline-1,3-dione (7p)

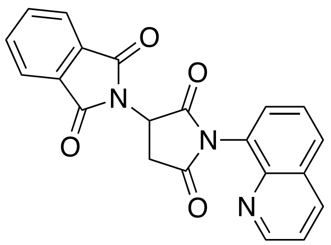

Following general procedure E, 2-(1,3-dioxoisoindolin-2-yl)-*N*-(quinolin-8-yl)propanamide **1p** (69 mg, 0.20 mmol) and $Co(acac)_2$ (10 mg, 0.04 mmol) yielded product, which was purified by column chromatography (CH_2Cl_2) to afford the title compound as a yellow oil and as a mixture of diastereoisomers in ratio 9:1 (A:B) (41 mg, 0.11 mmol, 55%); **IR** ν max/cm^{-1} (neat film): 3059, 2929, 1776, 1709, 1501, 1469, 1382, 1207; **^{1}H NMR** (400 MHz, $CDCl_3$) δ: 9.04 (dd, *J*= 4.1, 1.4 Hz, 1H, diast. B), 8.90 (dd, *J*= 4.3, 1.4 Hz, 1H, diast. A), 8.24 (d, *J*= 8.0 Hz, 1H, diast. A), 8.19 (d, *J*= 8.4, 1.4 Hz, 1H, diast. B), 7.99-7.65 (m, 8H, diast. A and diast. B), 5.71 (dd, *J*= 9.9, 5.8 Hz, 1H, diast. A), 5.57 (dd, *J*= 9.4, 7.5 Hz, 1H, diast. B), 3.62 (dd, *J*= 17.3, 7.5 Hz, 1H, diast. B), 3.56 (dd, *J*= 17.8, 9.9 Hz, 1H, diast. A), 3.34 (dd, *J*= 9.4, 17.3 Hz, 1H, diast. B), 3.19 (dd, *J*= 17.8, 5.8 Hz, 1H, diast. A); **^{13}C NMR** (100 MHz, $CDCl_3$) diast. A δ: 173.5, 173.1, 134.9, 134.8, 134.5, 131.9, 130.3, 130.2, 129.5, 126.7, 124.1, 123.8, 122.3, 47.6, 35.0; **HRMS-NSI**: $C_{21}H_{14}N_3O_4^+$$[M+H]^+$ requires m/z 372.07979, found 372.984.

Product Derivatisations

2-methyl-2-phenylsuccinic acid (8)

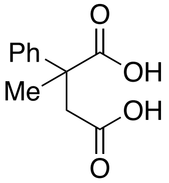

A solution of 3-methyl-3-phenyl-1-(quinolin-8-yl)pyrrolidine-2,5-dione **2c** (200 mg, 0.63 mmol) in concentrated HCl (3 mL) was stirred at 120 °C in a sealed vial for 72 h. The mixture was basified with 10% aqueous NaOH and washed with Et_2O (3 × 15 mL). The aqueous layer was then acidified to pH 1 with 3M HCl, extracted with EtOAc (3 × 15 mL) dried (Mg_2SO_4) and concentrated *in vacuo* to afford the title compound as a white, crystaline solid (114 mg, 0.55 mmol, 88%); **melting point**: 150-152 °C; **IR** ν max/cm^{-1} (neat film): 3154, 1708, 1420, 1295; **^{1}H NMR** (400 MHz, MeOD) δ: 7.43-7.39 (m, 2H), 7.35-7.31 (m, 2H), 7.26-7.21 (m, 1H), 3.20 (d, *J*= 16.3 Hz, 1H), 2.82 (d, *J*= 16.3 Hz, 1H), 1.69 (s, 3H); **^{13}C NMR** (100 MHz, MeOD) δ: 178.9, 174.8, 144.8, 129.5, 128.0, 126.9, 44.4, 24.1.

2-methyl-4-morpholino-4-oxo-2-phenyl-N-(quinolin-8-yl)butanamide (9)

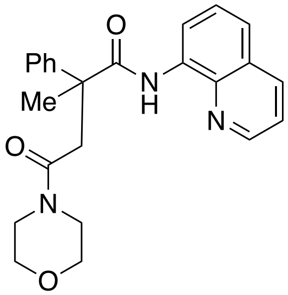

A solution of 3-methyl-3-phenyl-1-(quinolin-8-yl)pyrrolidine-2,5-dione **2c** (63 mg, 0.20 mmol) in morpholine (2 mL) was heated to 120 °C in a sealed vial for 16 h. The reaction was concentrated, and purified by column chromatography (PE:EtOAc 7:3) to afford the title compound as a colourless oil (62 mg, 0.15 mmol, 77%); **IR** ν max/cm^{-1} (neat film): 3363, 1704, 1675, 1647, 1527, 1486, 1425, 1385, 1326; **^{1}H NMR** (400 MHz, $CDCl_3$) δ: 10.19 (s, 1H), 8.80 (dd, *J*= 7.5 Hz, 1.4 Hz 1H), 8.66 (dd, *J*= 4.3 Hz, 1.7 Hz, 1H), 8.10 (8.3 Hz, 1.7 Hz), 7.56-7.44 (m, 4H), 7.40-7.34 (m, 3H), 7.30-7.26 (m, 1H), 3.63-3.28 (m, 9H), 3.00 (d, *J*= 15.4 Hz), 2.13 (s, 3H); **^{13}C NMR** (100 MHz, $CDCl_3$) δ: 174.5, 169.3, 148.4, 143.7, 138.9, 136.3, 134.8, 129.0, 128.0, 127.5, 127.4, 126.6, 121.6, 121.5, 116.3, 67.0,

66.6, 50.9, 46.4, 42.4, 41.9, 23.9; **HRMS-NSI**: $C_{24}H_{26}O_3N_3$+ $[M+H]^+$ requires m/z 404.1969, found 404.1962.

2-methyl-2-phenyl-4-(quinolin-8-ylamino)butan-1-ol (10)

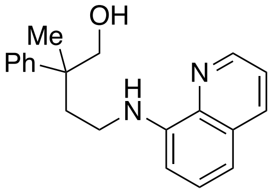

To a solution of 3-methyl-3-phenyl-1-(quinolin-8-yl)pyrrolidine-2,5-dione **2c** (63 mg, 0.20 mmol) in THF (2 mL) was added $LiAlH_4$ (38 mg, 1.0 mmol) portionwise at 0 °C. The reaction was allowed to warm to room temperature and was heated to reflux for 16 h. The reaction was diluted with CH_2Cl_2 (10 mL), washed with EtOAc (3 × 10 mL), dried (Mg_2SO_4) and concentrated to yield product, which was purified by column chromatography (PE:EtOAc 7:3-1:1) to afford the title compound as a yellow oil (41 mg, 0.13 mmol, 66%); **IR** ν max/cm^{-1} (neat film): 3397, 3048, 2926,1575, 1521, 1478, 1379, 1339; **^{1}H NMR** (400 MHz, $CDCl_3$) δ: 8.68 (dd, *J*= 4.27 Hz, 1.7Hz, 1H), 8.04 (dd, *J*= 8.3 Hz, 1.5 Hz, 1H), 7.46-7.24 (m, 8H), 7.01 (d, *J*= 8.2 Hz, 1H), 6.50 (d, J *J*= 8.2 Hz, 1H), 6.05 (br. s, 1H), 3.79 (d, *J*= 11.0 Hz, 1H), 3.67 (d, *J*= 11.0 Hz, 1H), 2.33-2.25 (m, 1H), 3.13-3.04 (m, 1H), 1.50 (3.47); **^{13}C NMR** (100 MHz, $CDCl_3$) δ:146.9, 144.8, 144.4, 138.4, 136.3, 128.9, 128.8, 128.0, 126.8, 126.7, 121.5, 113.9, 104.9, 72.6, 43.0, 39.3, 38.1, 22.1.

References:

1. X. Wu, Y. Zhao and H. Ge, *J. Am. Chem. Soc.,* 2015, **137**, 4924-4927
2. Y. Wang, Y. Wang, K. Jiang, Q. Zhiang and D. Li, *Org. Biomol. Chem.,* 2016, **14**, 10180-10184
3. D. S. Roman and A. B. Charette, *Org. Lett.,* 2013, **15**, 4394-4397
4. S. Yan, Y. Liu, B. Liu, Y. Liu, Z. Zhang and B. Shi, *Chem. Commun.,* 2015, **51**, 7341-7344
5. X. Wu, Y. Zhao and H. Ge, *Chem. Eur. J.,* 2014, **20**, 9530-9533
6. X. Wu, K. Yang, Y. Zhao, H. Sun, G. Li and H. Ge, *Nature Commun.,* 2015, **6**, 6462
7. M. Lei, H. Feng, C. Wang, H. Li, J. Shi, J. Wang, Z. Liu, S. Chen, S. Hu and Y. Zhu *Bioorg. Med. Chem.,* 2016, **24**, 2576-2588

8. R. Shang, L. Ilies, A. Matsumoto and E. Nakamura, *J. Am. Chem. Soc.*, 2013, **135**, 6030–6032

9. B. Li, S. Wang, B. Liu and B. Shi, *Org. Lett.,* 2015, **17**, 1200-1203

10. N. Hasegawa, V. Charra, S. Inoue, Y. Fukumoto and N. Chatani, *J. Am. Chem. Soc.*, 2011, **133**, 8070-8073

11. S. Zhang, X. Yang, X. Zhao, P. Li, J. Liu, M. Song, *Organometallics*, 2015, **34**, 4331-4339

12. M. Salamone, F. Basili, R. Mele, M. Cianfanelli and M. Bietti, *Org. Lett.*, 2014, **16**, 6444–7

***N*-(quinolin-8-yl)pivalamide (1a)**
^{1}H NMR ($CDCl_3$, 400 MHz)

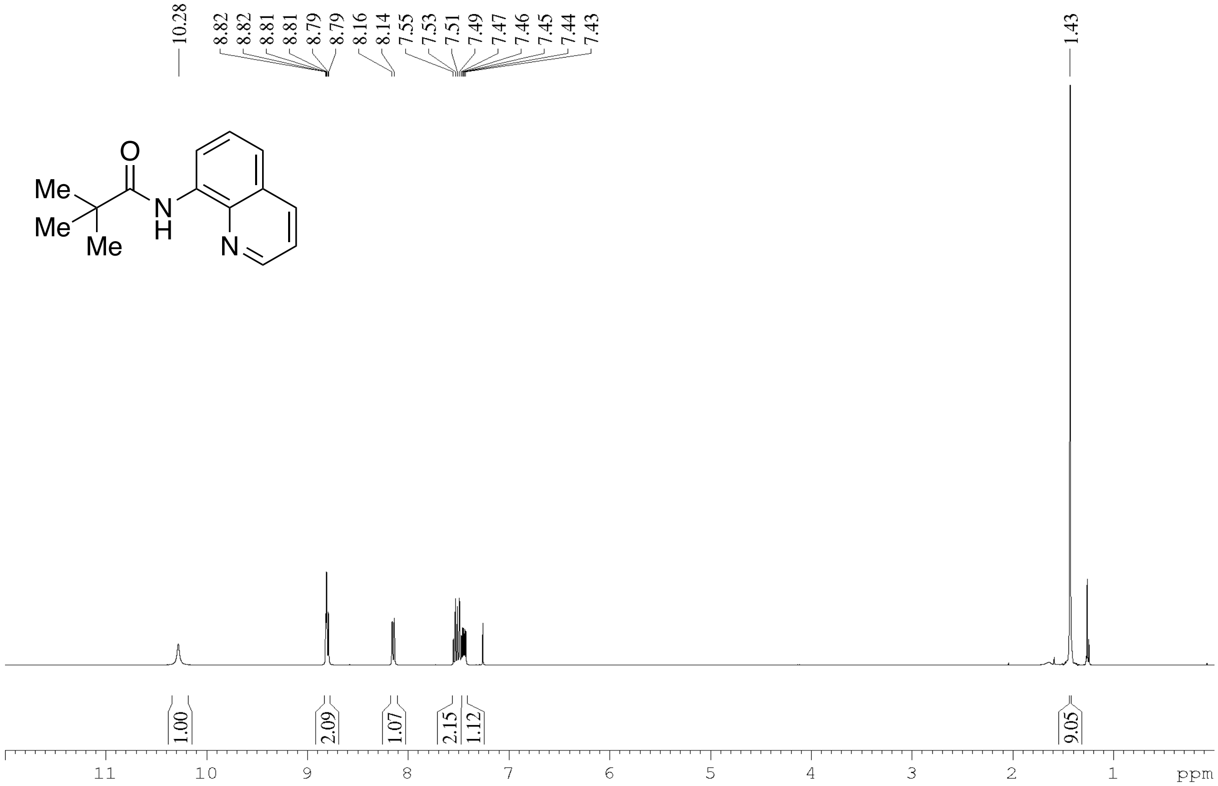

^{13}C NMR ($CDCl_3$, 100 MHz)

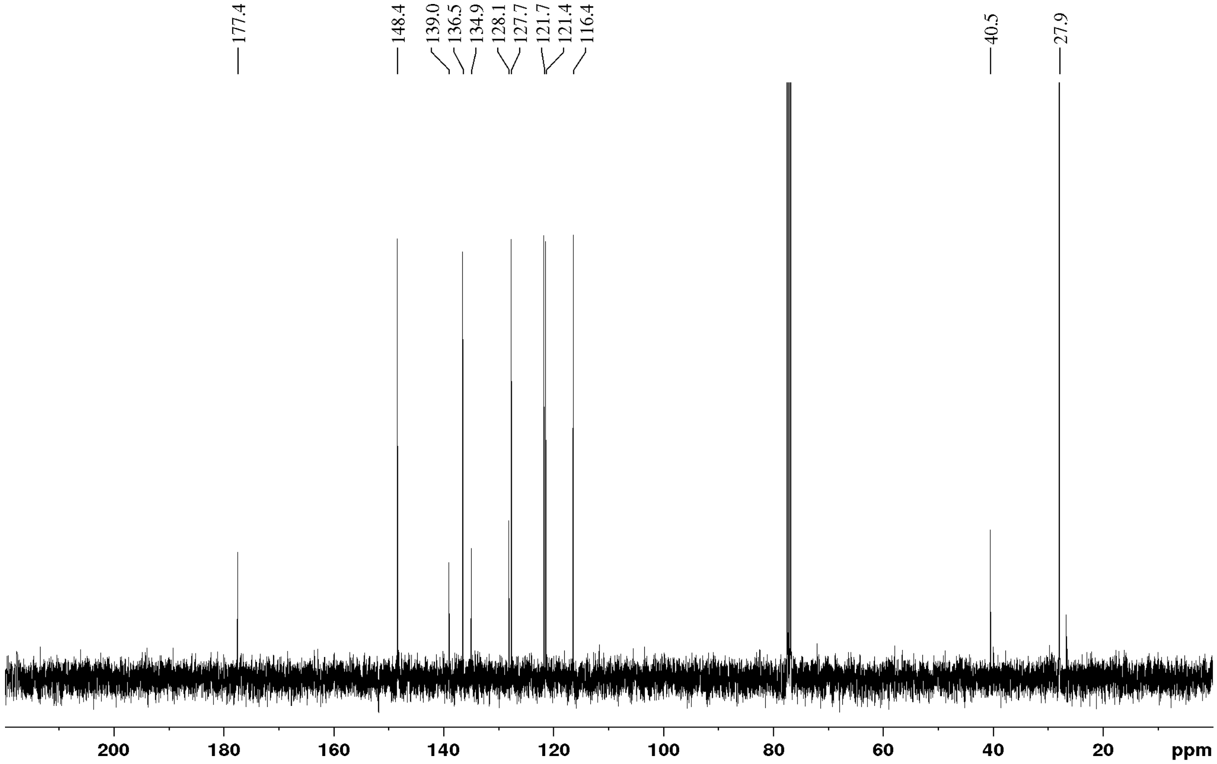

2,2-dimethyl-*N*-(quinolin-8-yl)butanamide (1b)

^{1}H NMR ($CDCl_3$, 400 MHz)

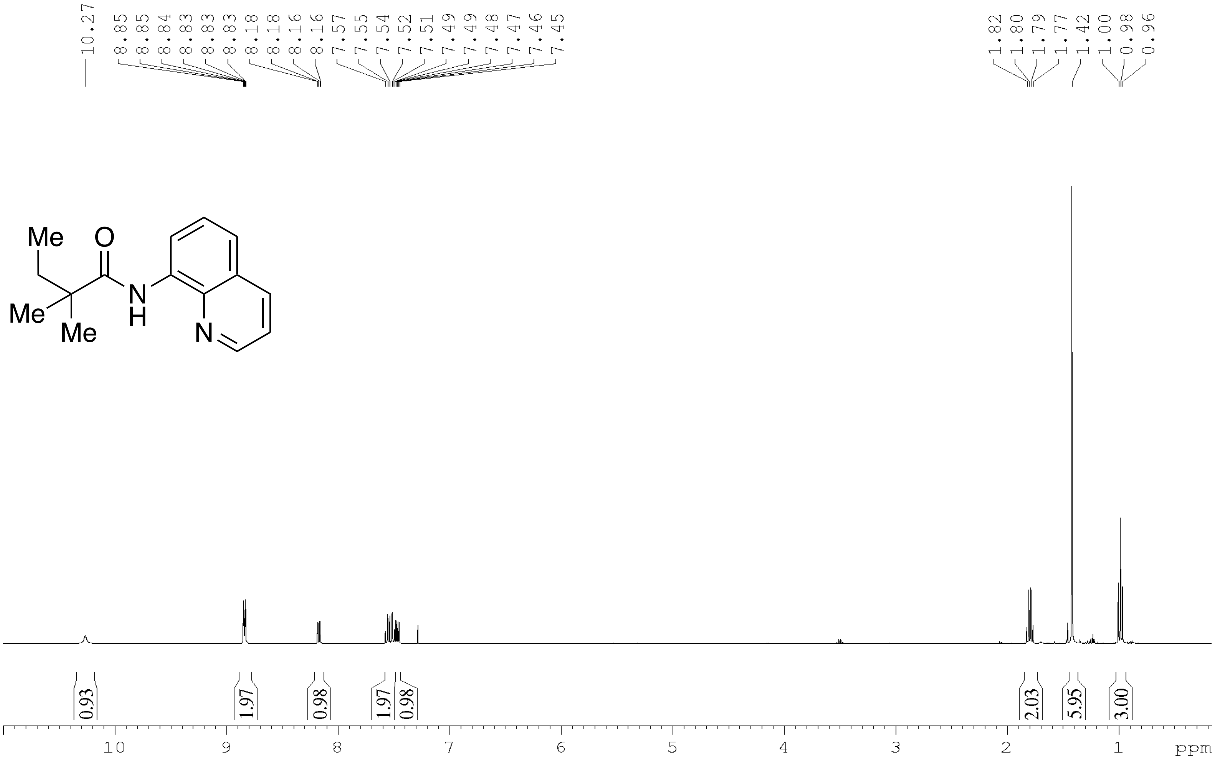

^{13}C NMR ($CDCl_3$, 100 MHz)

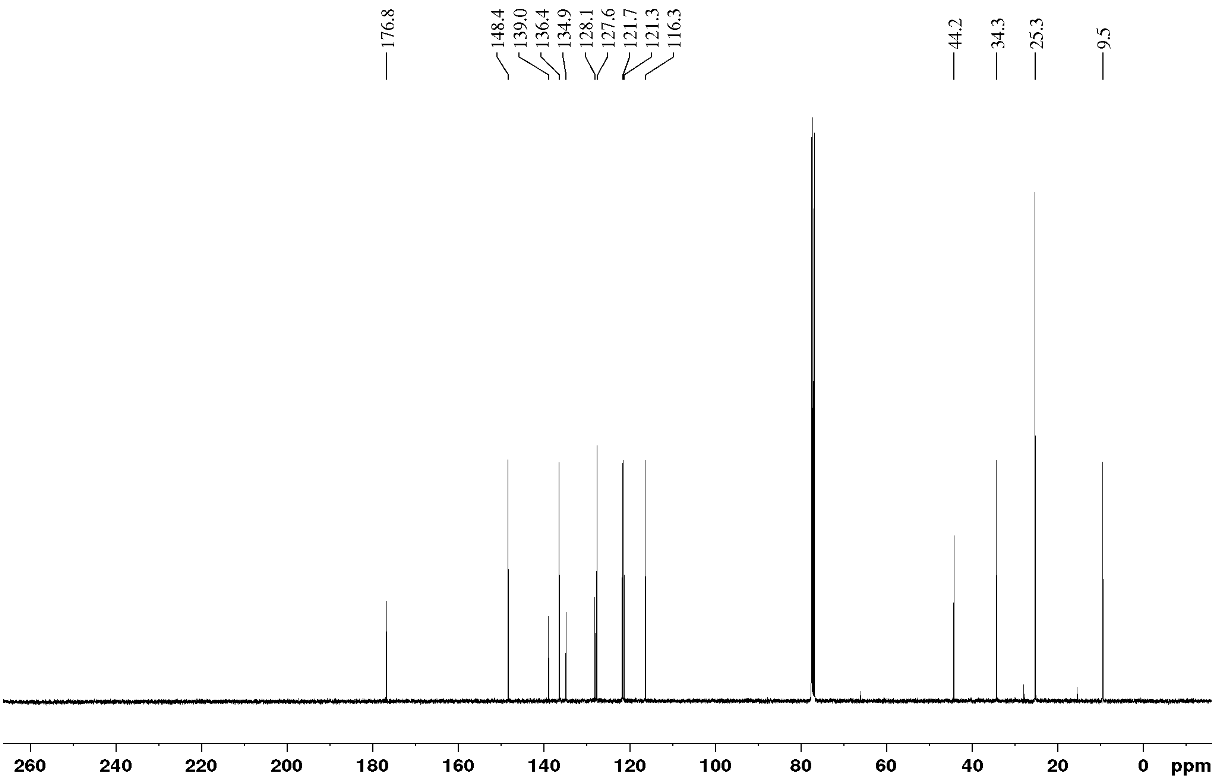

2-methyl-2-phenyl-*N*-(quinolin-8-yl)propanamide (1c)

^{1}H NMR ($CDCl_3$, 400 MHz)

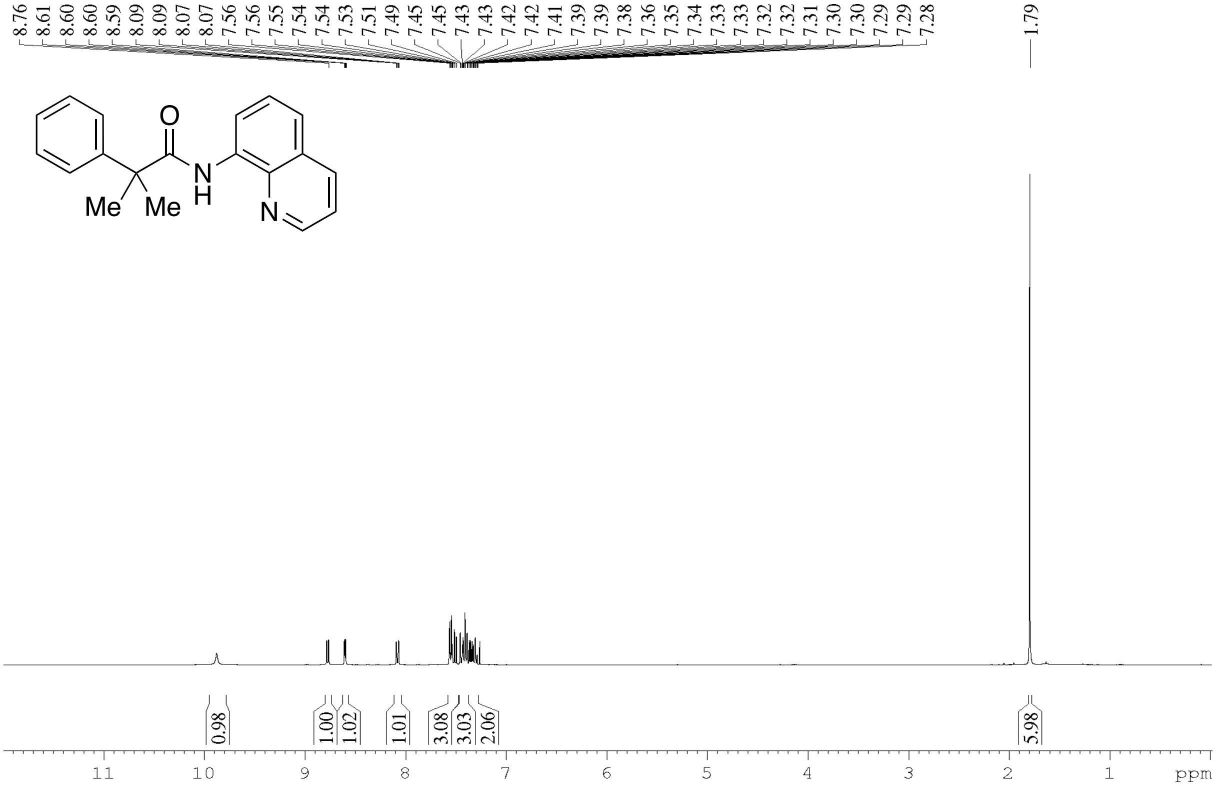

^{13}C NMR ($CDCl_3$, 100 MHz)

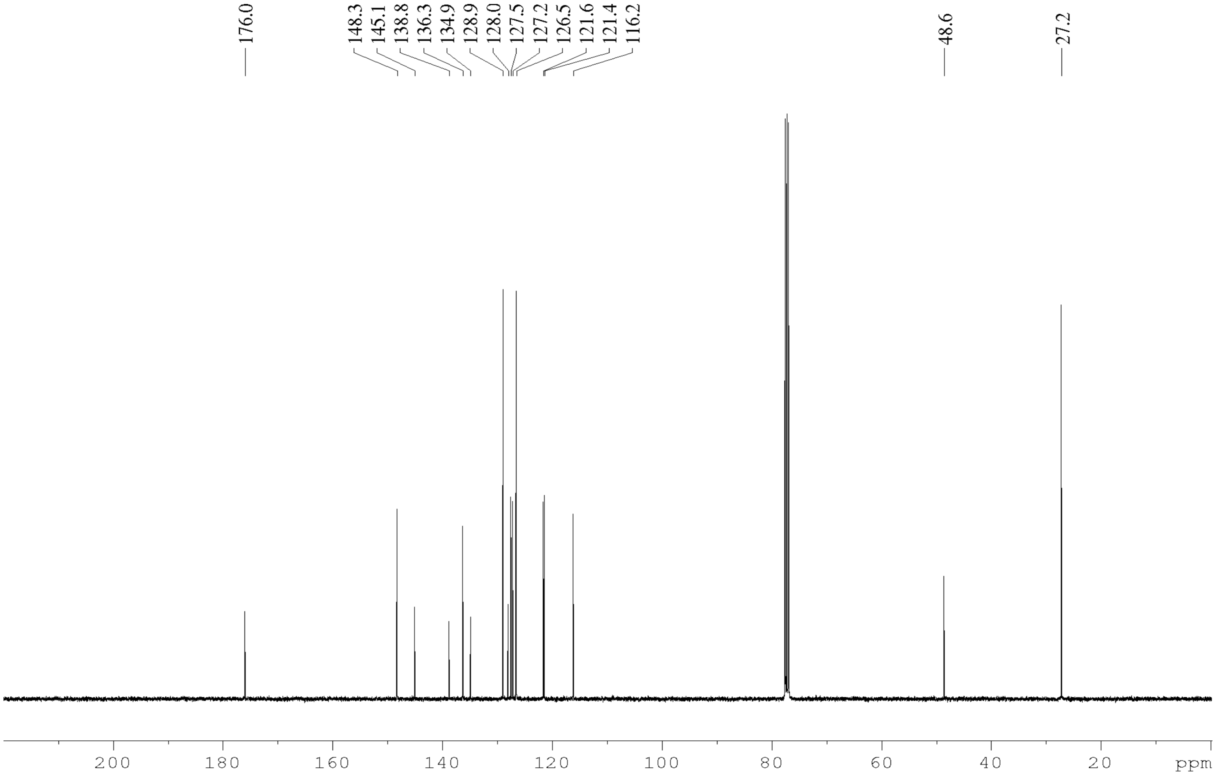

2,2-dimethyl-3-phenyl-*N*-(quinolin-8-yl)propanamide (1d)
^{1}H NMR ($CDCl_3$, 400 MHz)

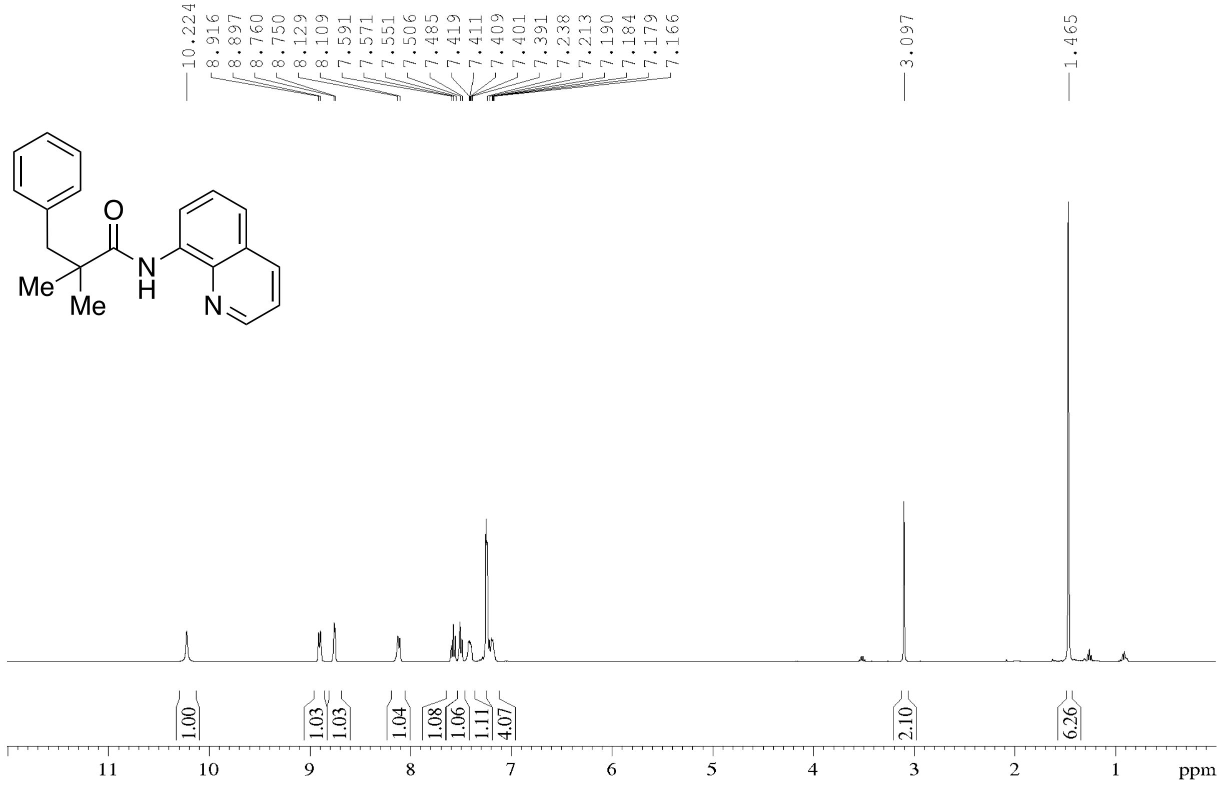

^{13}C NMR ($CDCl_3$, 100 MHz)

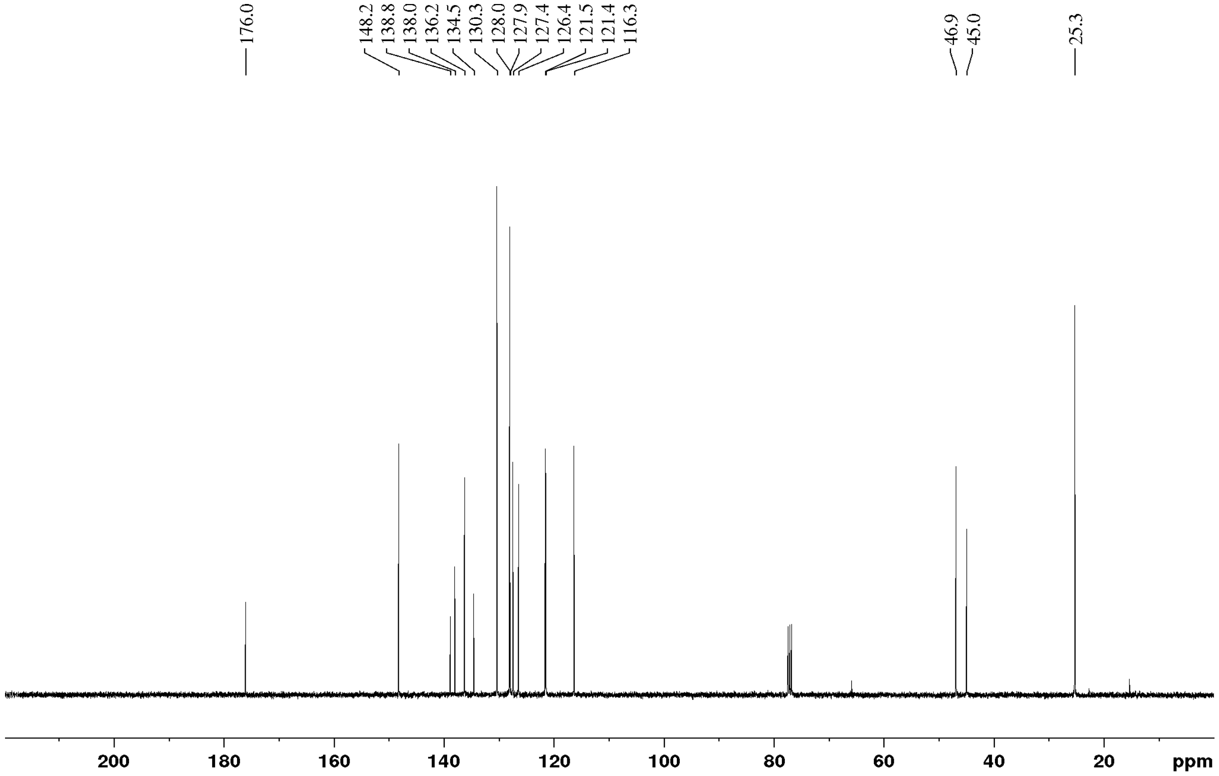

1-methyl-*N*-(quinolin-8-yl)cyclohexane-1-carboxamide (1e)

^{1}H NMR ($CDCl_3$, 400 MHz)

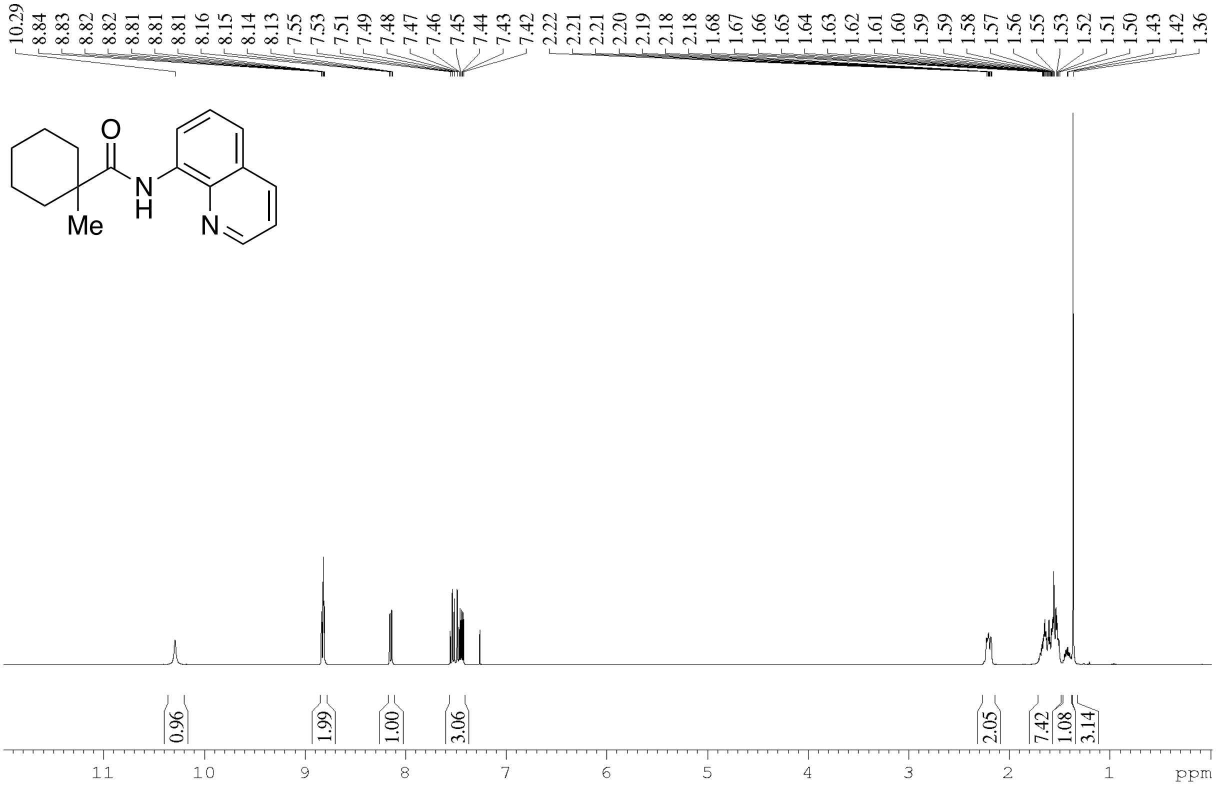

^{13}C NMR ($CDCl_3$, 100 MHz)

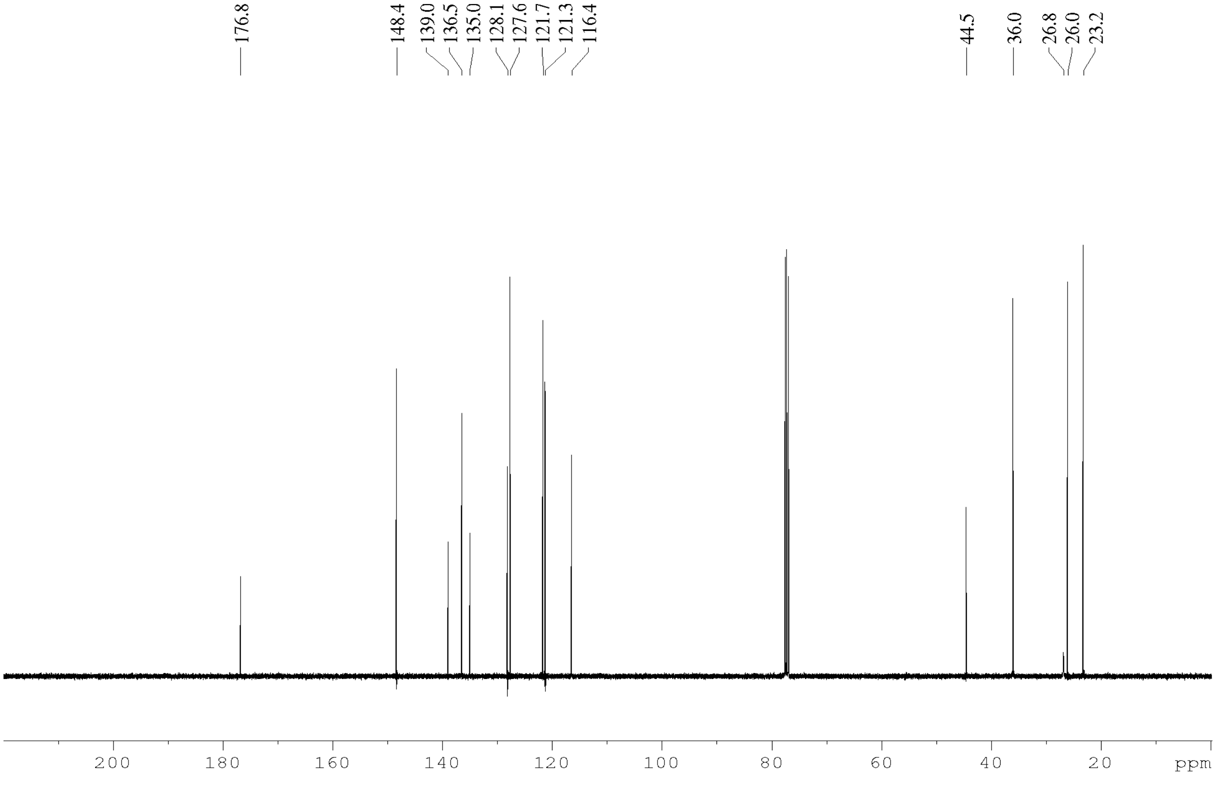

4-methyl-*N*-(quinolin-8-yl)-1-tosylpiperidine-4-carboxamide (1f)
^{1}H NMR ($CDCl_3$, 400 MHz)

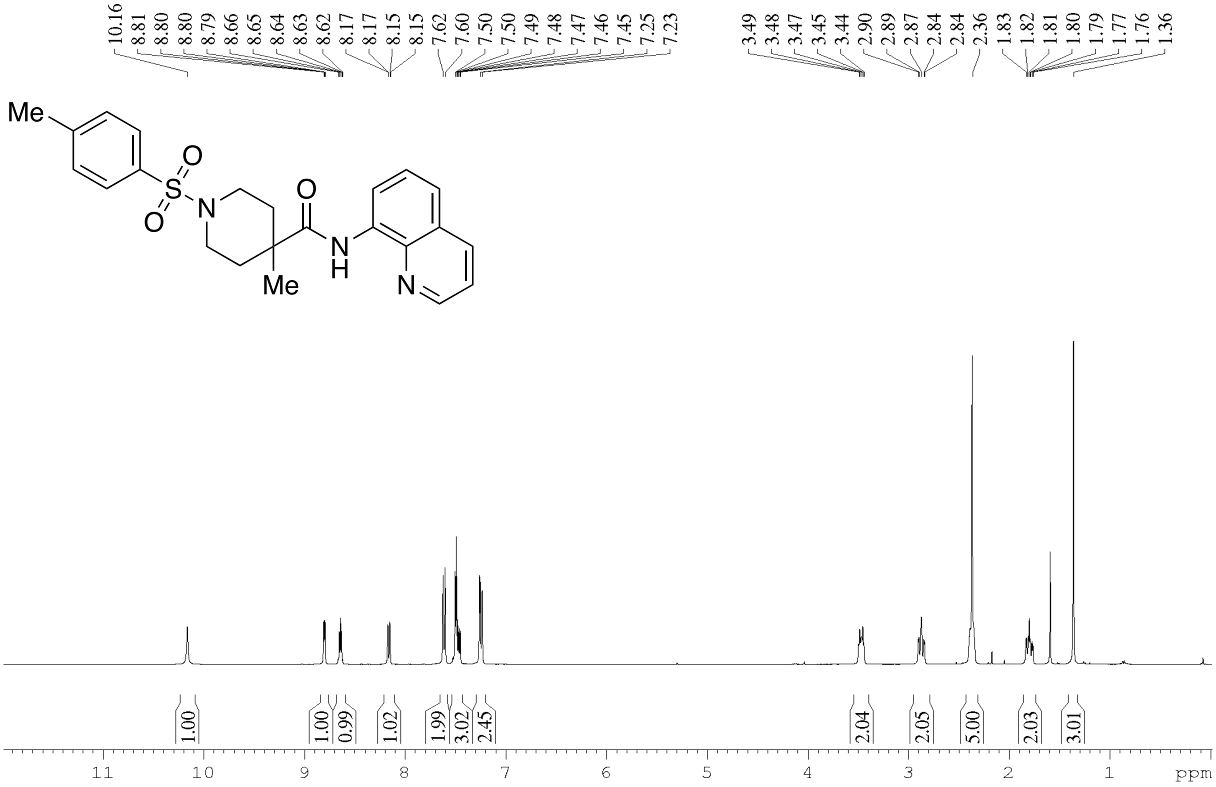

^{13}C NMR ($CDCl_3$, 100 MHz)

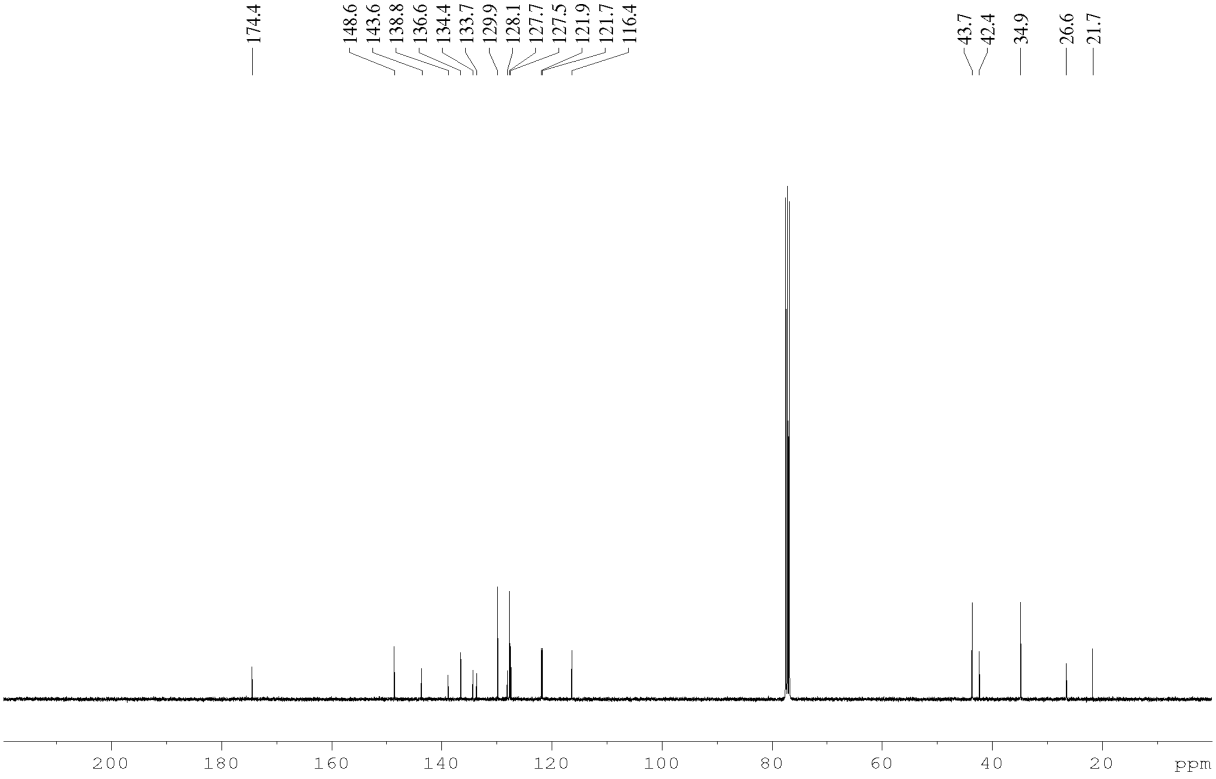

ethyl 2,2-dimethyl-3-oxo-3-(quinolin-8-ylamino)propanoate (1g)

^{1}H NMR ($CDCl_3$, 400 MHz)

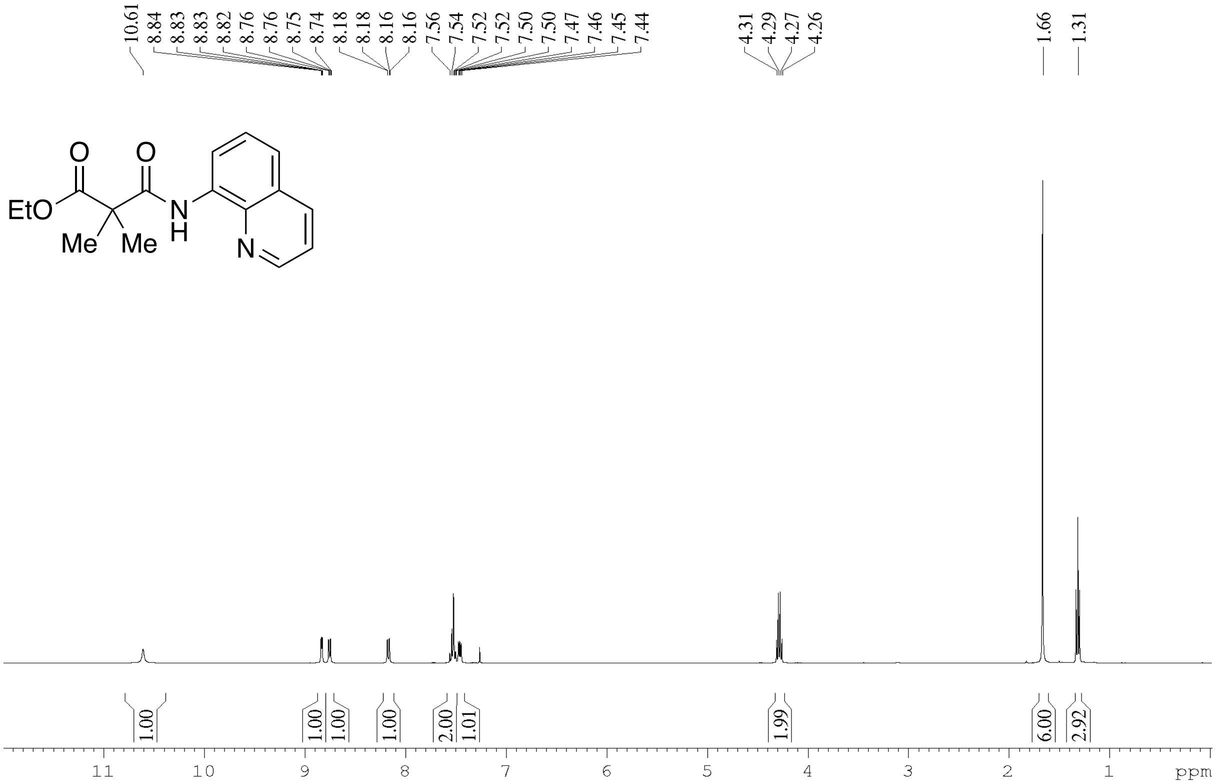

^{13}C NMR ($CDCl_3$, 100 MHz)

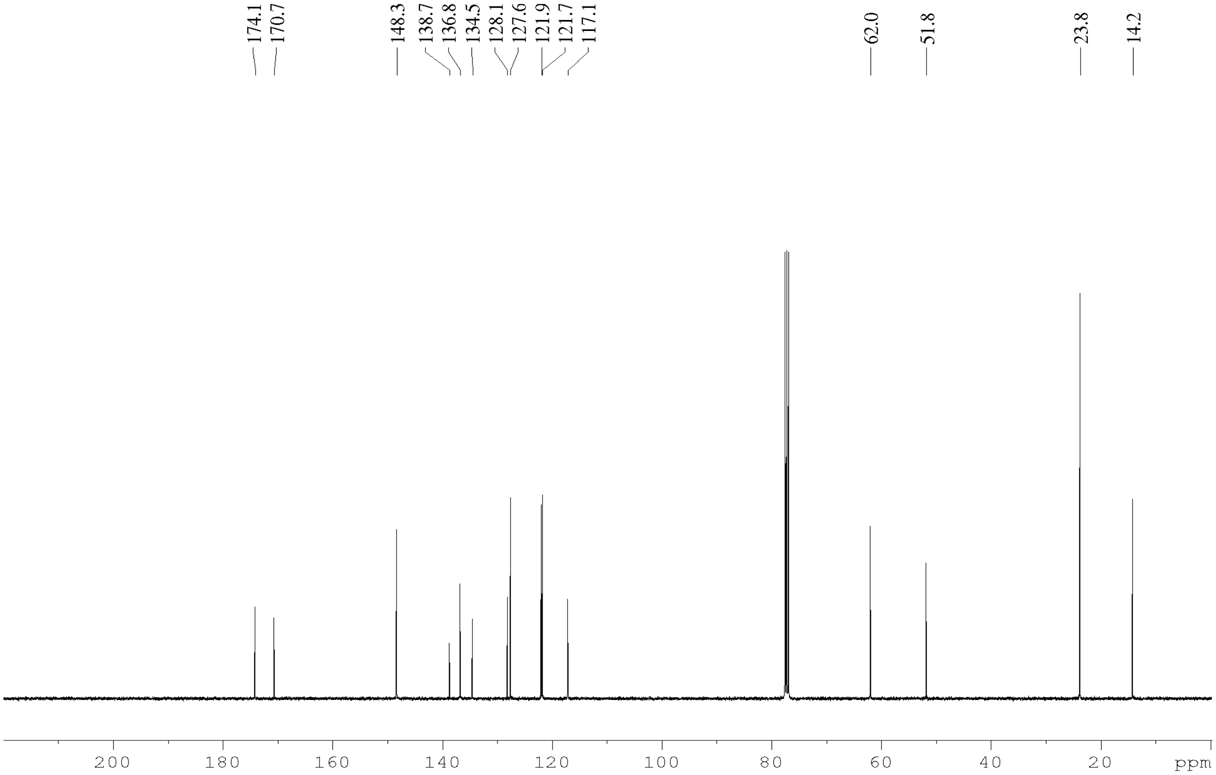

2,2-dimethyl-3-oxo-3-(quinolin-8-ylamino)propyl acetate (1h)

^{1}H NMR (CDCl$_3$, 400 MHz)

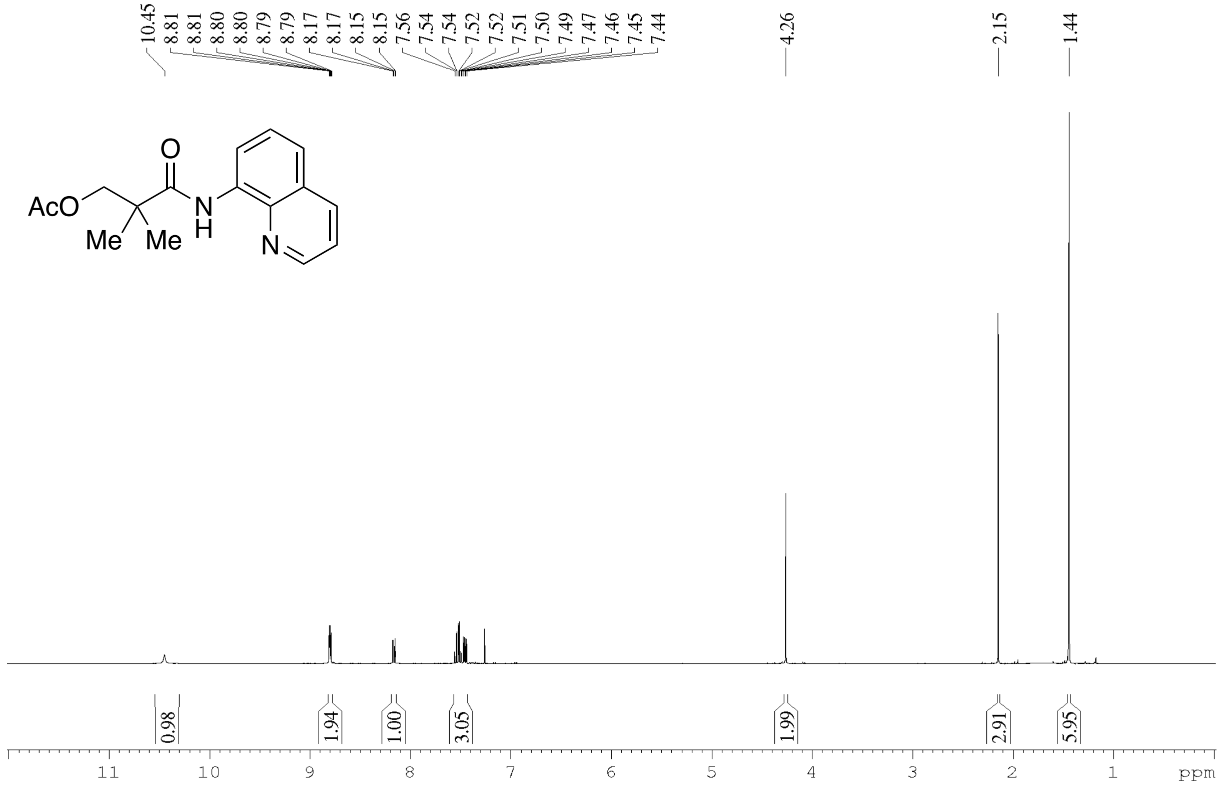

^{13}C NMR (CDCl$_3$, 100 MHz)

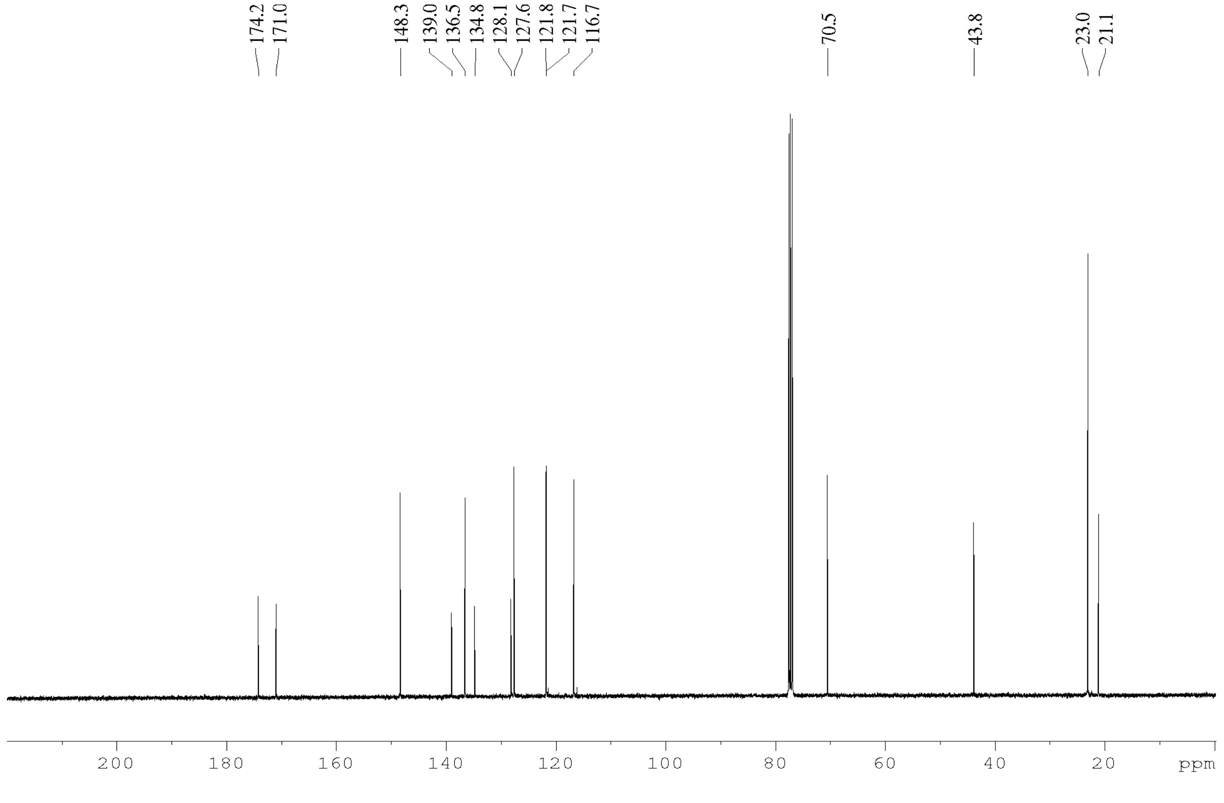

3,3,3-trifluoro-2,2-dimethyl-*N*-(quinolin-8-yl)propanamide (1i)

^{1}H NMR ($CDCl_3$, 400 MHz)

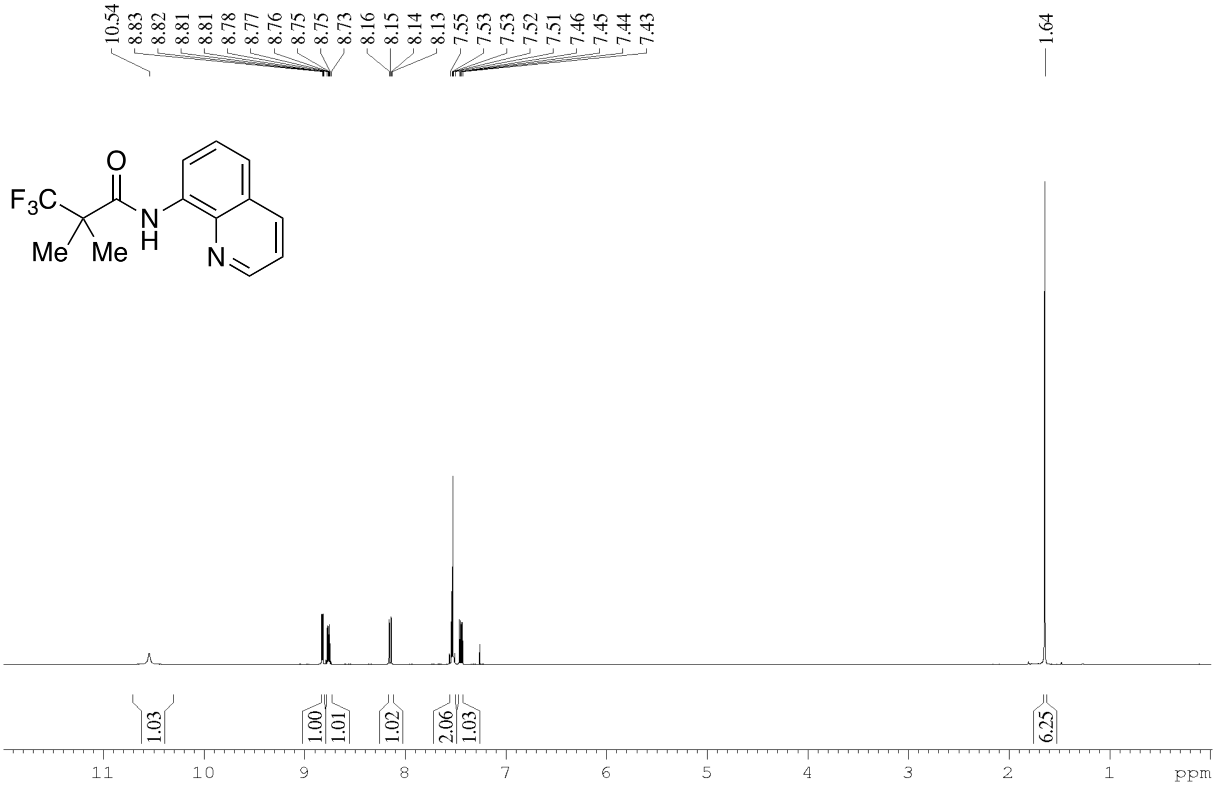

^{13}C NMR ($CDCl_3$, 100 MHz)

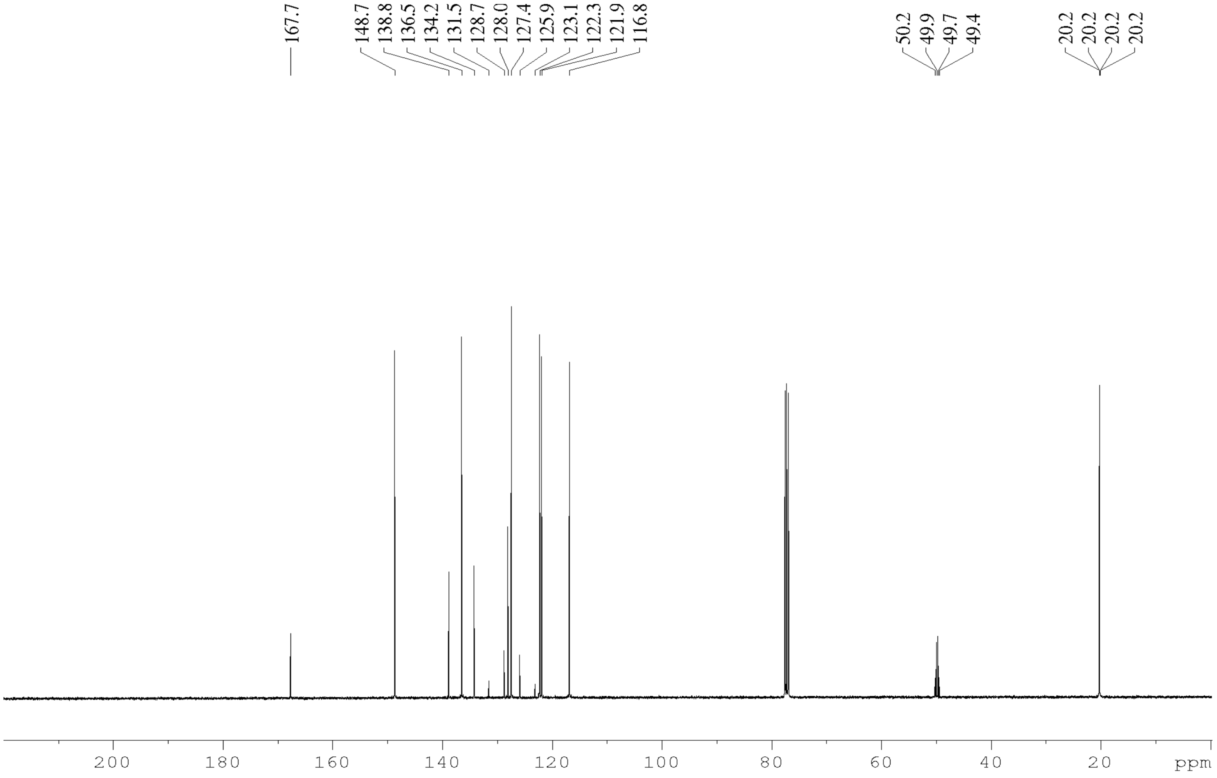

^{19}F NMR ($CDCl_3$, 376 MHz)

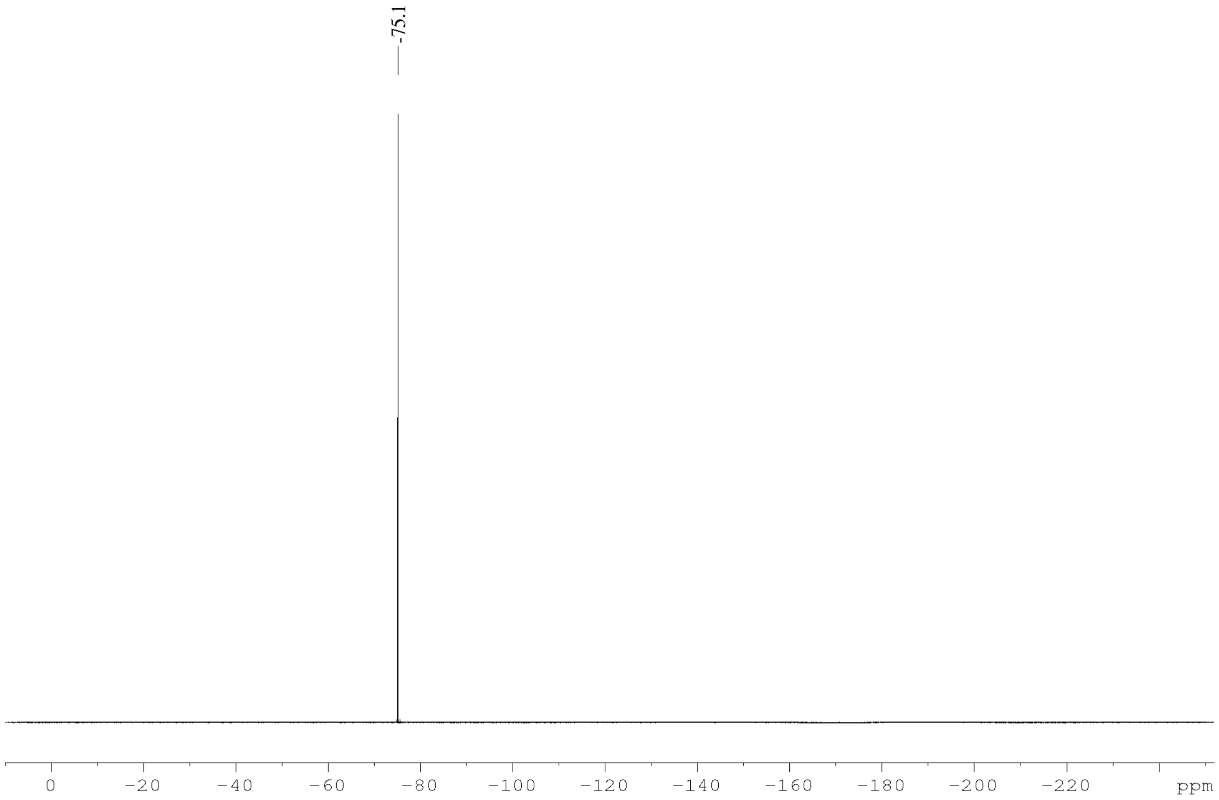

2-(1,3-dioxoisoindolin-2-yl)-2-methyl-*N*-(quinolin-8-yl)propanamide (1j)
^{1}H NMR ($CDCl_3$, 500 MHz)

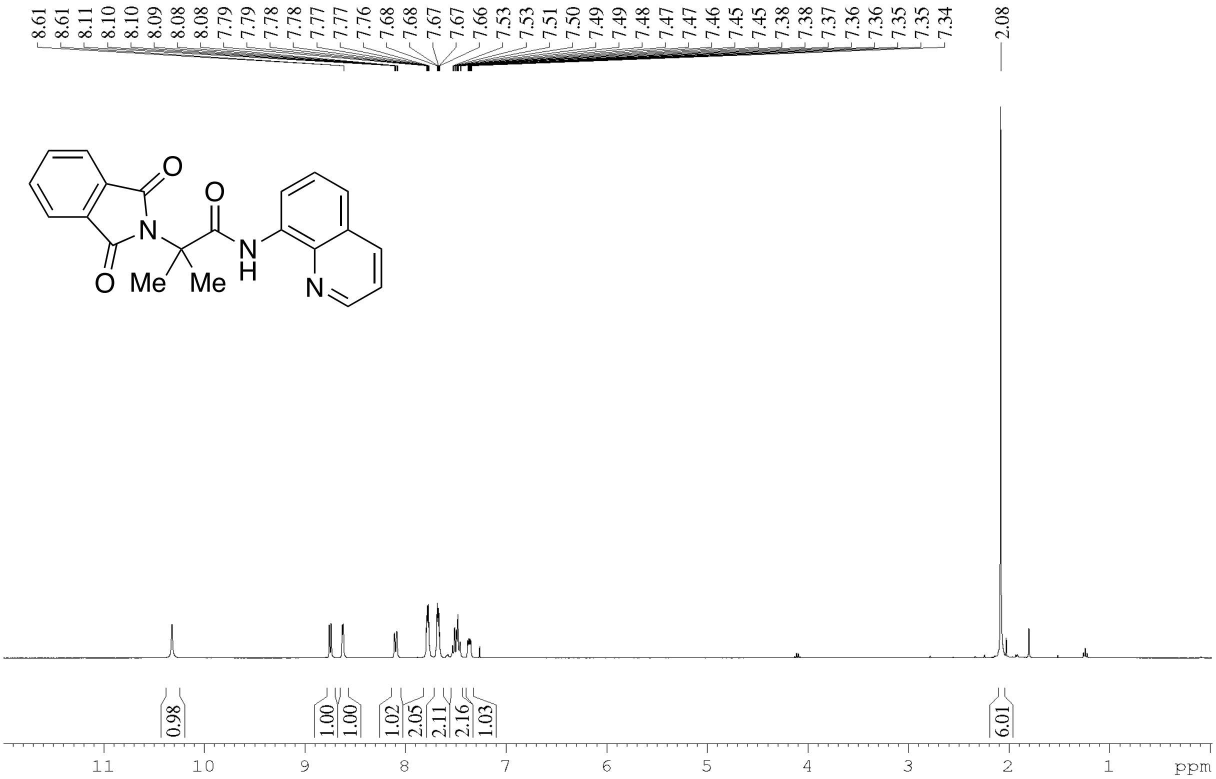

^{13}C NMR ($CDCl_3$, 126 MHz)

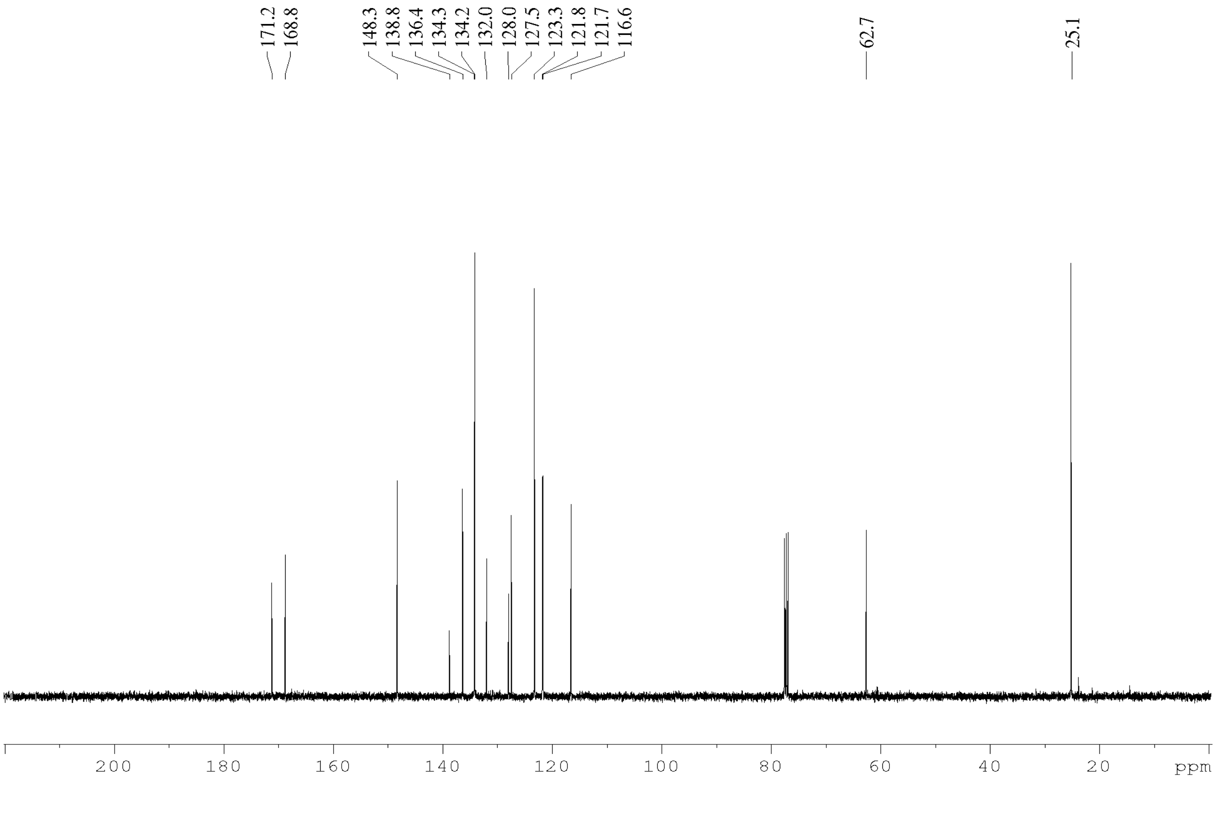

***N*-(quinolin-8-yl)cyclopropanecarboxamide (1k)**

^{1}H NMR ($CDCl_3$, 400 MHz)

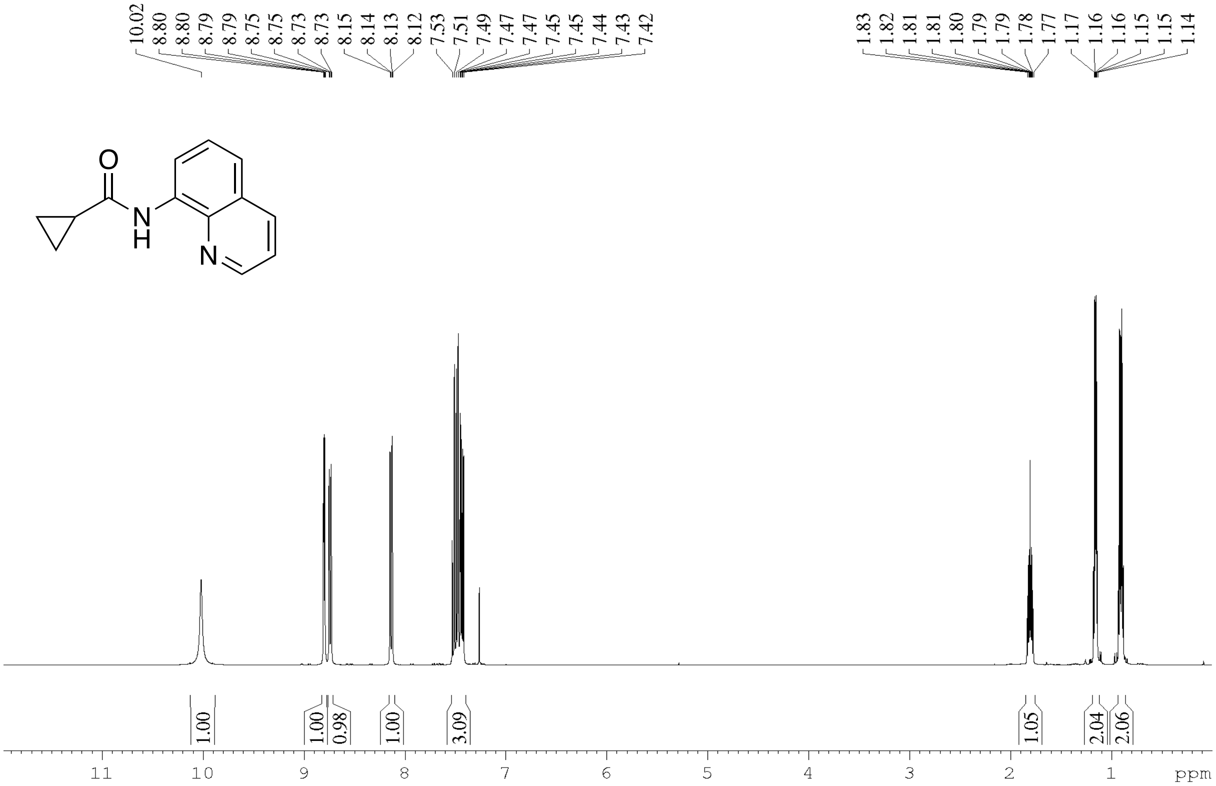

^{13}C NMR ($CDCl_3$, 100 MHz)

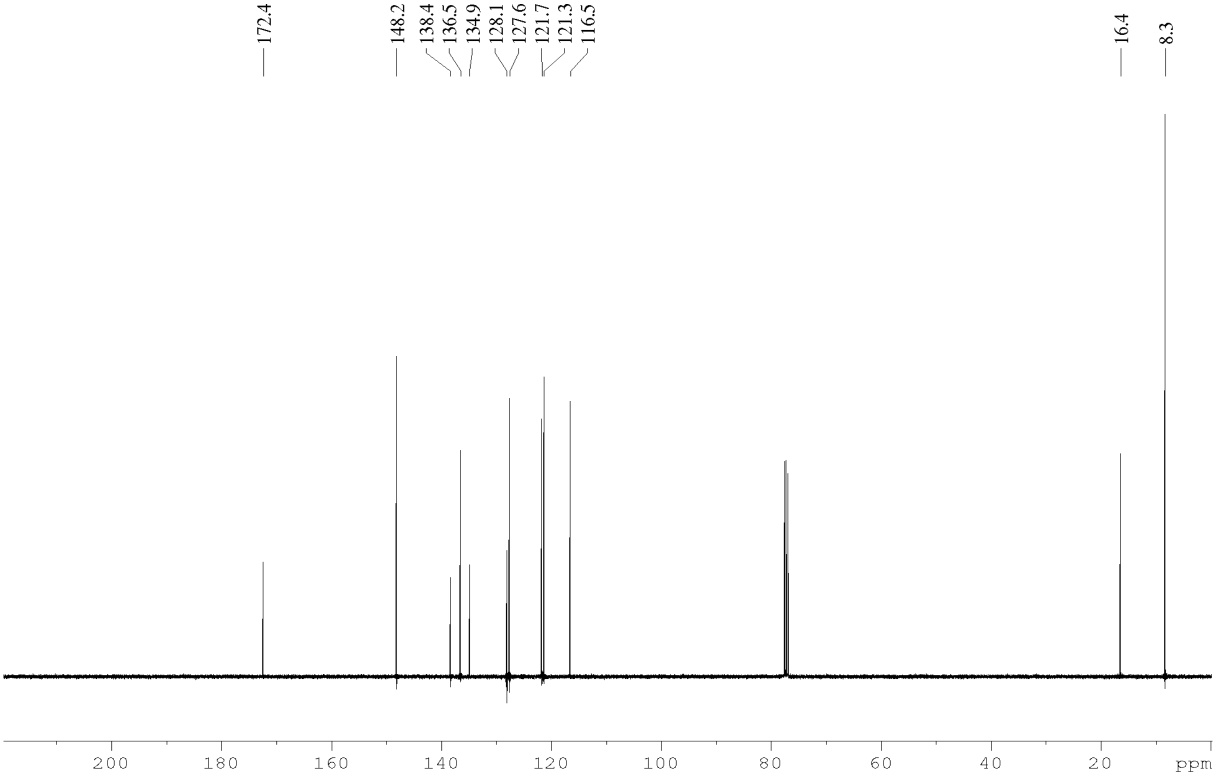

1-methyl-*N*-(quinolin-8-yl)cyclopropane-1-carboxamide (1l)

^{1}H NMR ($CDCl_3$, 400 MHz)

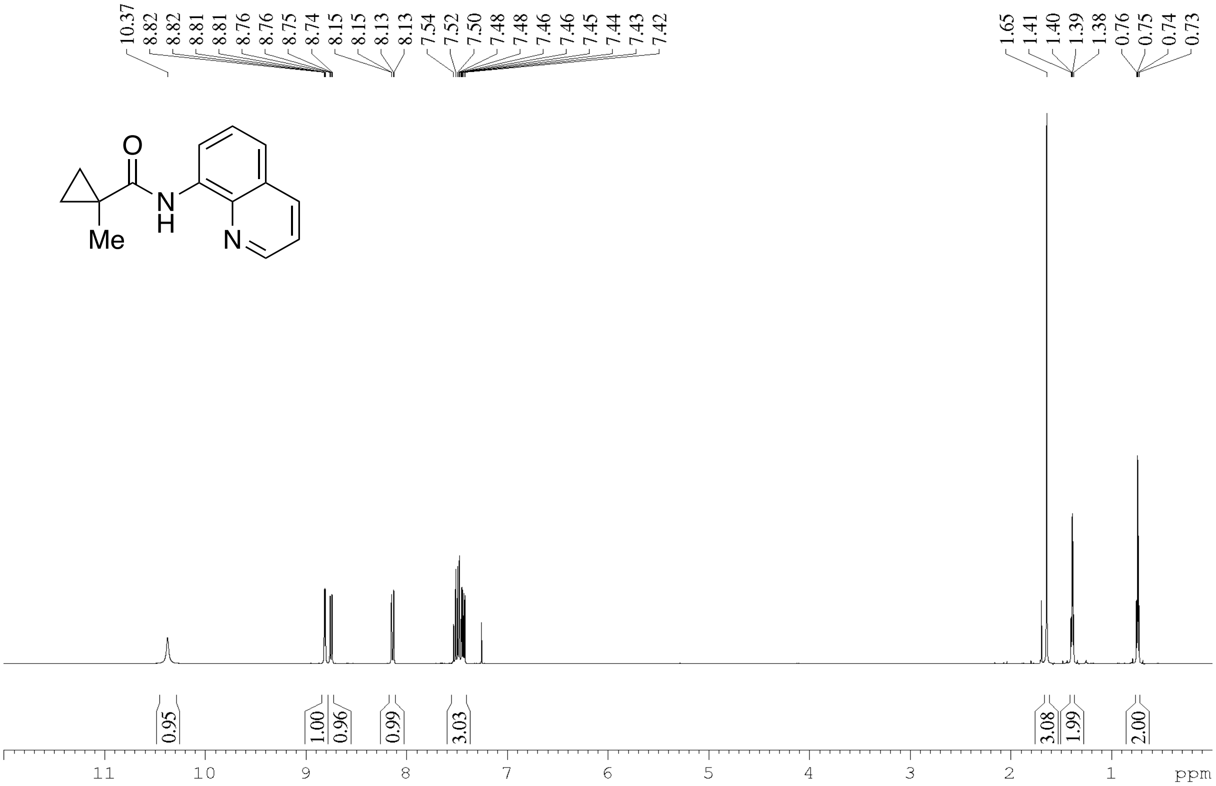

^{13}C NMR ($CDCl_3$, 100 MHz)

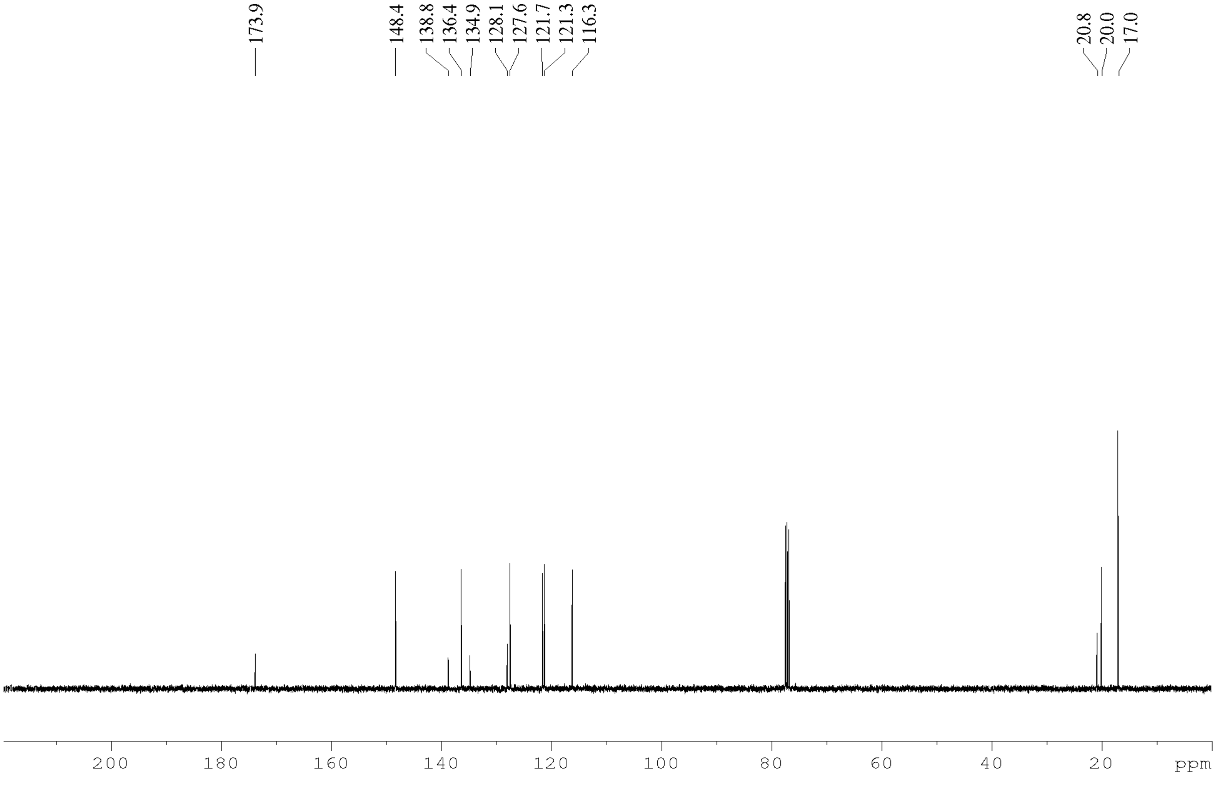

1-(1,3-dioxoisoindolin-2-yl)-*N*-(quinolin-8-yl)cyclopropane-1-carboxamide (1m)

^{1}H NMR (CDCl$_3$, 400 MHz)

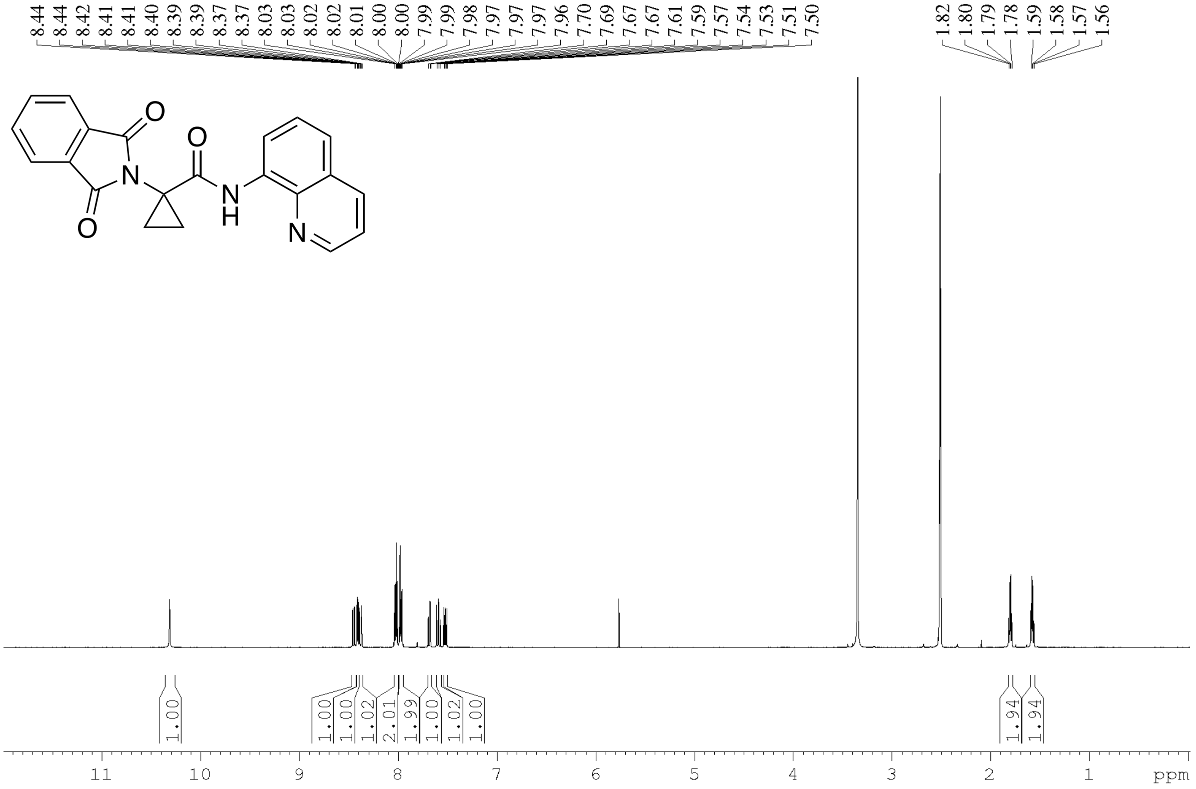

^{13}C NMR (CDCl$_3$, 100 MHz)

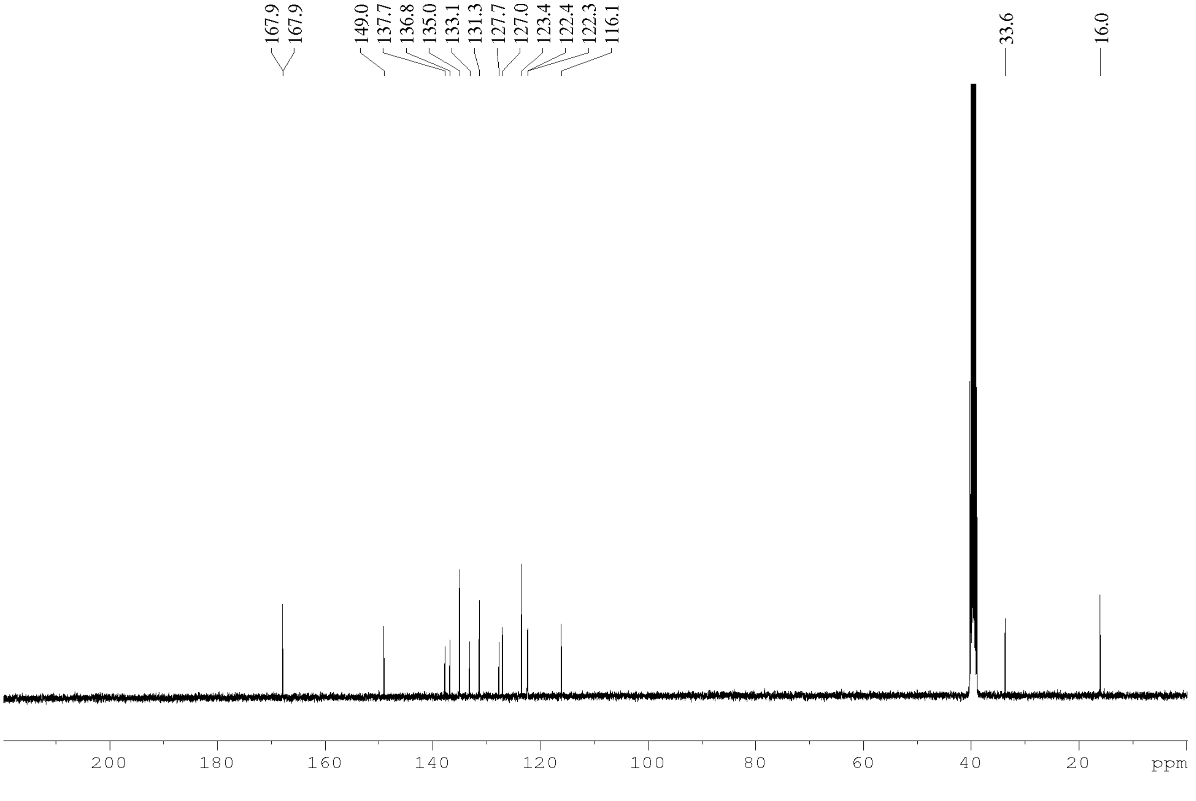

2,2-diphenyl-*N*-(quinolin-8-yl)cyclopropane-1-carboxamide (1n)

^{1}H NMR ($CDCl_3$, 400 MHz)

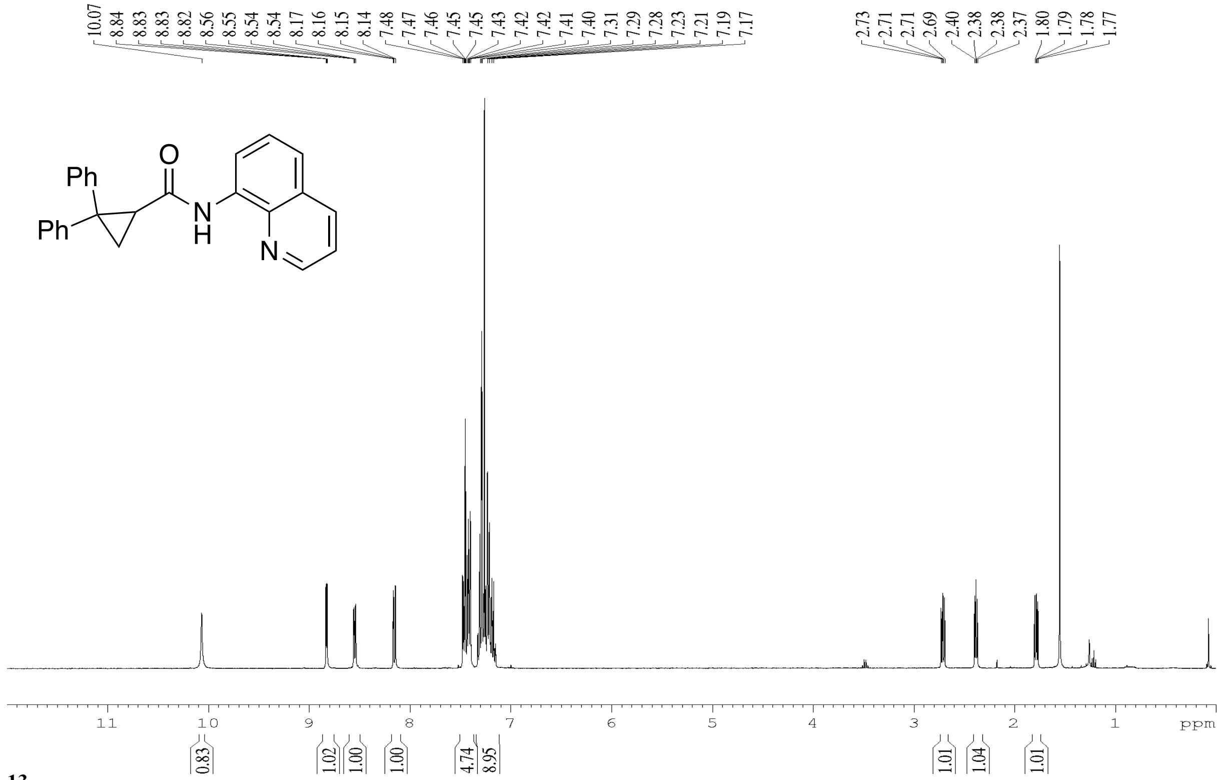

^{13}C NMR ($CDCl_3$, 100 MHz)

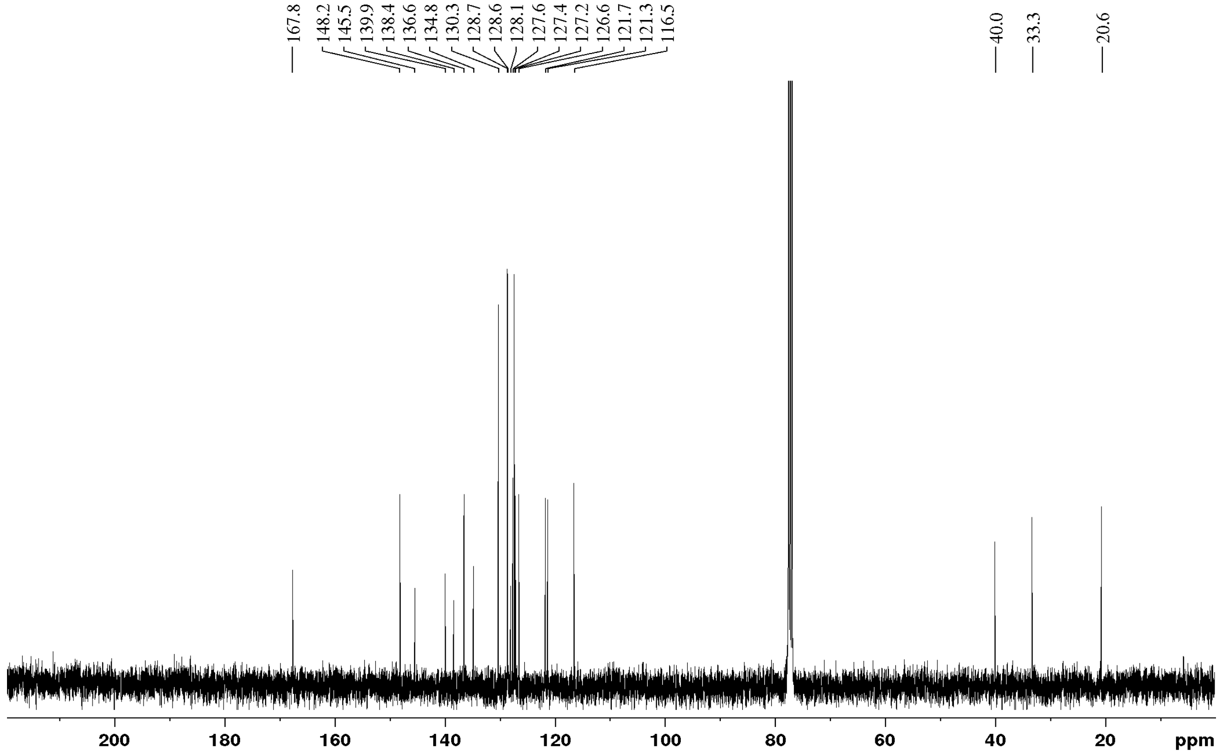

N-(quinolin-8-yl)isobutyramide (1o)

^{1}H NMR ($CDCl_3$, 400 MHz)

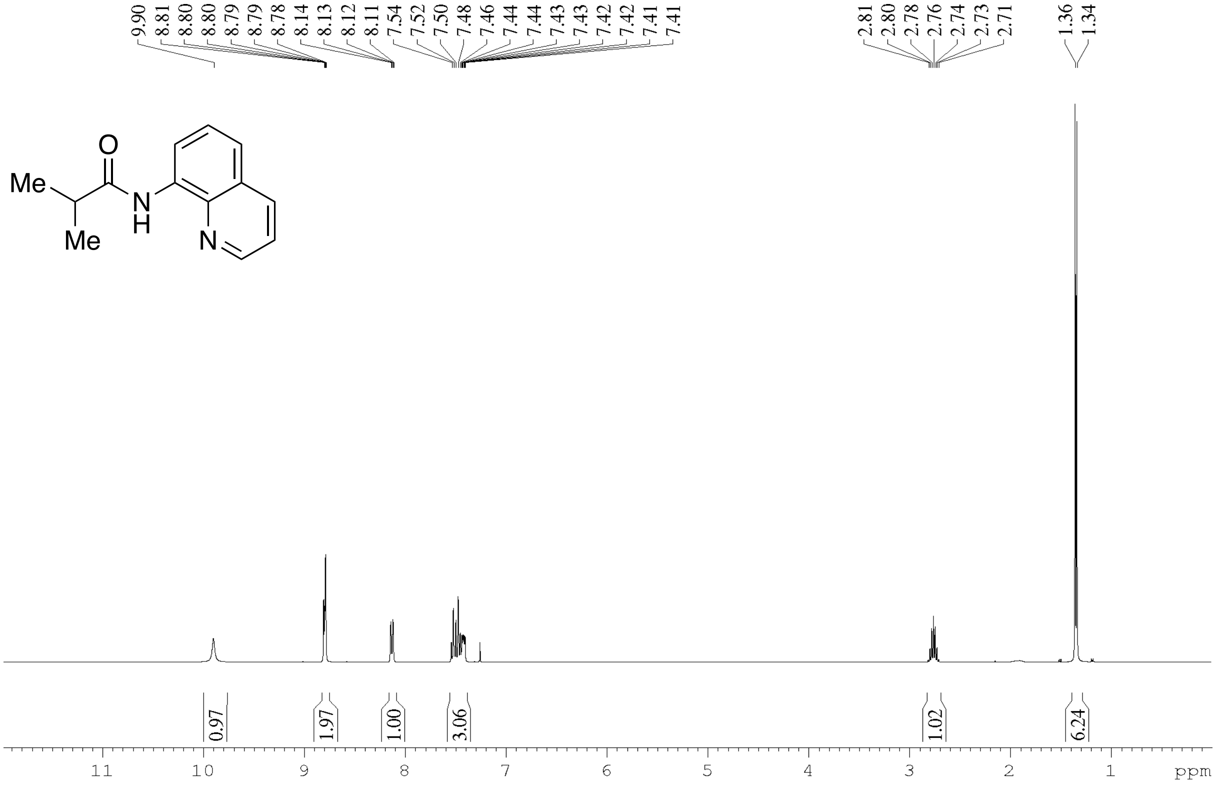

^{13}C NMR ($CDCl_3$, 100 MHz)

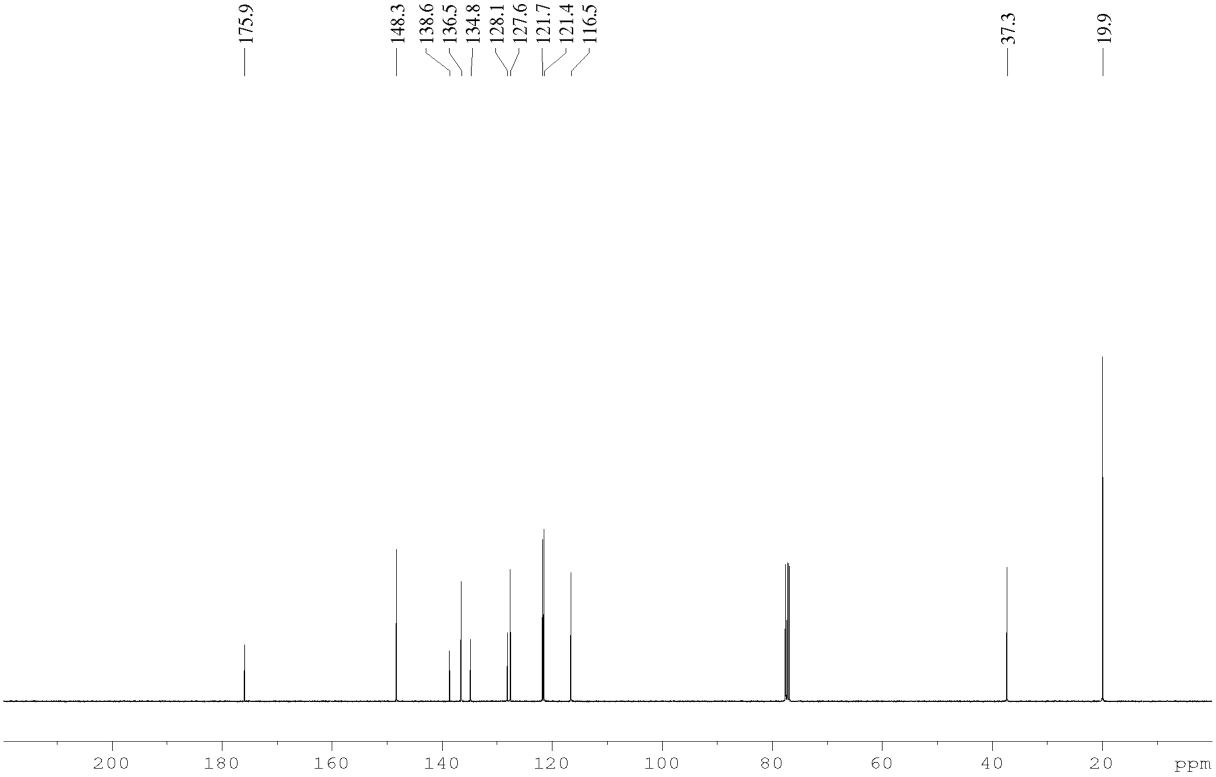

2-(1,3-dioxoisoindolin-2-yl)-*N*-(quinolin-8-yl)propanamide (1p)
^{1}H NMR ($CDCl_3$, 400 MHz)

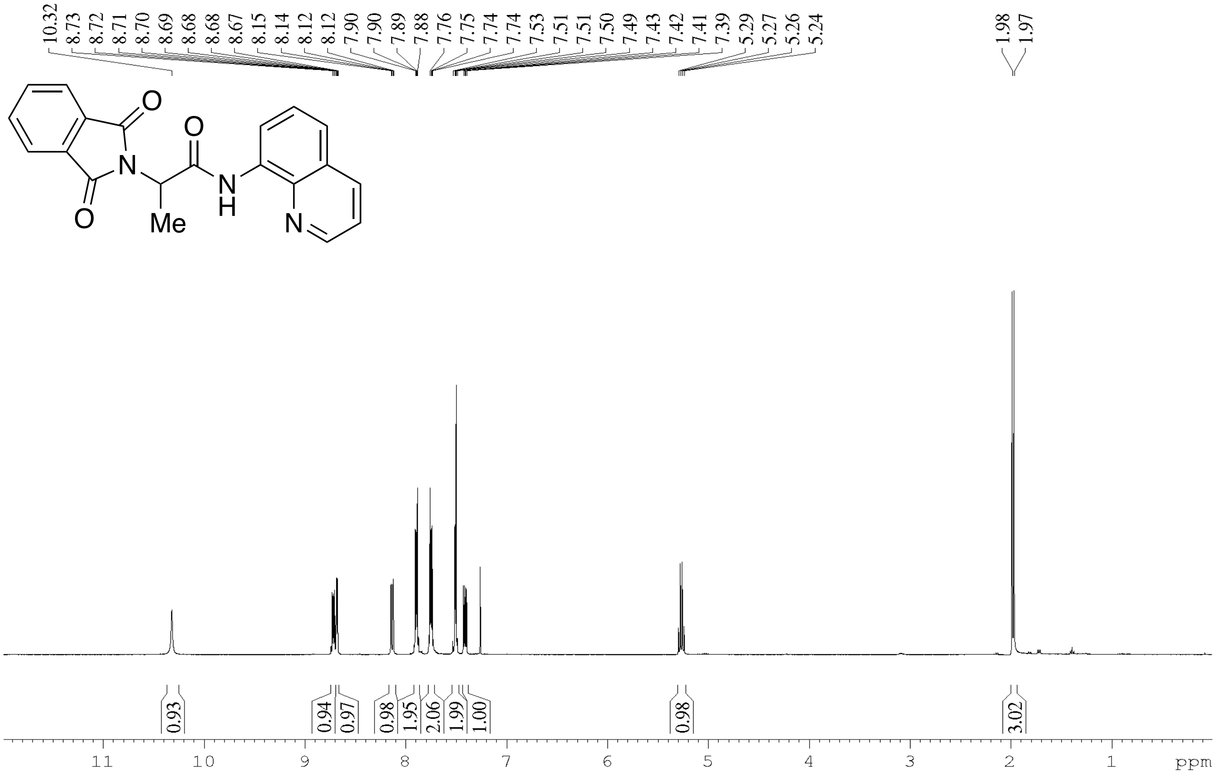

^{13}C NMR ($CDCl_3$, 100 MHz)

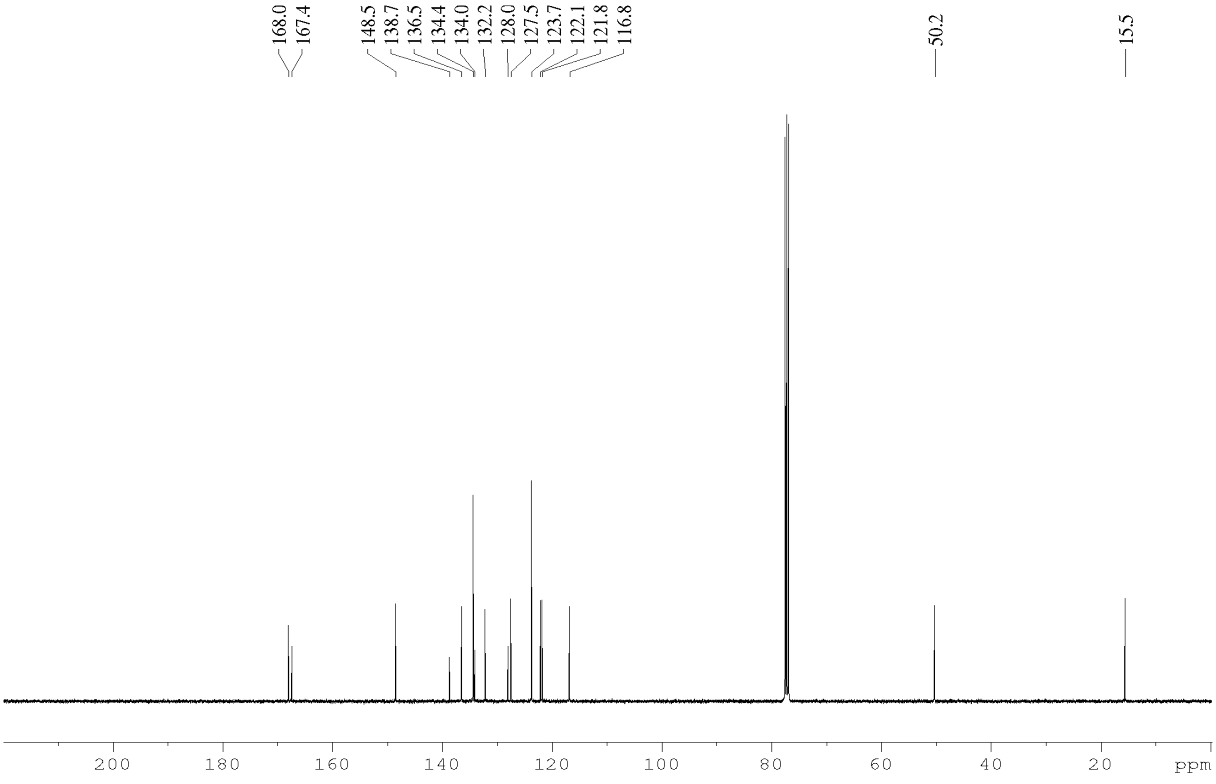

N-(pyridin-2-ylmethyl)pivalamide (**2**)

^{1}H NMR ($CDCl_3$, 400 MHz)

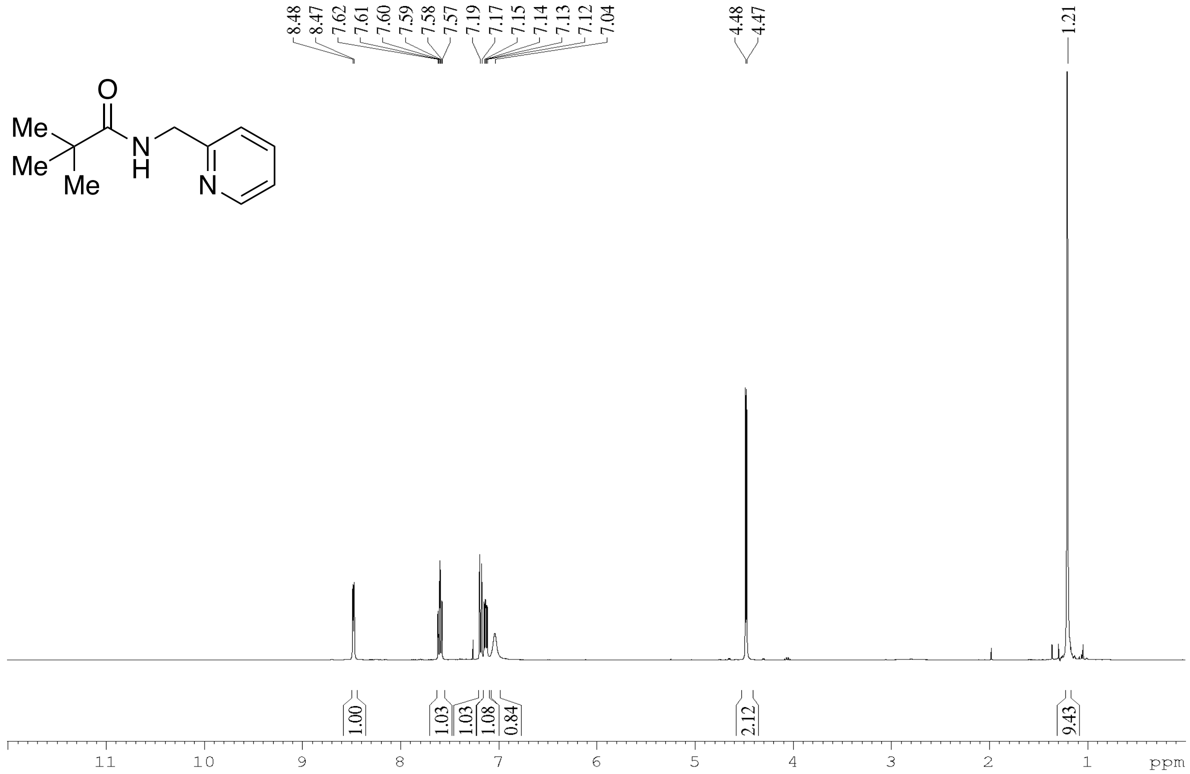

^{13}C NMR ($CDCl_3$, 100 MHz)

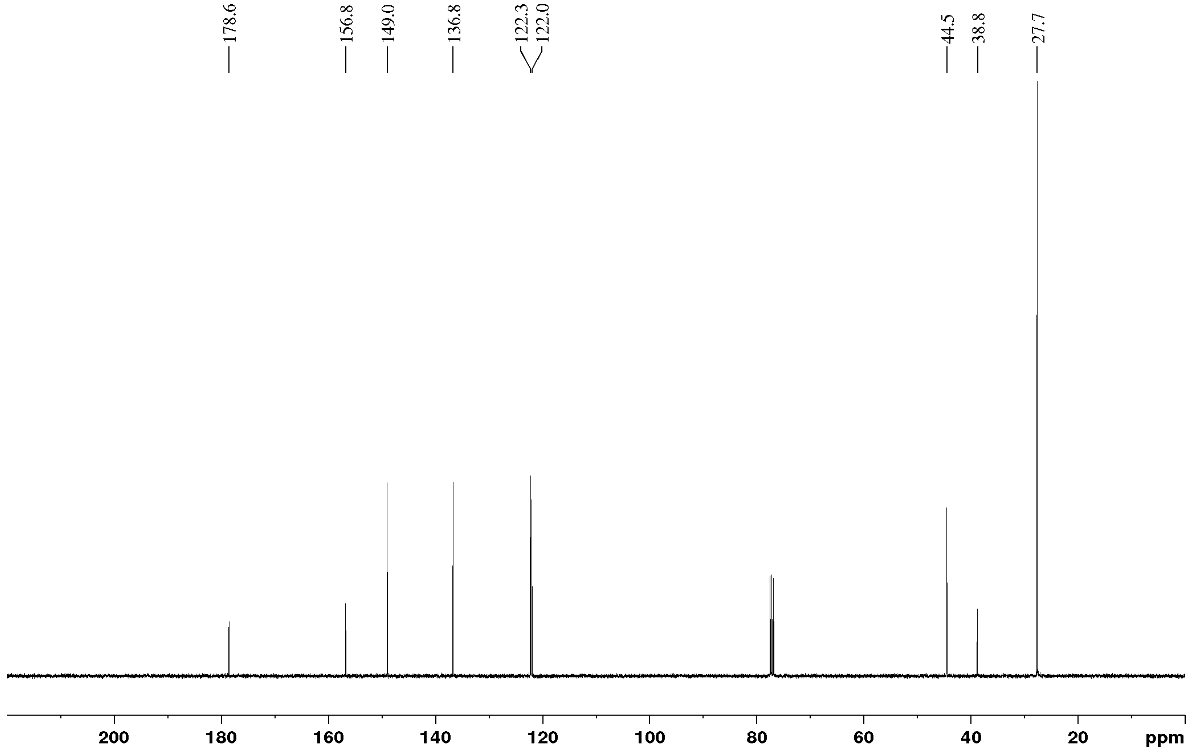

***N*-(2-(pyridin-2-yl)propan-2-yl)pivalamide (3)**

^{1}H NMR ($CDCl_3$, 400 MHz)

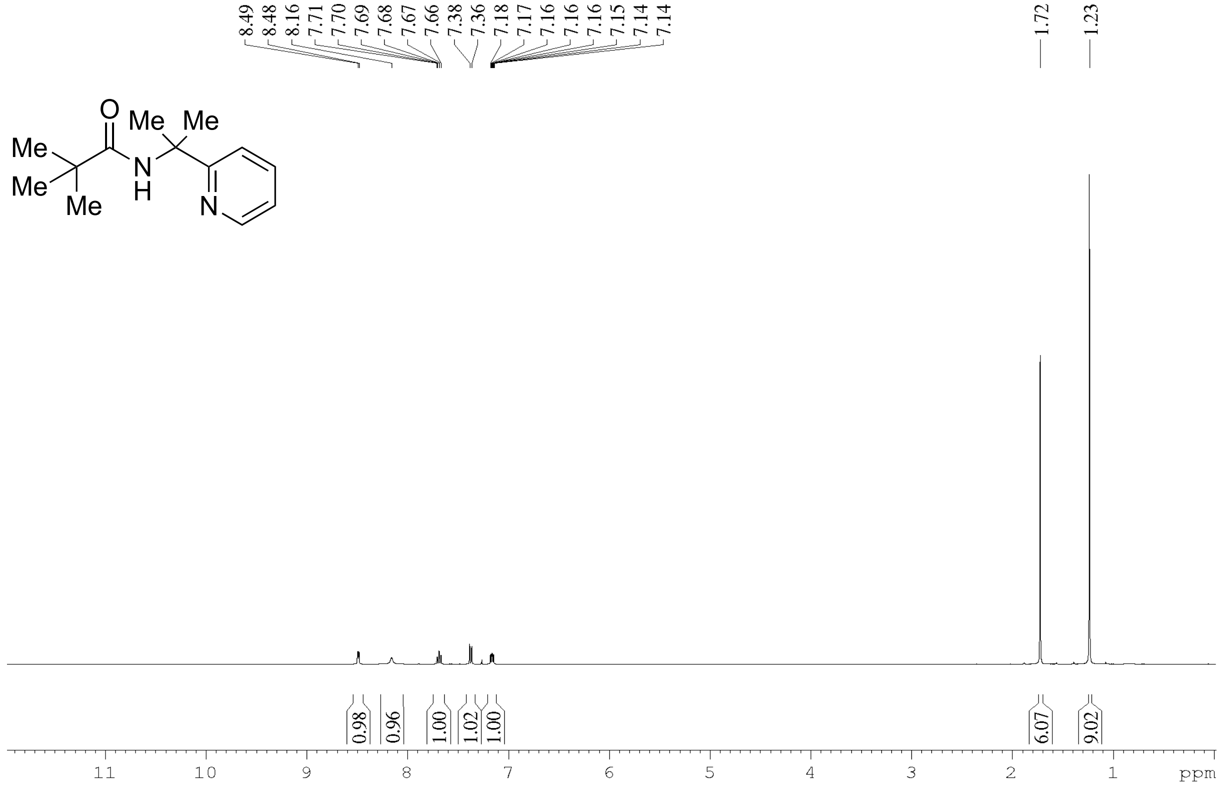

^{13}C NMR ($CDCl_3$, 100 MHz)

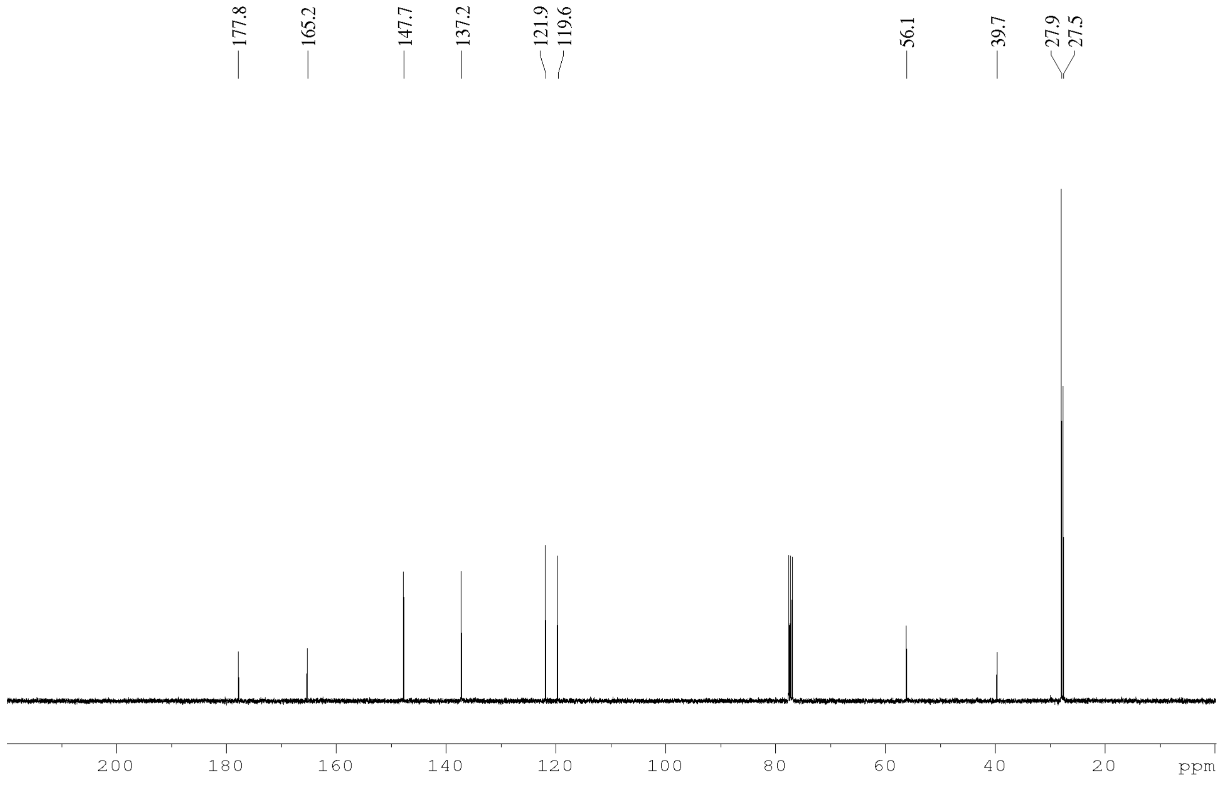

***N*-(pyridin-2-yl)pivalamide (4)**

^{1}H NMR ($CDCl_3$, 500 MHz)

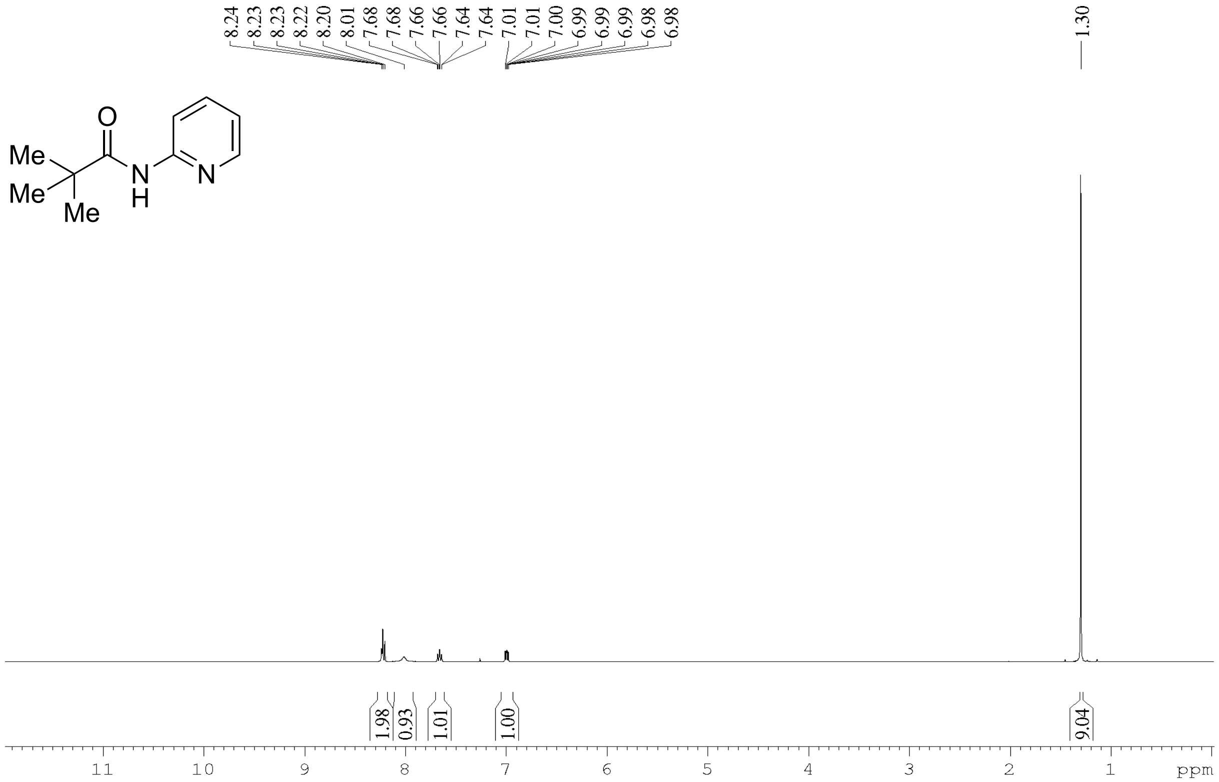

^{13}C NMR ($CDCl_3$, 100 MHz)

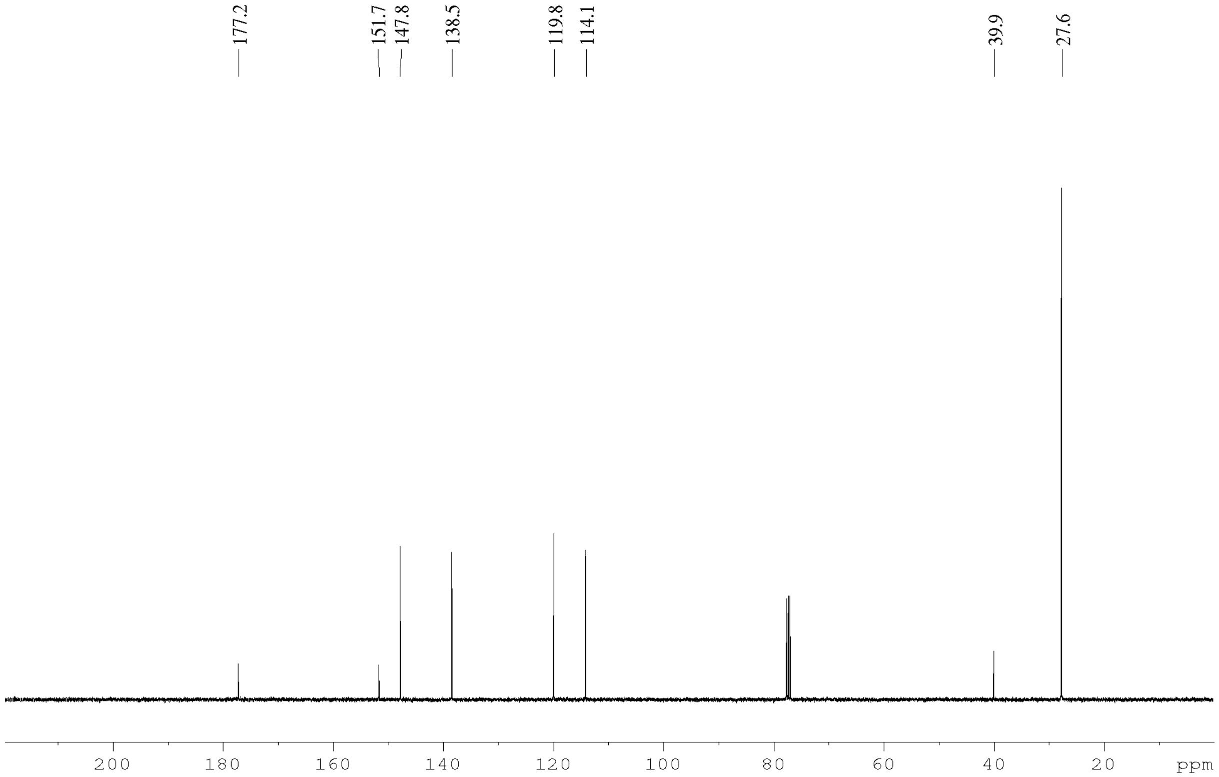

2-pivalamidopyridine 1-oxide (5)

^{1}H NMR ($CDCl_3$, 400 MHz)

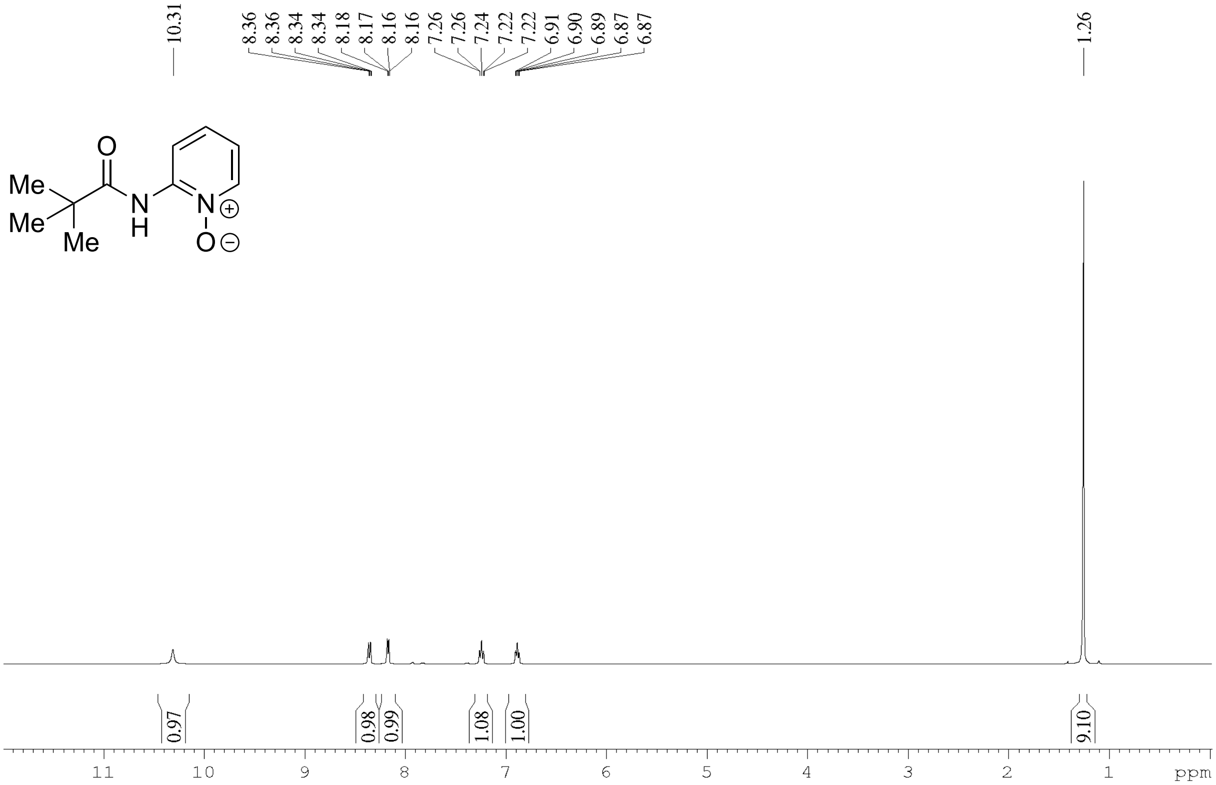

^{13}C NMR ($CDCl_3$, 100 MHz)

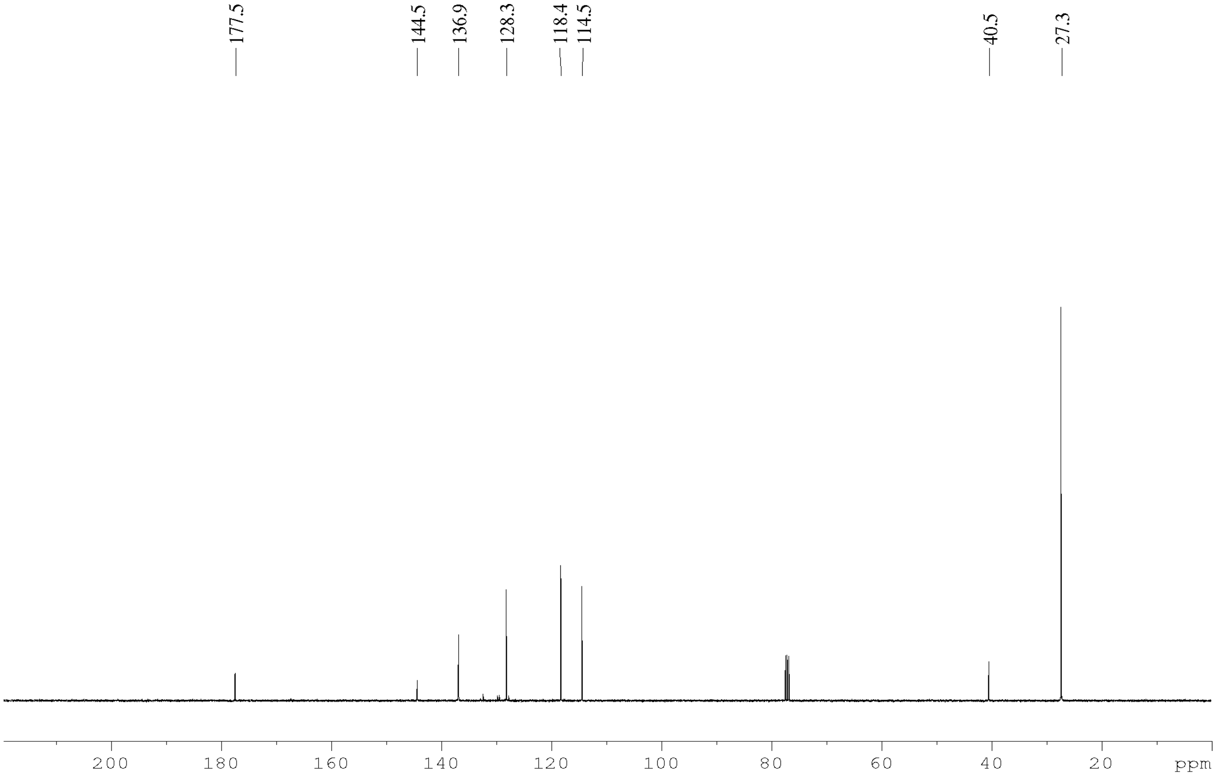

N-methylpivalamide (6)

^{1}H NMR ($CDCl_3$, 400 MHz)

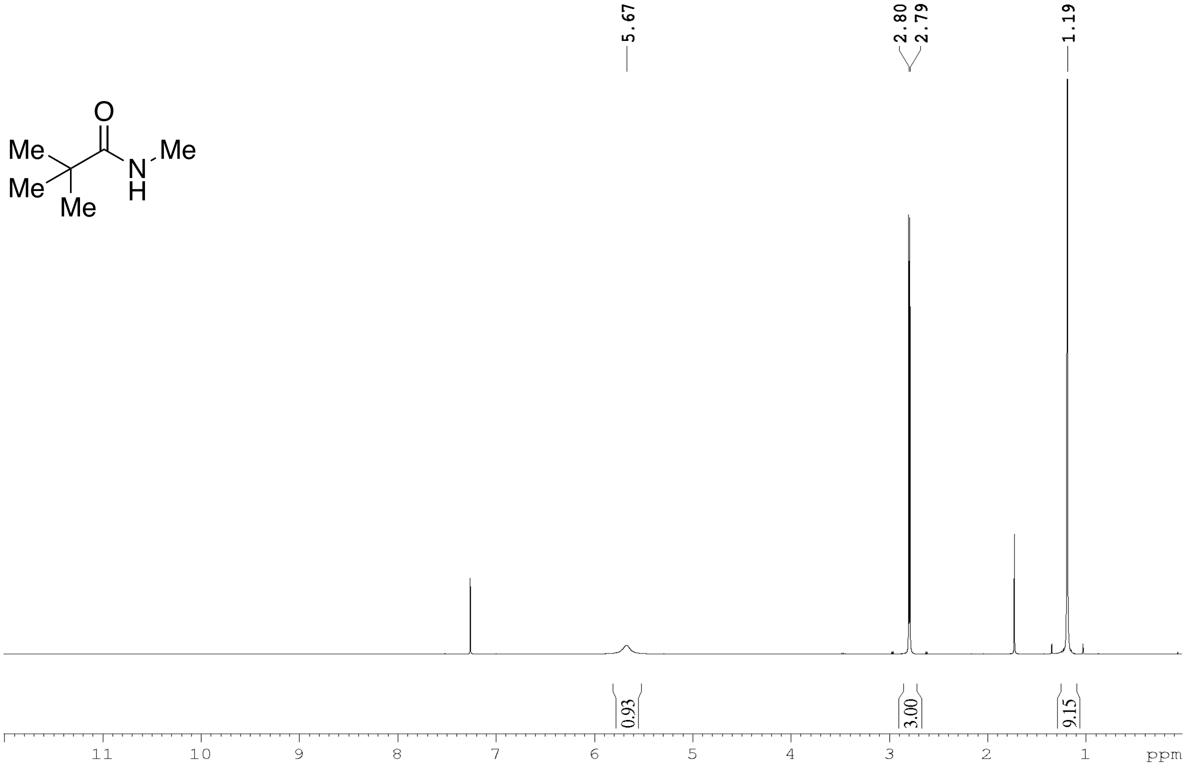

^{13}C NMR ($CDCl_3$, 100 MHz)

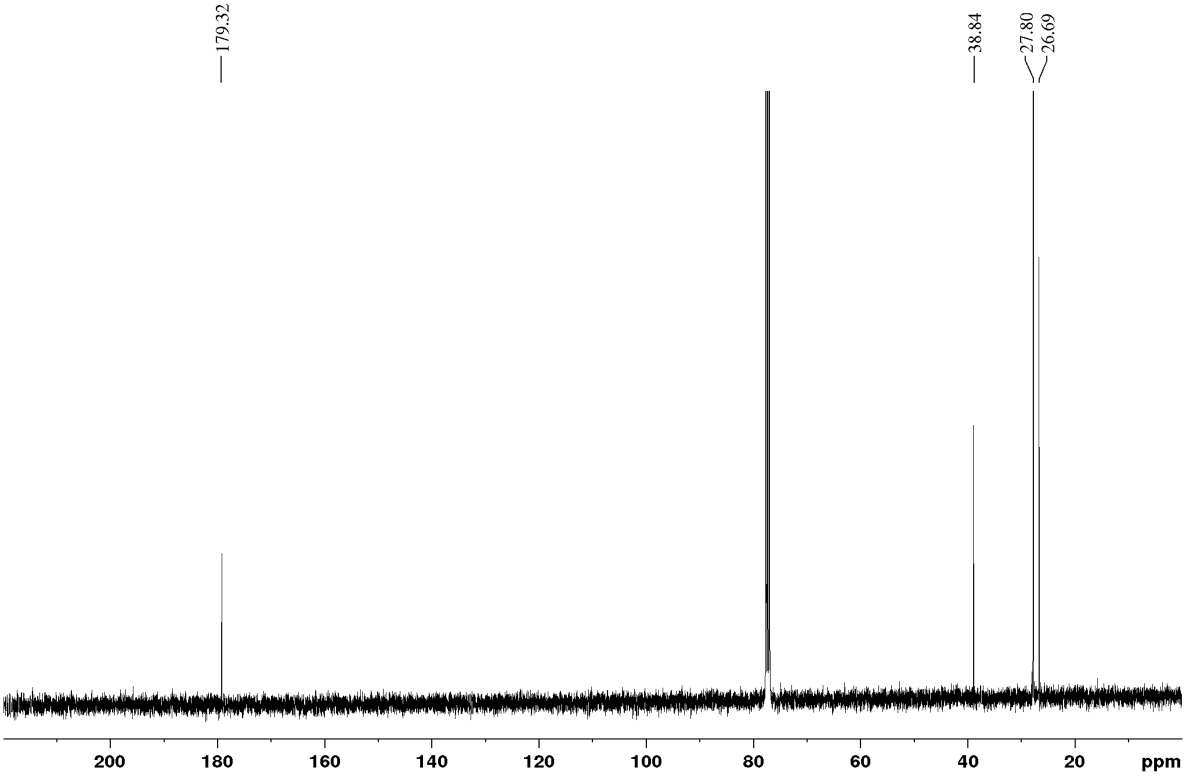

3,3-dimethyl-1-(quinolin-8-yl)pyrrolidine-2,5-dione (7a)

^{1}H NMR (CDCl$_3$, 400 MHz)

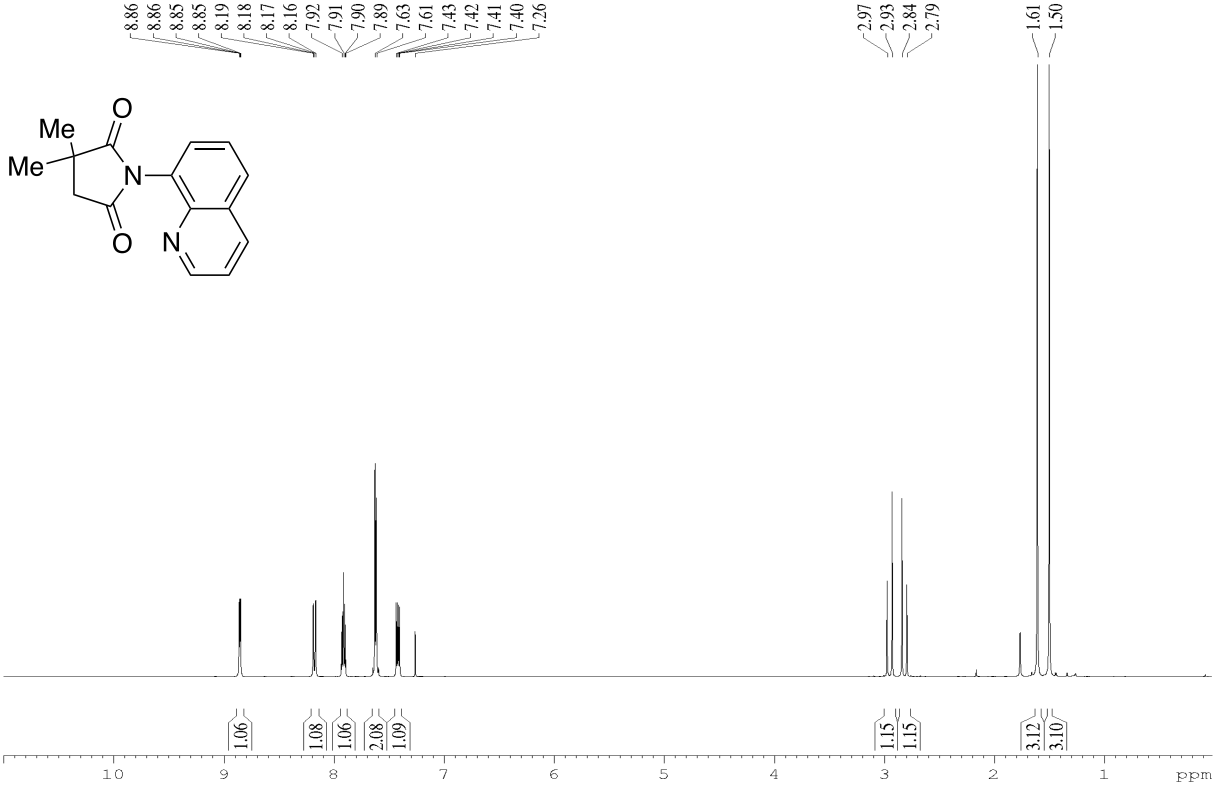

^{13}C NMR (CDCl$_3$, 100 MHz)

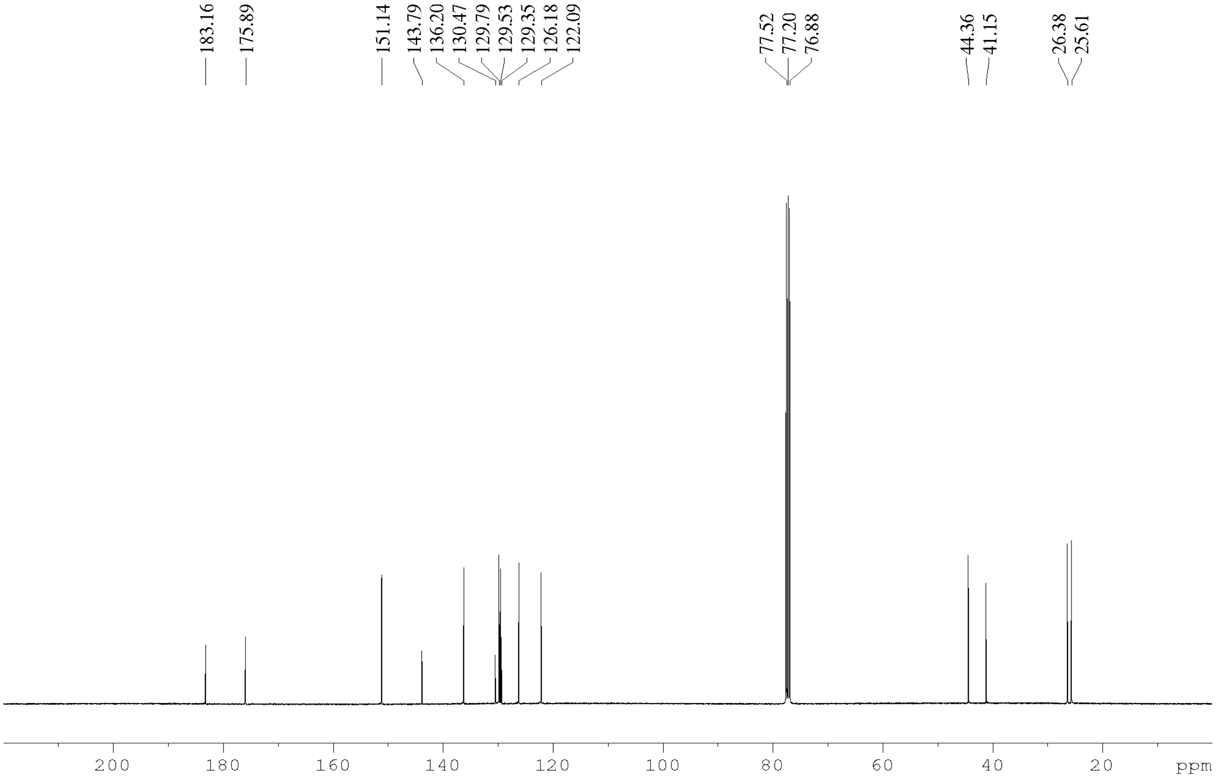

3-ethyl-3-methyl-1-(quinolin-8-yl)pyrrolidine-2,5-dione (7b)

^{1}H NMR ($CDCl_3$, 400 MHz)

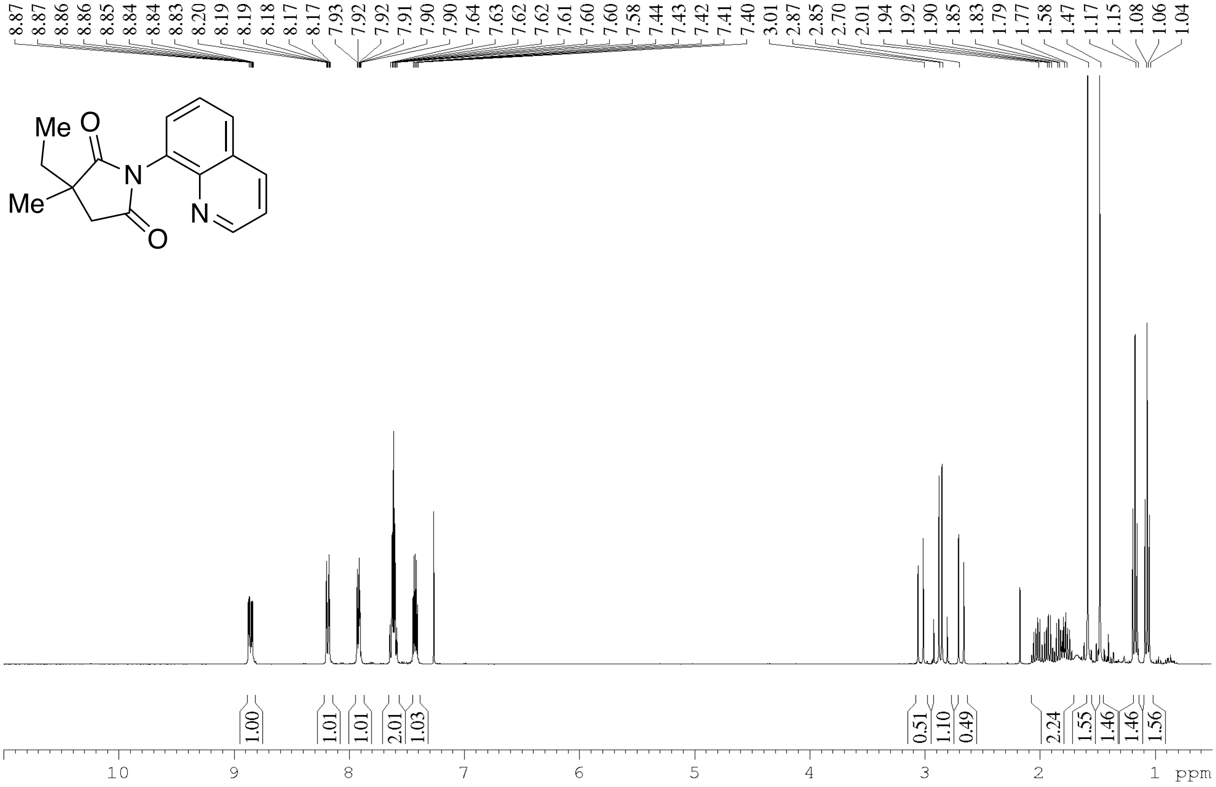

^{13}C NMR ($CDCl_3$, 100 MHz)

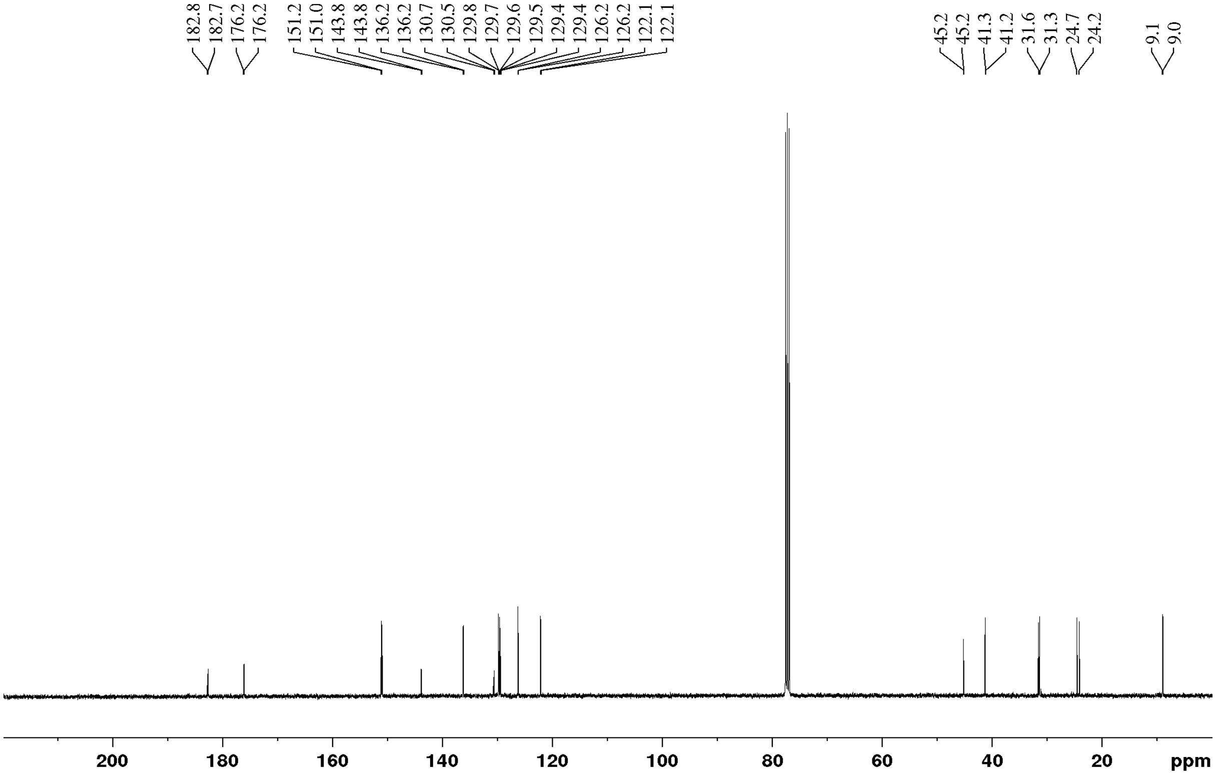

3-methyl-3-phenyl-1-(quinolin-8-yl)pyrrolidine-2,5-dione (7c)

^{1}H NMR ($CDCl_3$, 400 MHz)

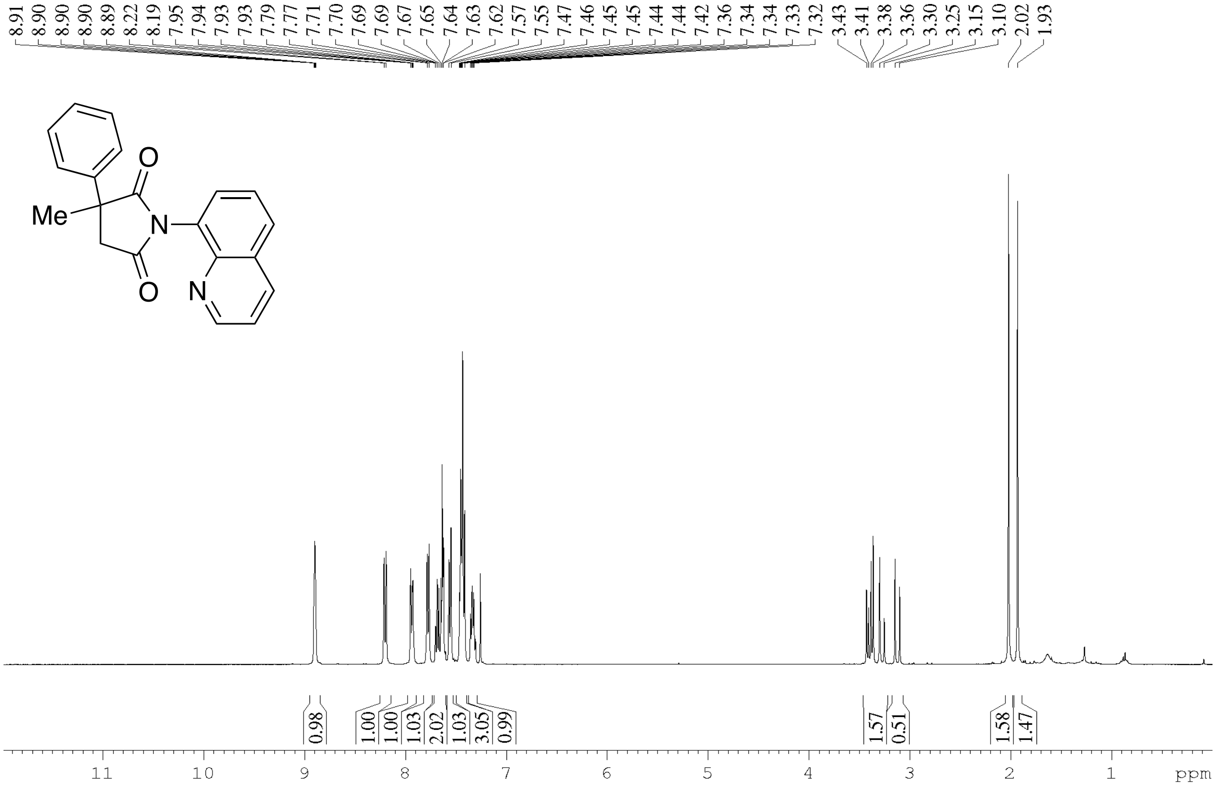

^{13}C NMR ($CDCl_3$, 100 MHz)

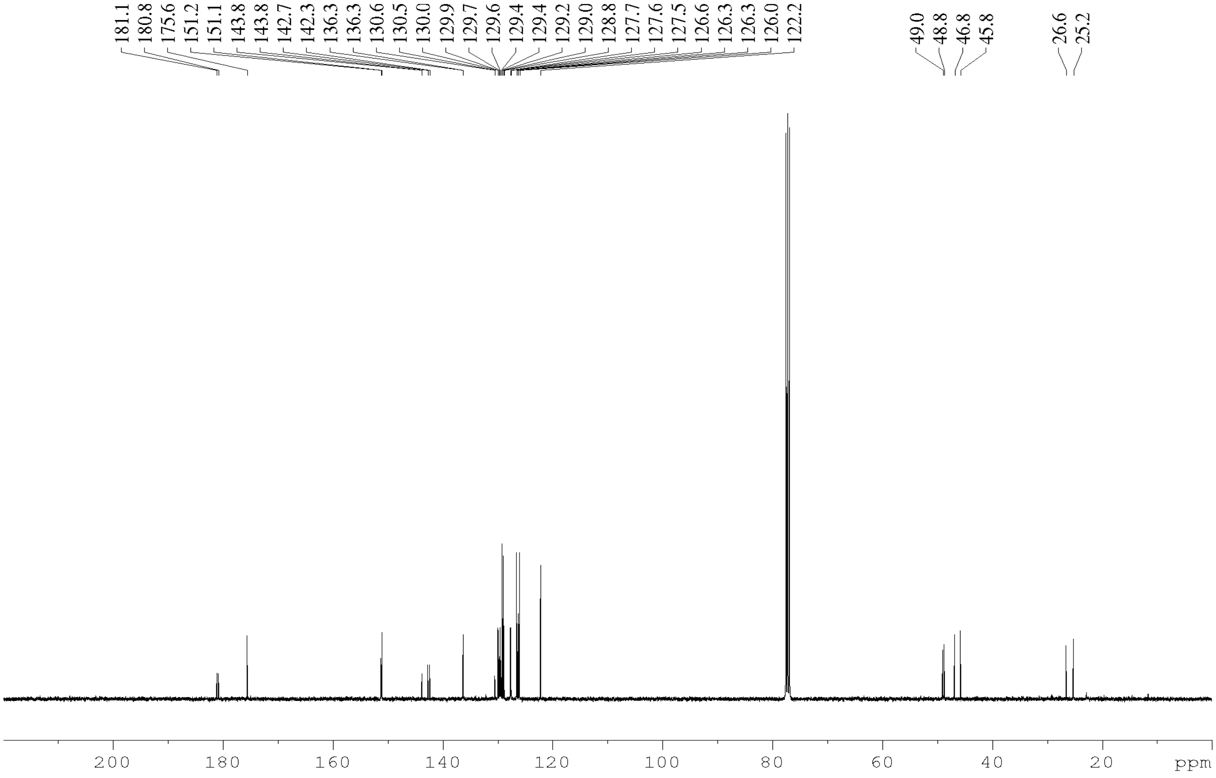

3-benzyl-3-methyl-1-(quinolin-8-yl)pyrrolidine-2,5-dione (7d)

^{1}H NMR ($CDCl_3$, 400 MHz)

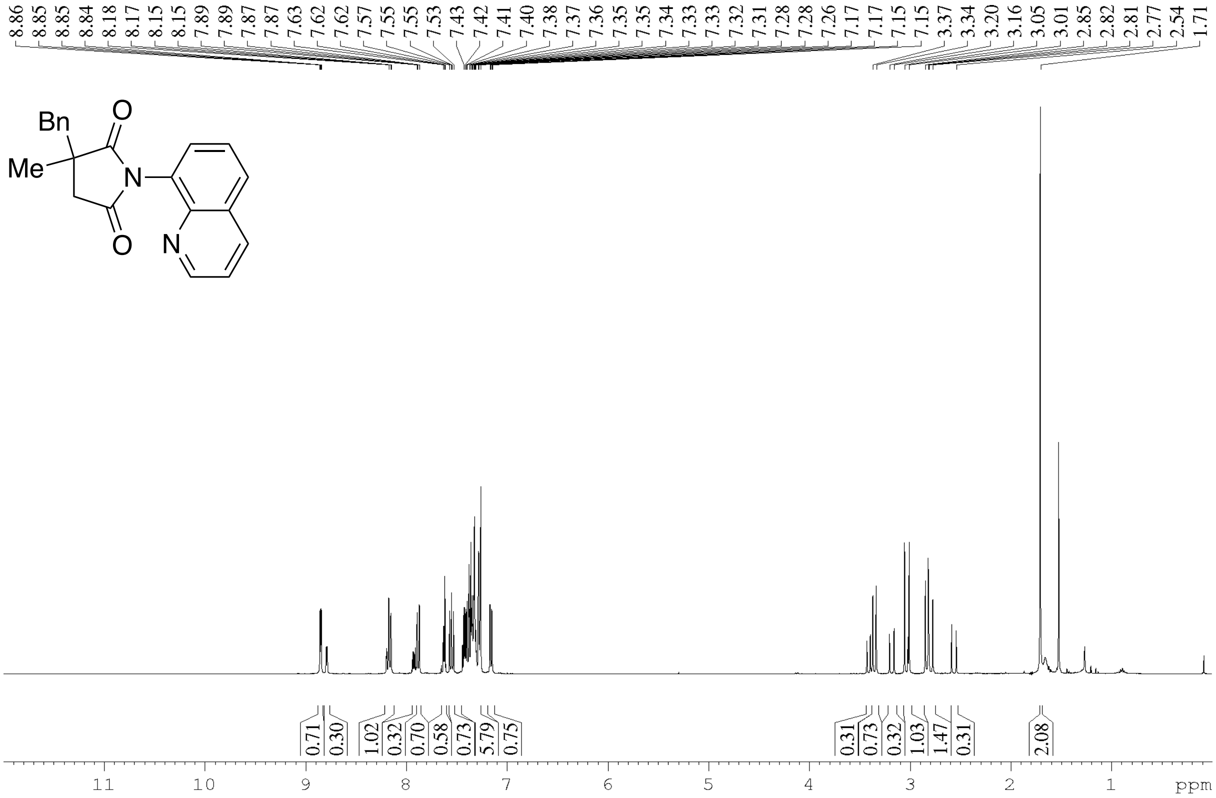

^{13}C NMR ($CDCl_3$, 100 MHz)

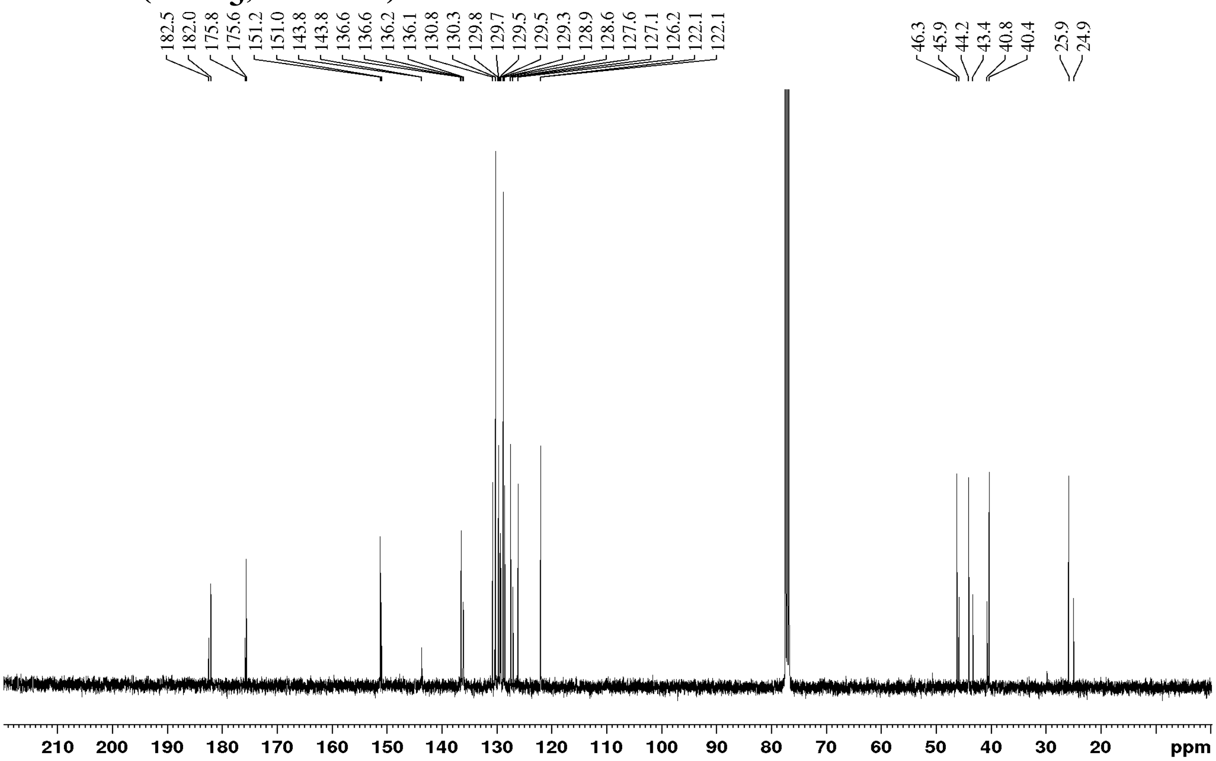

2-(quinolin-8-yl)-2-azaspiro[4.5]decane-1,3-dione (7e)

^{1}H NMR (CDCl$_3$, 400 MHz)

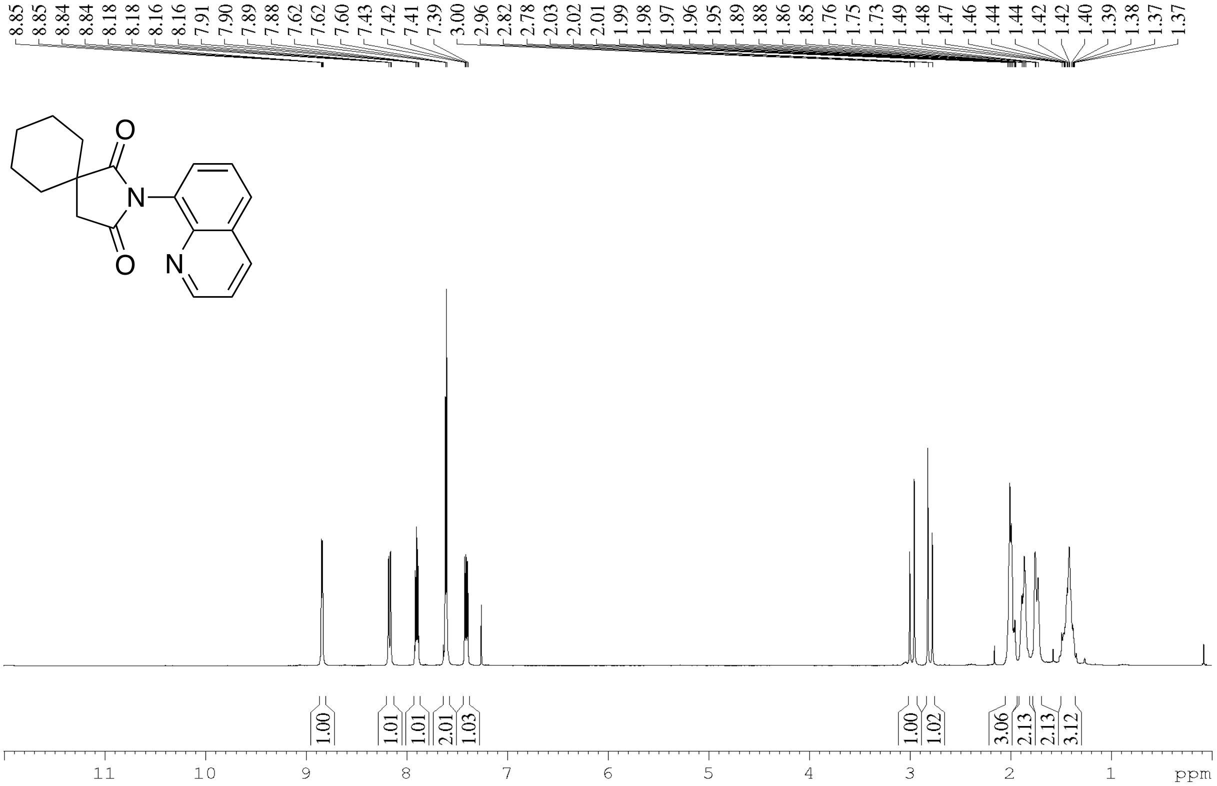

^{13}C NMR (CDCl$_3$, 100 MHz)

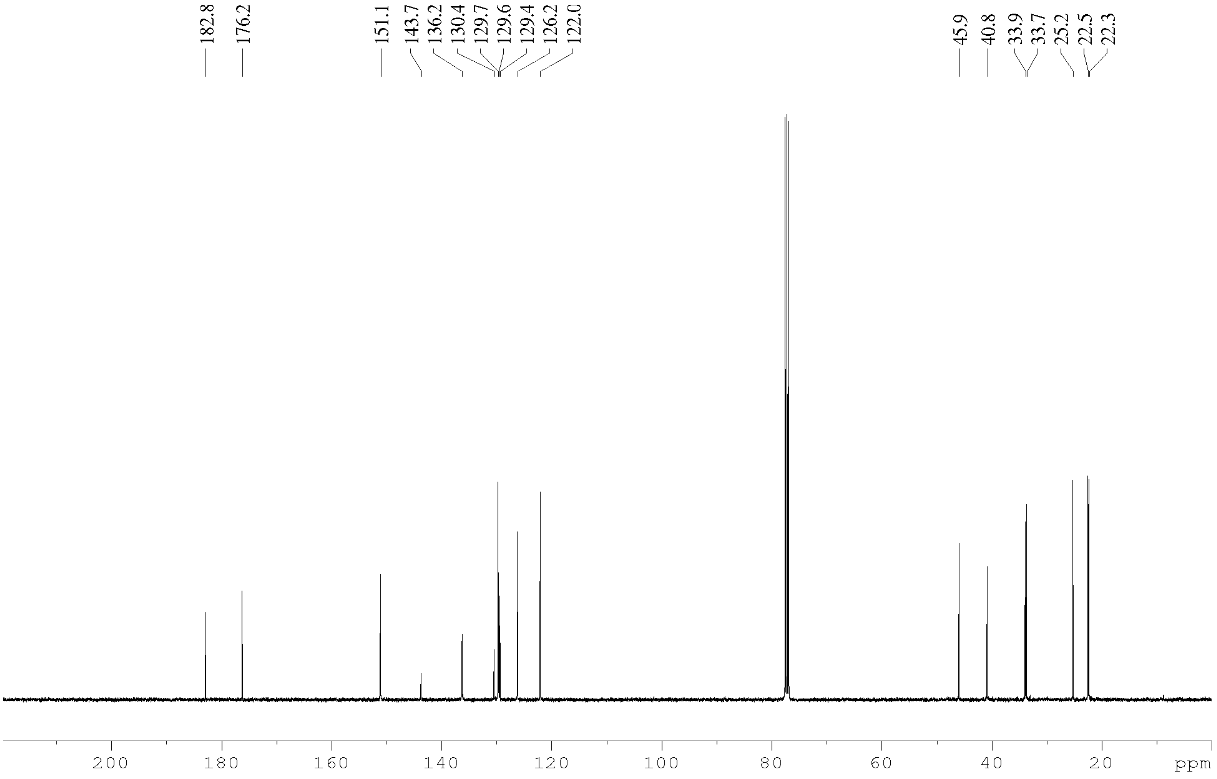

2-(quinolin-8-yl)-8-tosyl-2,8-diazaspiro[4.5]decane-1,3-dione (7f)

^{1}H NMR (CDCl$_3$, 400 MHz)

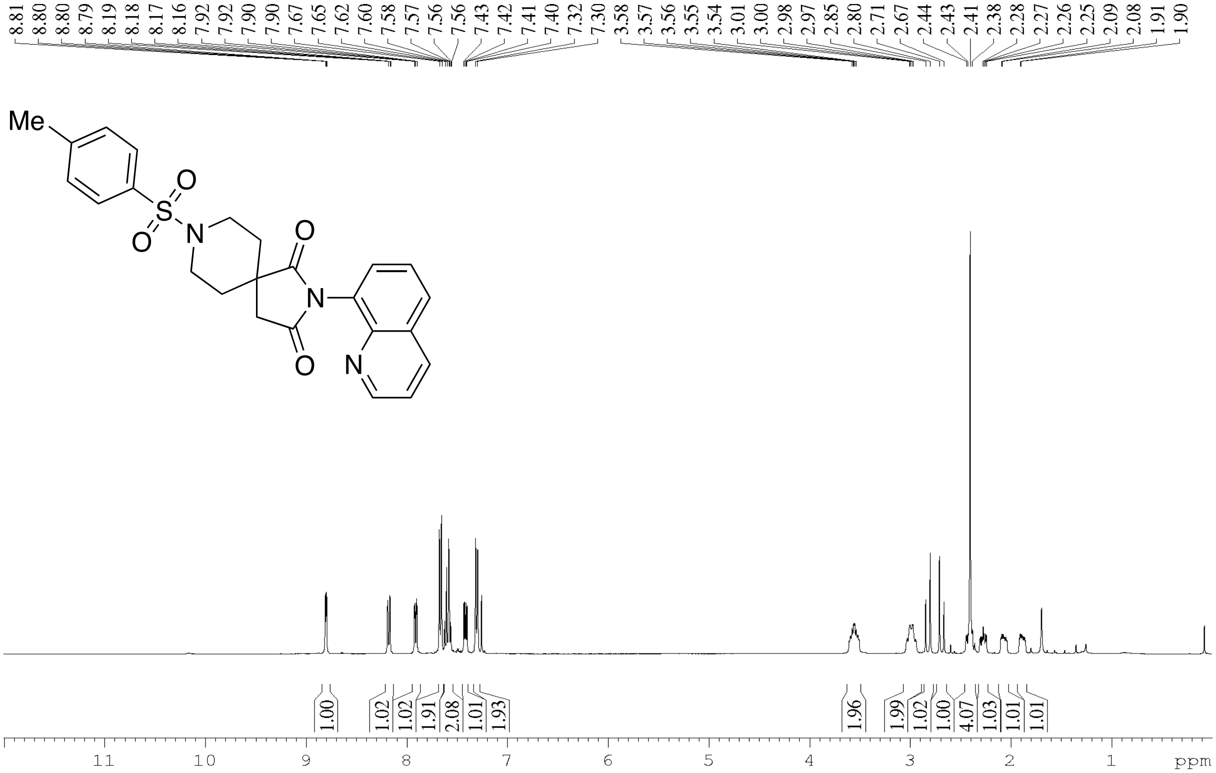

^{13}C NMR (CDCl$_3$, 100 MHz)

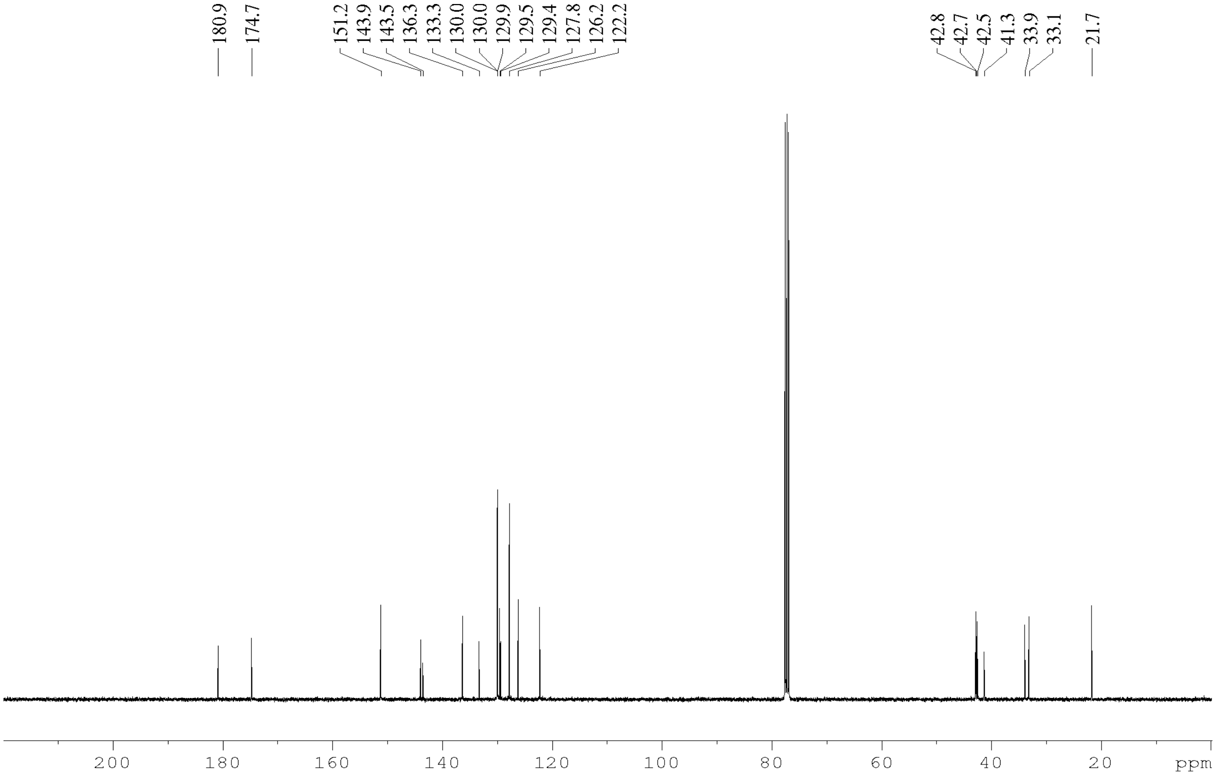

Ethyl 3-methyl-2,5-dioxo-1-(quinolin-8-yl)pyrrolidine-3-carboxylate (7g)

^{1}H NMR ($CDCl_3$, 400 MHz)

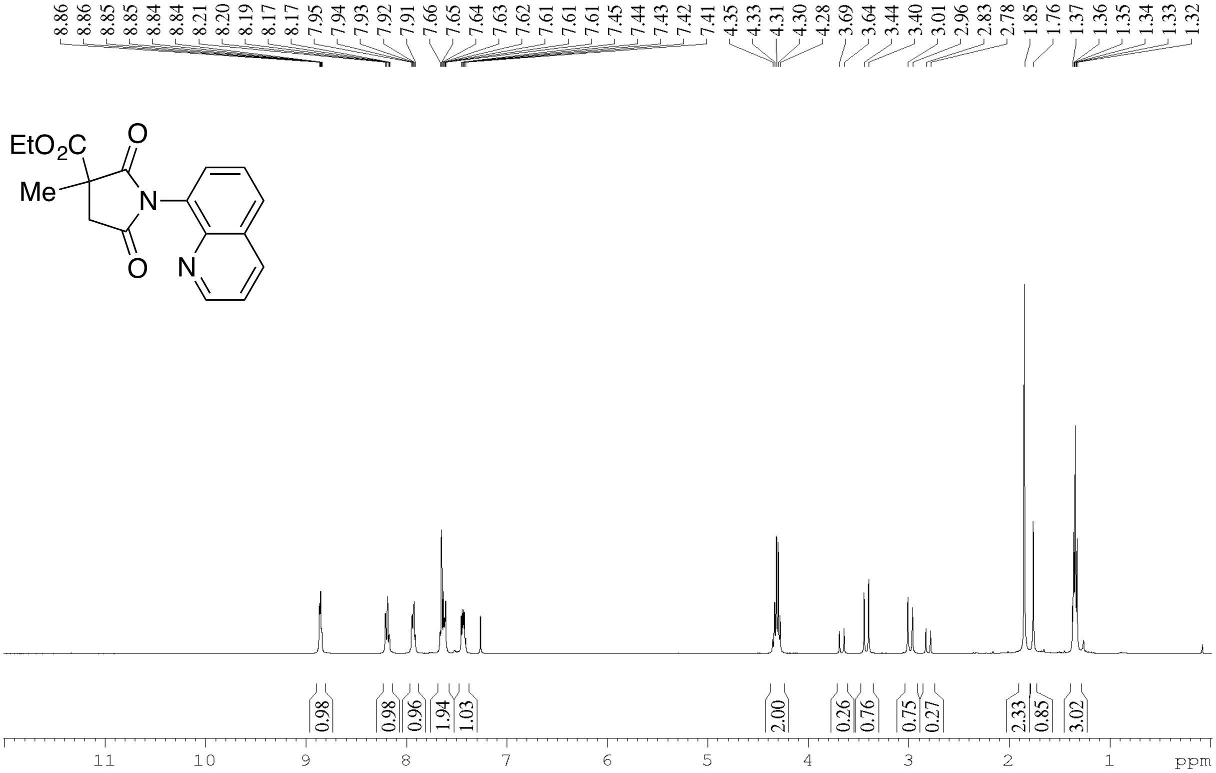

^{13}C NMR ($CDCl_3$, 100 MHz)

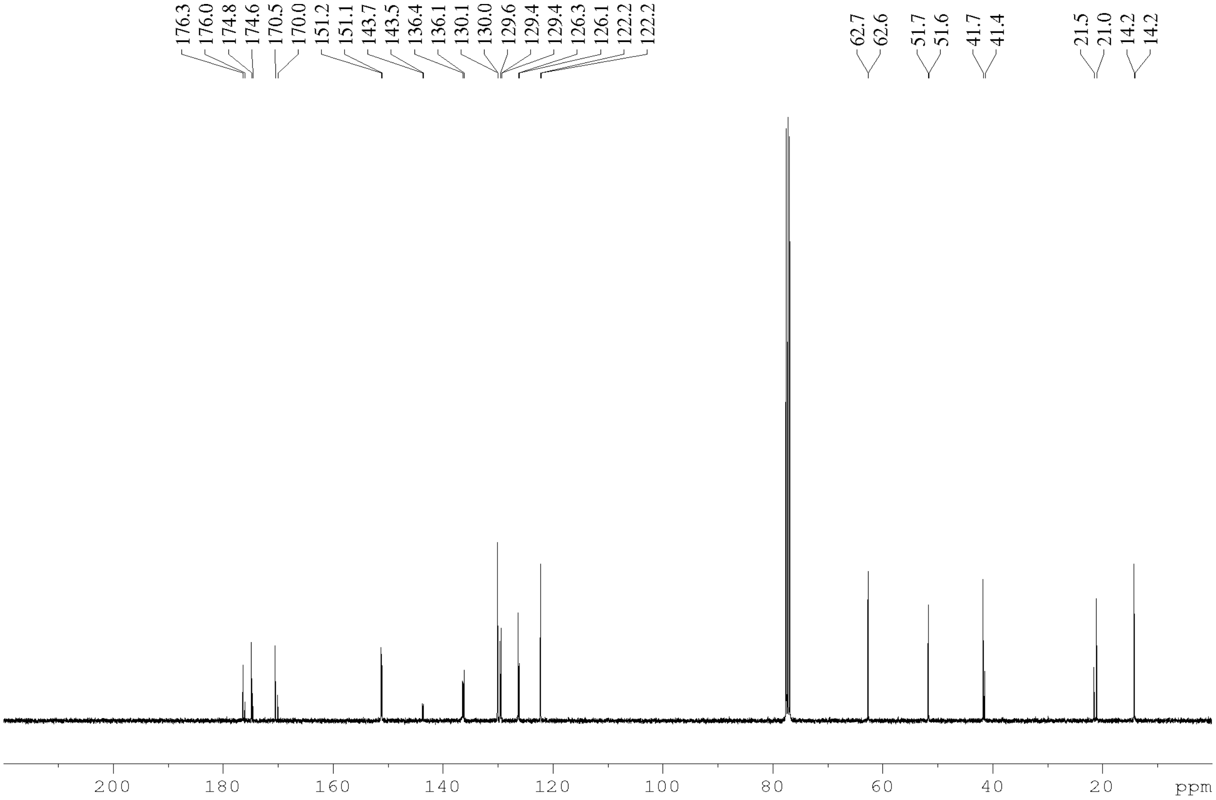

(3-methyl-2,5-dioxo-1-(quinolin-8-yl)pyrrolidin-3-yl)methyl acetate (7h)

^{1}H NMR ($CDCl_3$, 400 MHz)

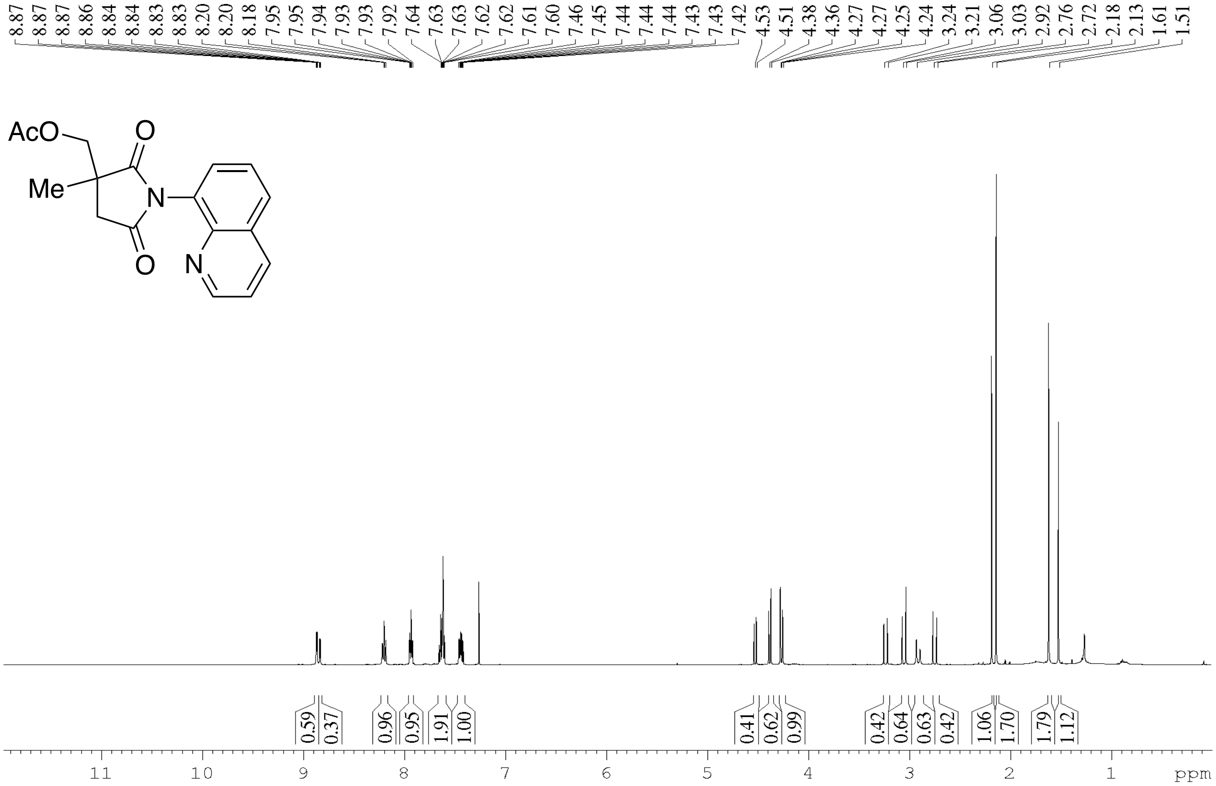

^{13}C NMR ($CDCl_3$, 100MHz)

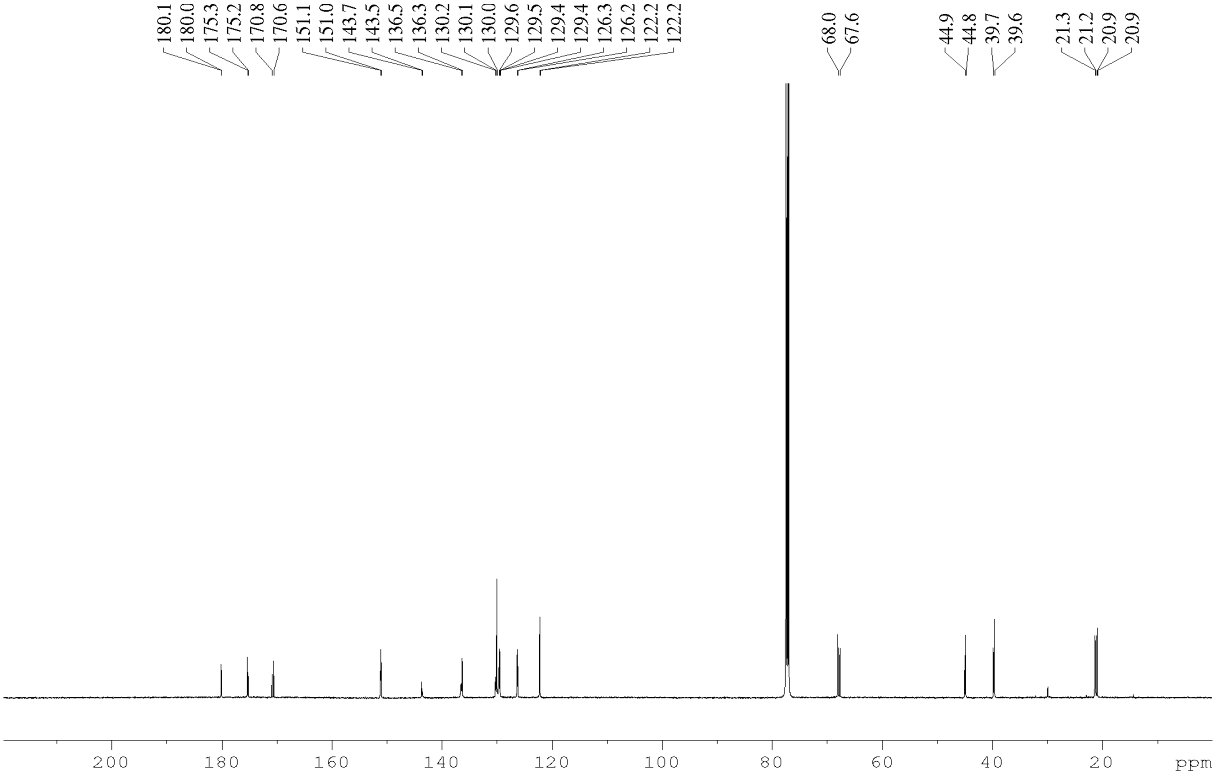

3-methyl-1-(quinolin-8-yl)-3-(trifluoromethyl)pyrrolidine-2,5-dione (7i)

^{1}H NMR ($CDCl_3$, 400 MHz)

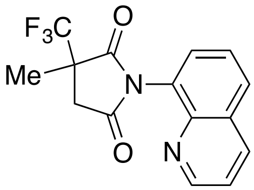

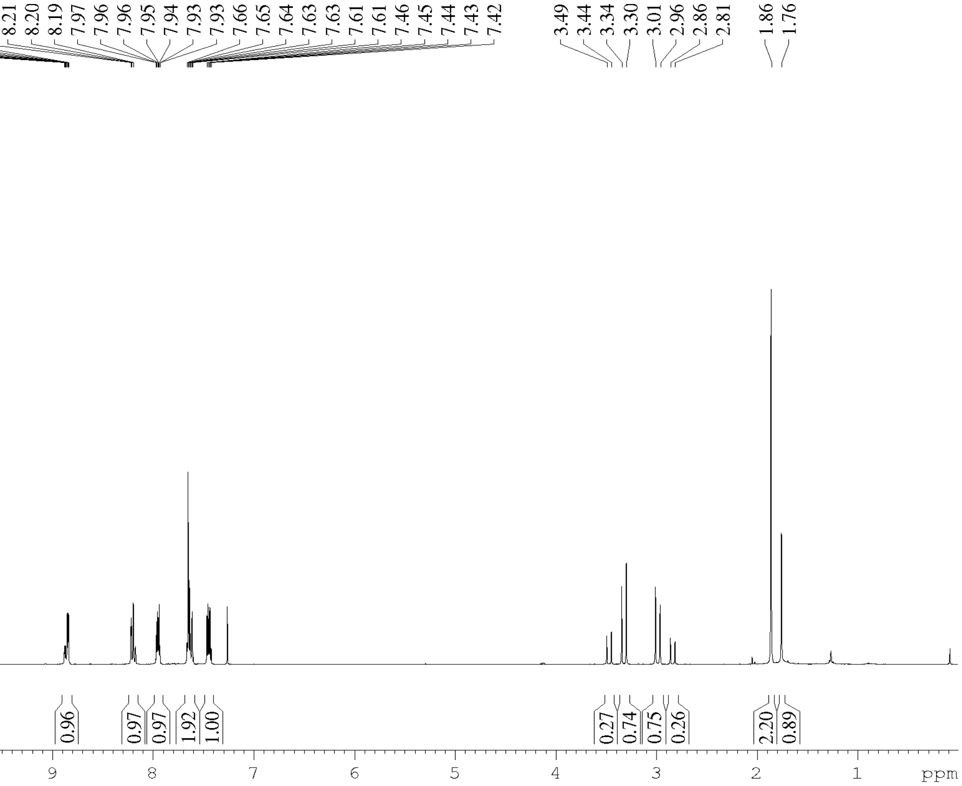

^{13}C NMR ($CDCl_3$, 100 MHz)

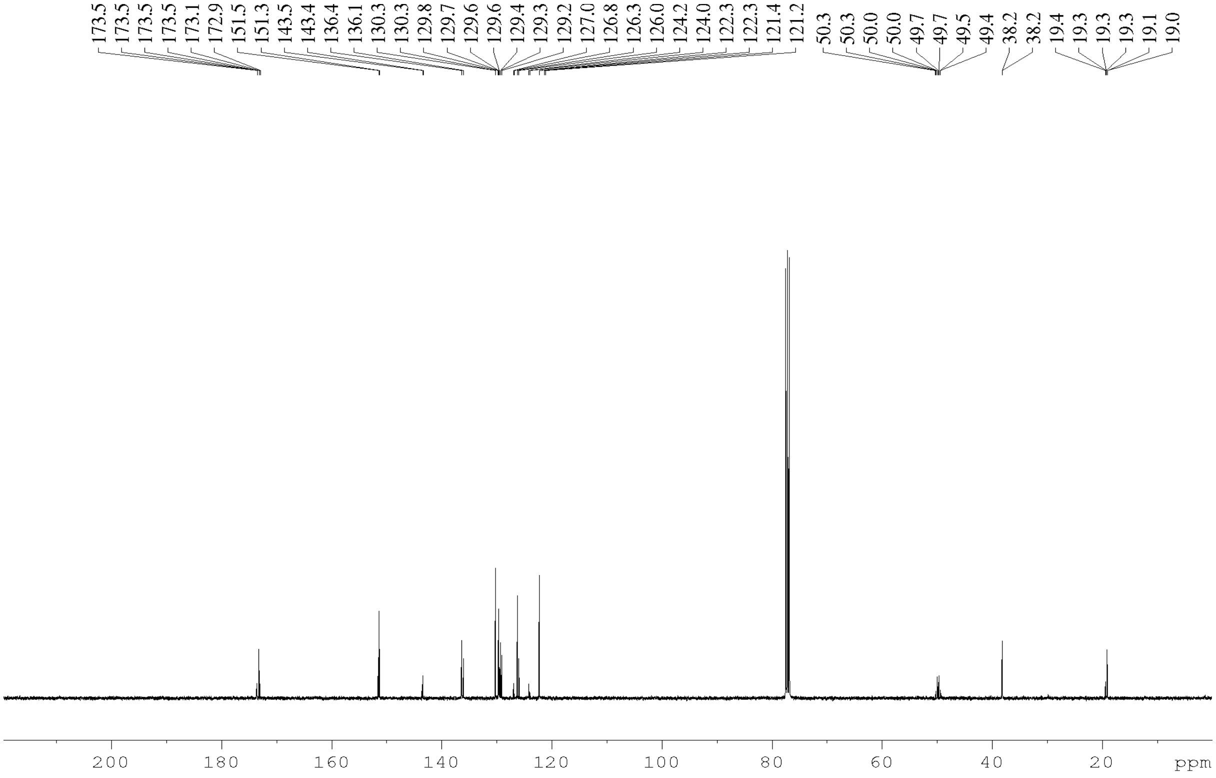

^{19}F NMR ($CDCl_3$, 376 MHz)

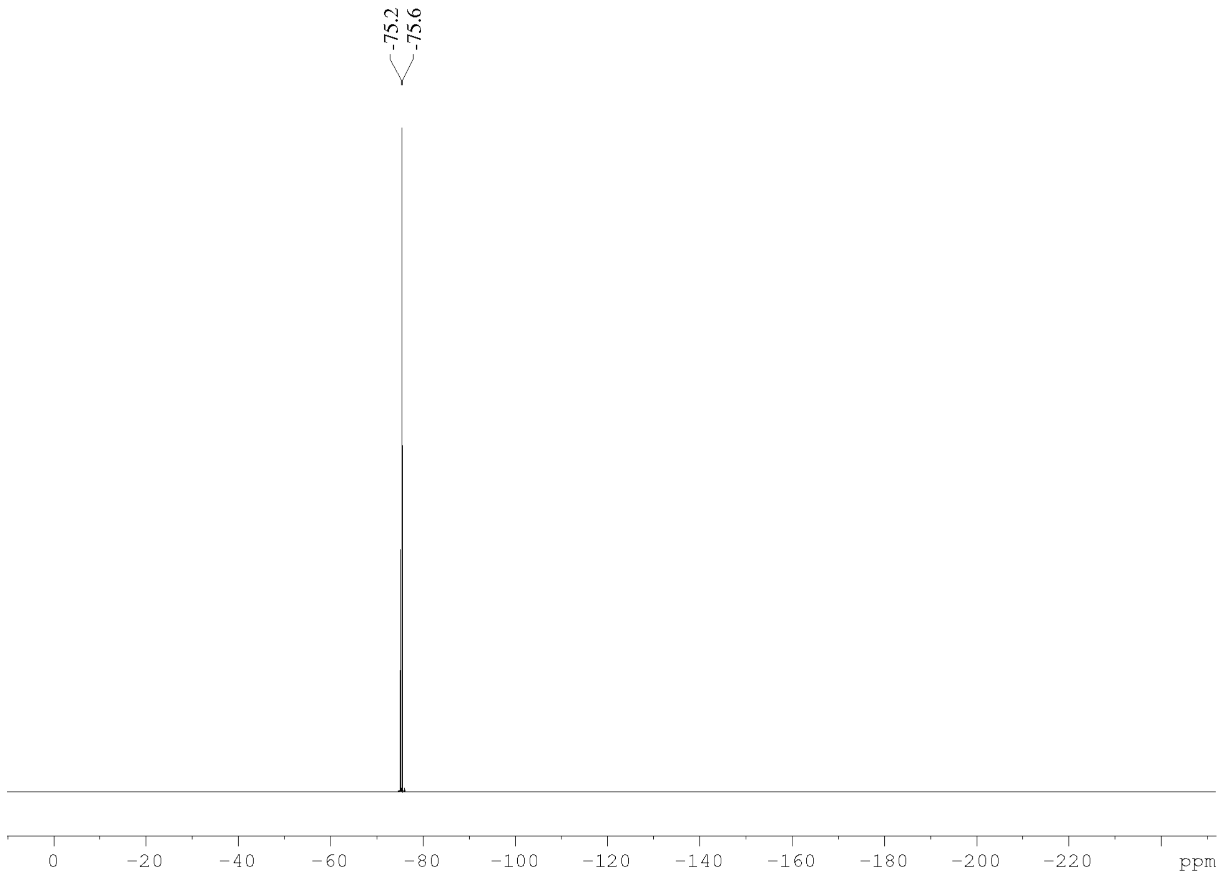

2-(3-methyl-2,5-dioxo-1-(quinolin-8-yl)pyrrolidin-3-yl)isoindoline-1,3-dione (7j)

^{1}H NMR ($CDCl_3$, 400 MHz)

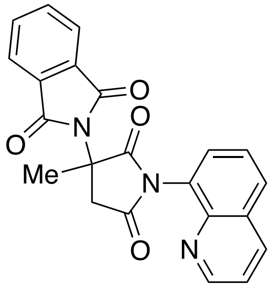

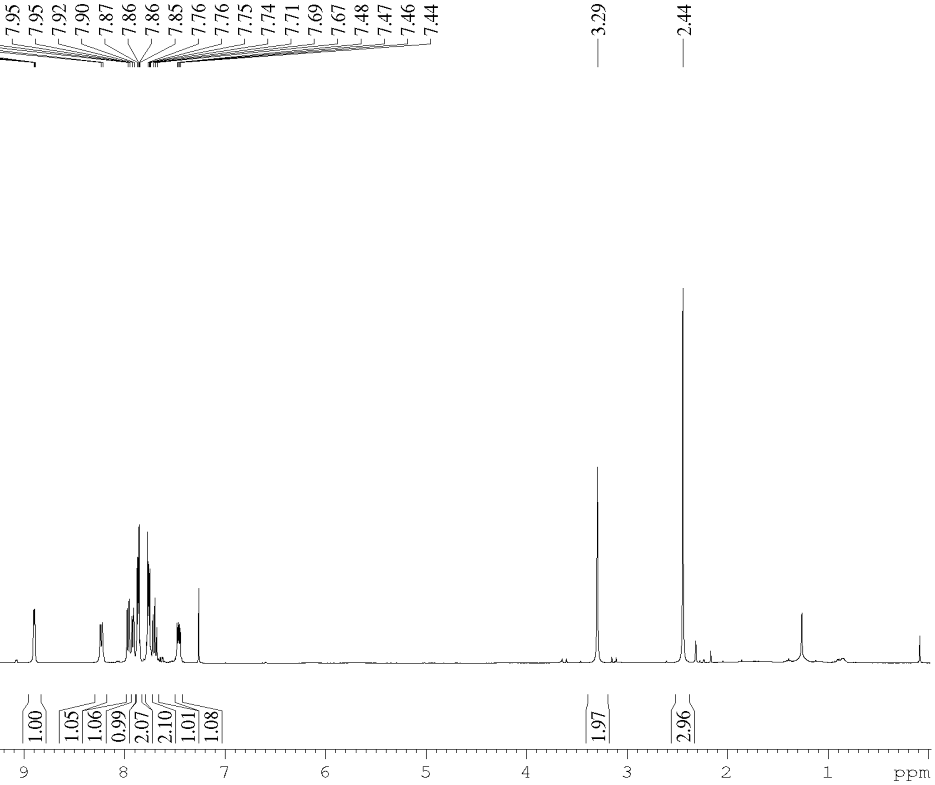

^{13}C NMR ($CDCl_3$, 100 MHz)

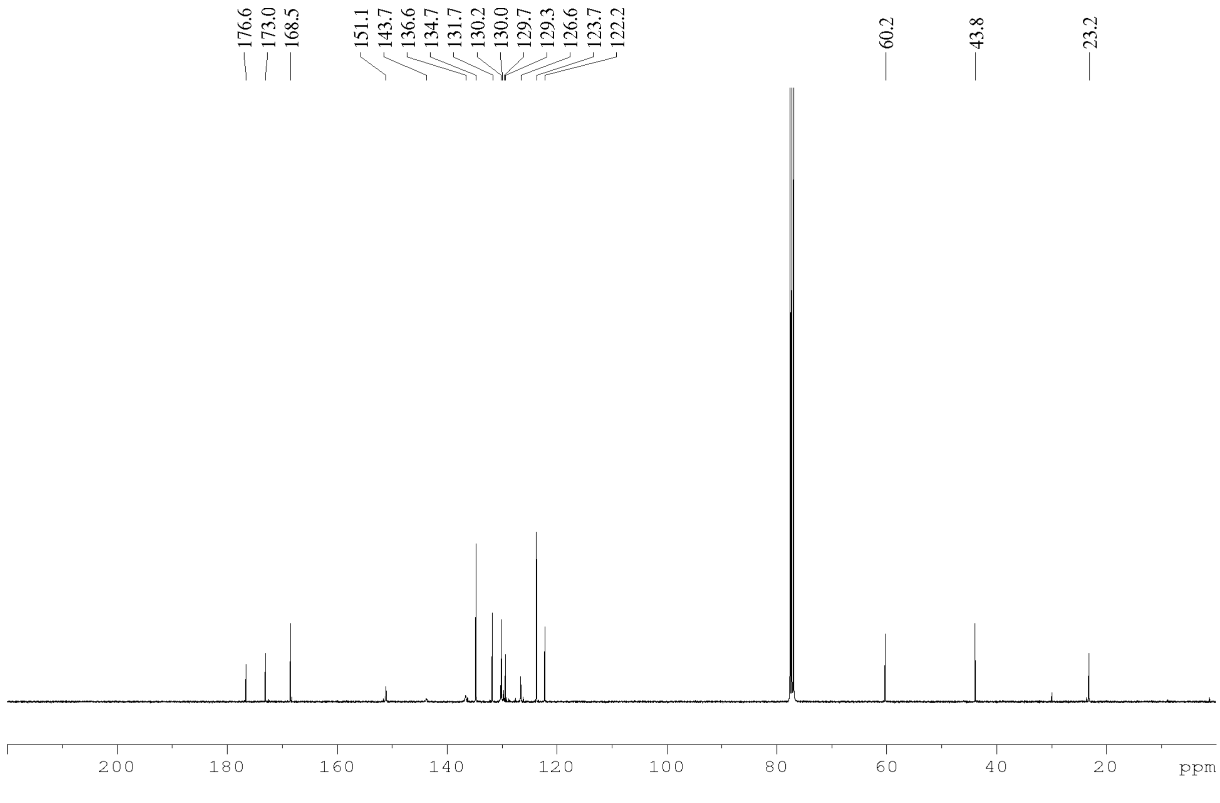

3-(quinolin-8-yl)-3-azabicyclo[3.1.0]hexane-2,4-dione (7k)

^{1}H NMR ($CDCl_3$, 400 MHz)

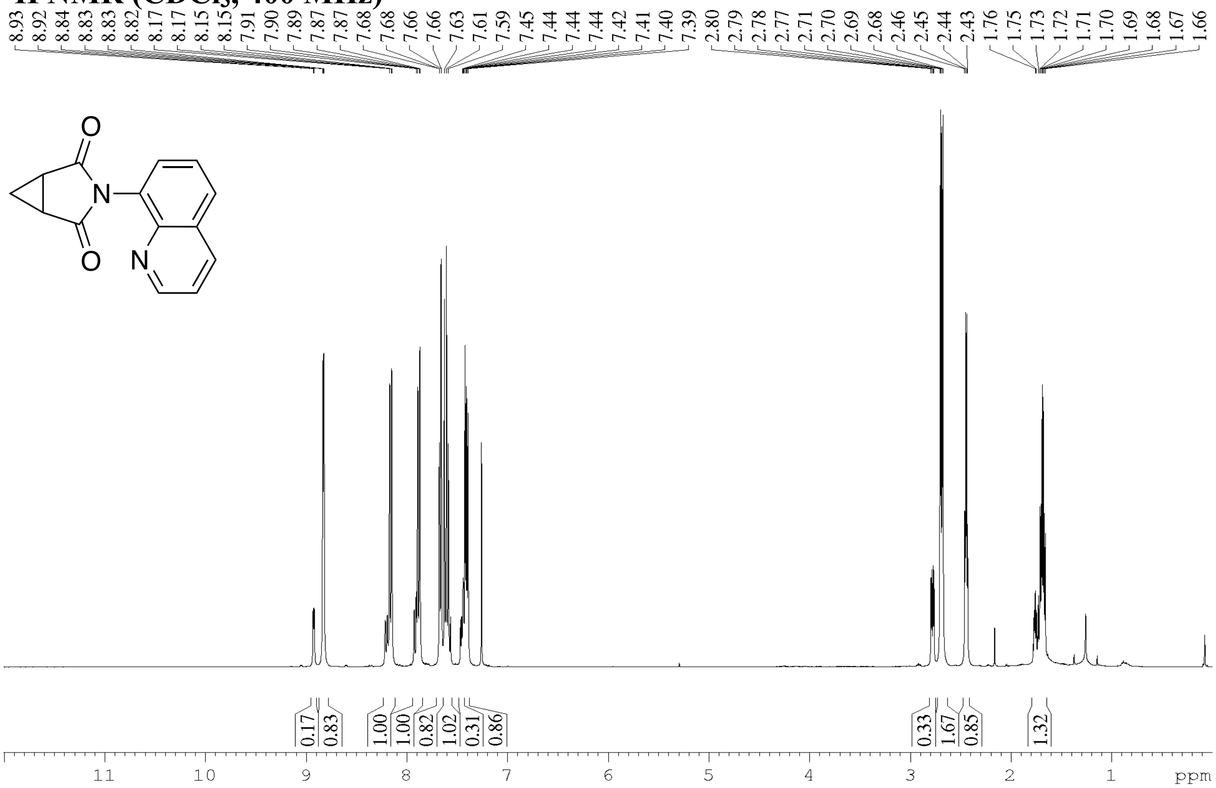

^{13}C NMR ($CDCl_3$, 100 MHz)

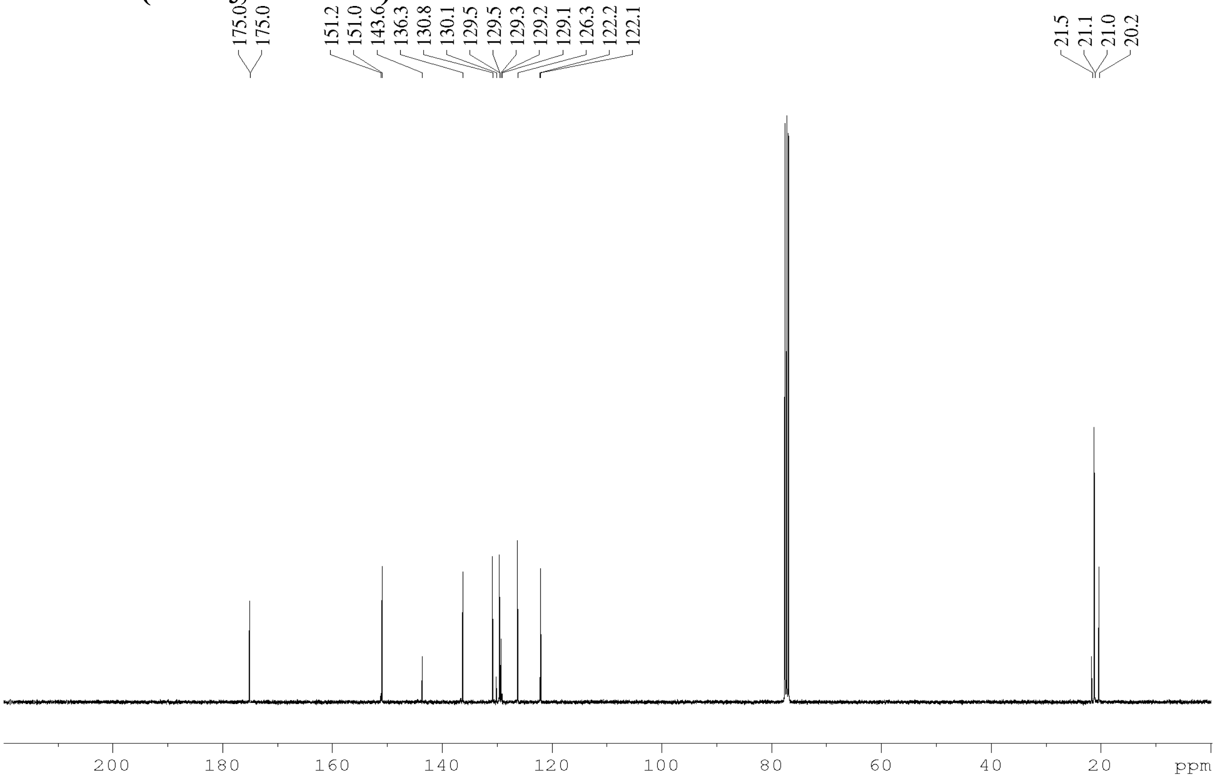

1-methyl-3-(quinolin-8-yl)-3-azabicyclo[3.1.0]hexane-2,4-dione (7l)

^{1}H NMR (CDCl$_3$, 400 MHz)

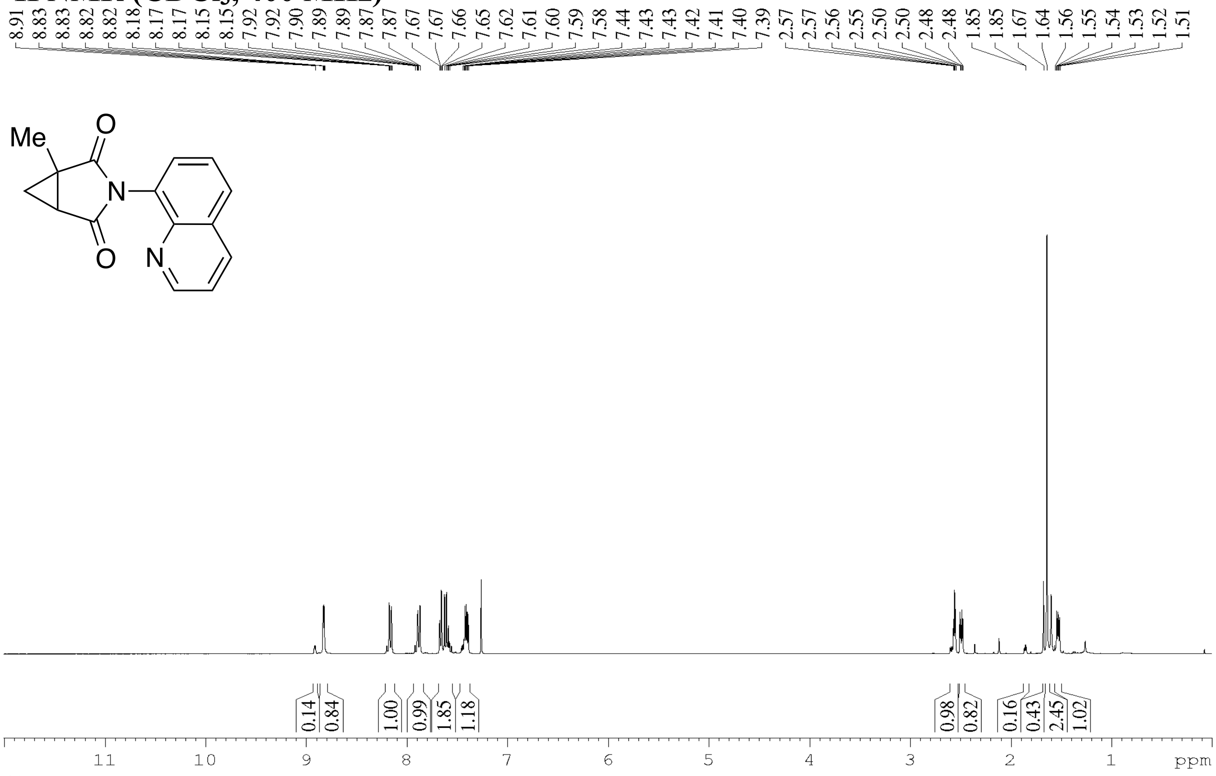

^{13}C NMR (CDCl$_3$, 100 MHz)

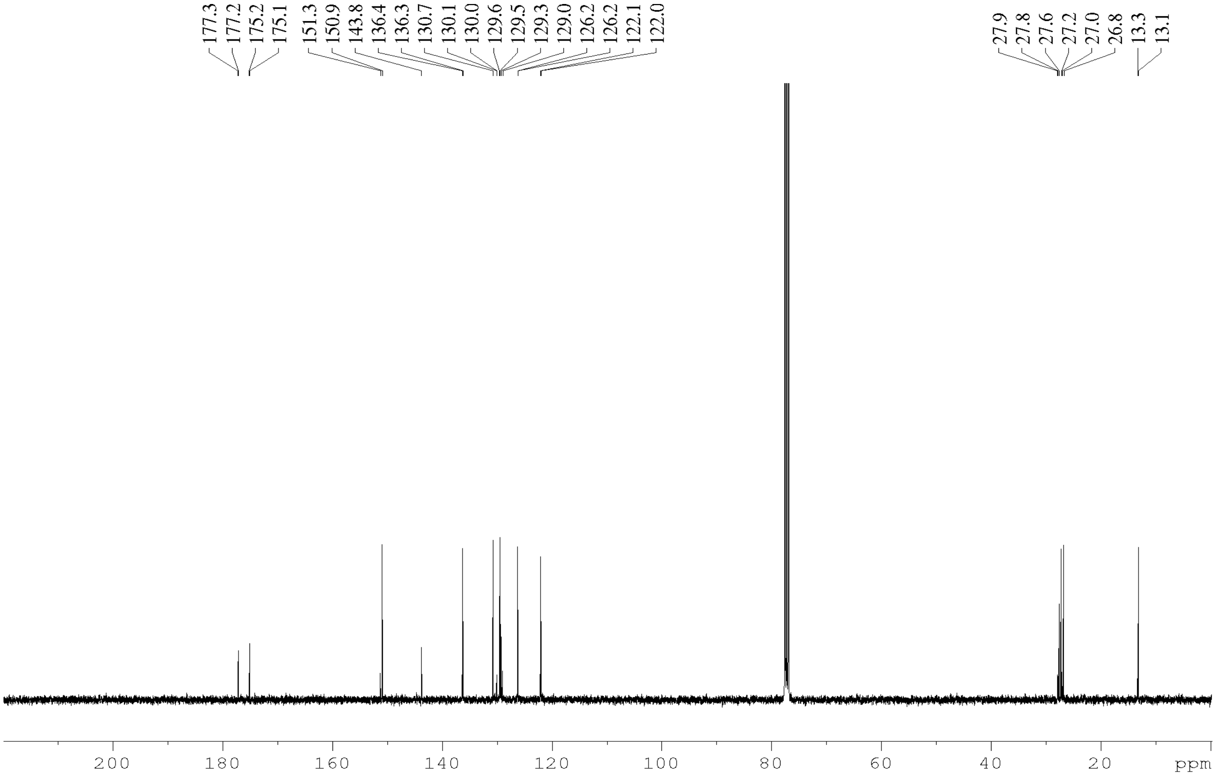

2-(2,4-dioxo-3-(quinolin-8-yl)-3-azabicyclo[3.1.0]hexan-1-yl)isoindoline-1,3-dione (7m)

^{1}H NMR ($CDCl_3$, 400 MHz)

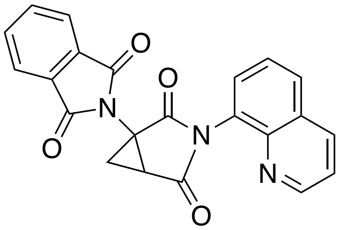

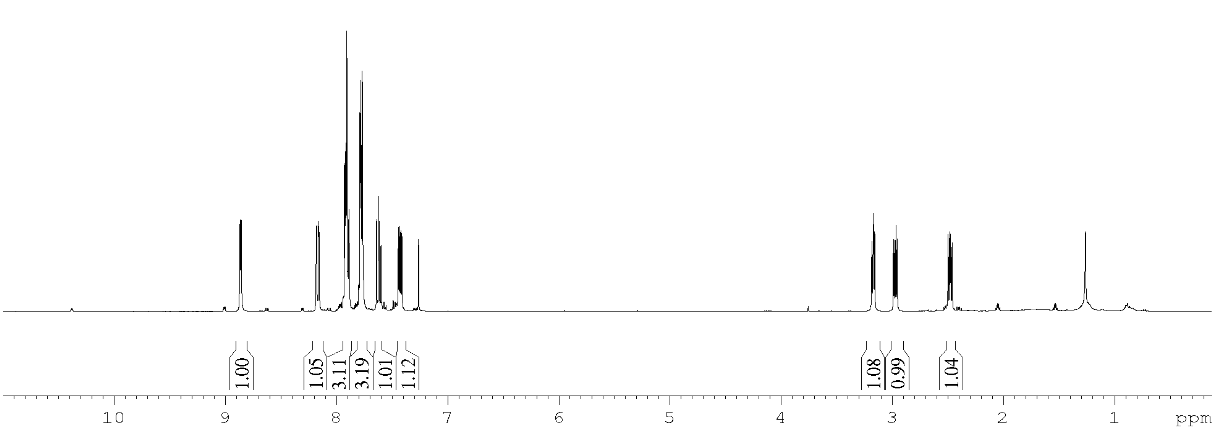

^{13}C NMR ($CDCl_3$, 100 MHz)

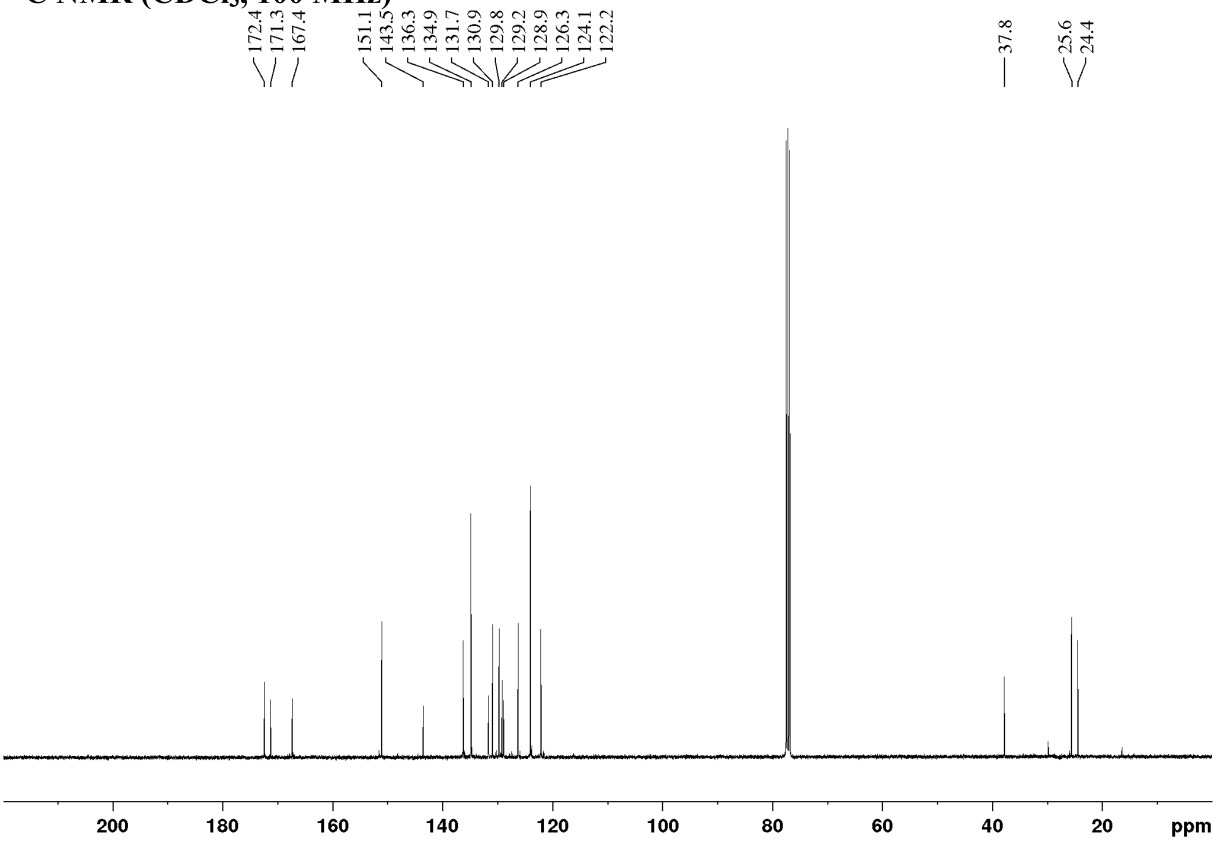

6,6-diphenyl-3-(quinolin-8-yl)-3-azabicyclo[3.1.0]hexane-2,4-dione (7n)

^{1}H NMR ($CDCl_3$, 400 MHz)

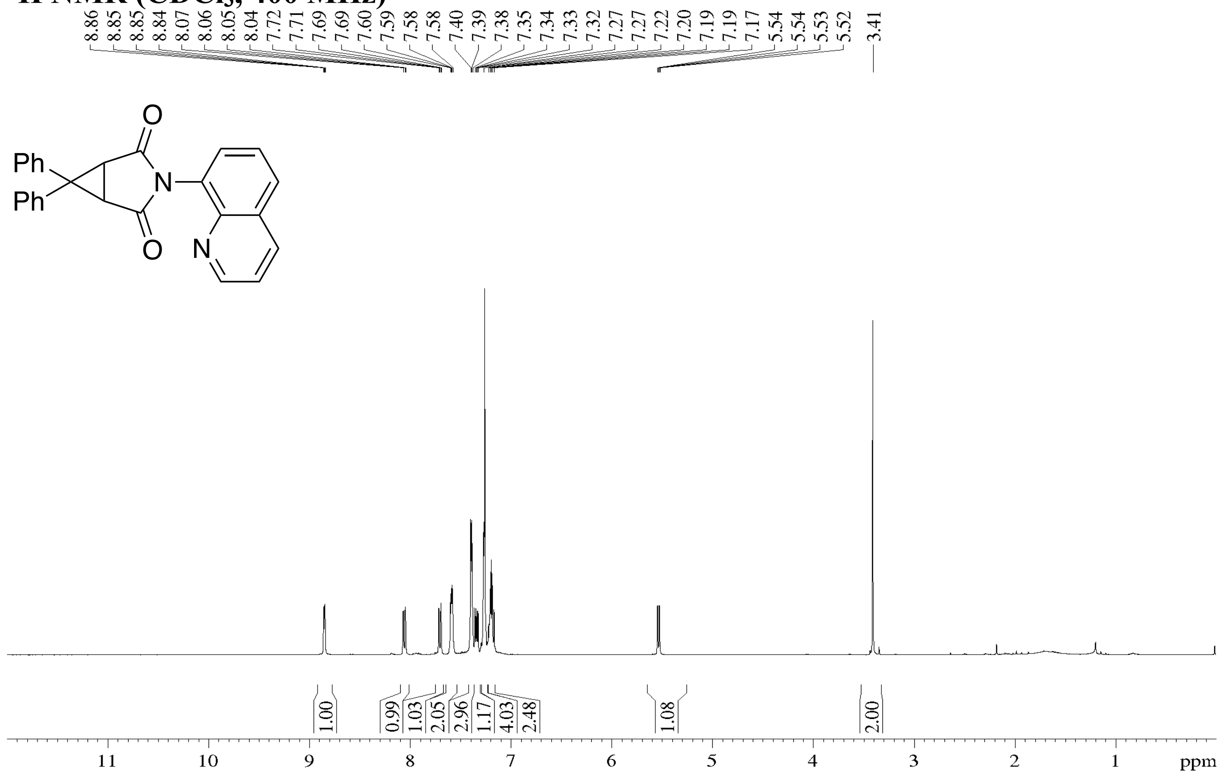

^{13}C NMR ($CDCl_3$, 100 MHz)

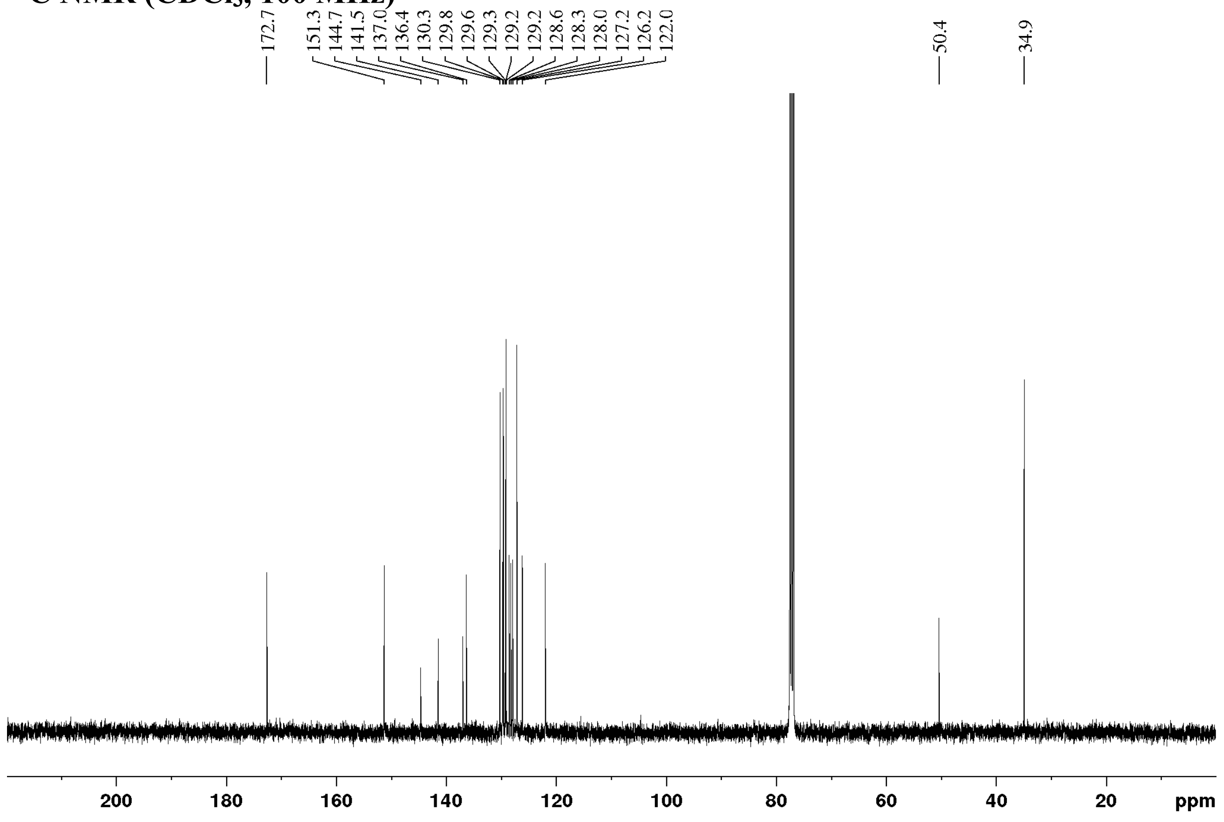

3-methyl-1-(quinolin-8-yl)pyrrolidine-2,5-dione (7o).

^{1}H NMR ($CDCl_3$, 400 MHz)

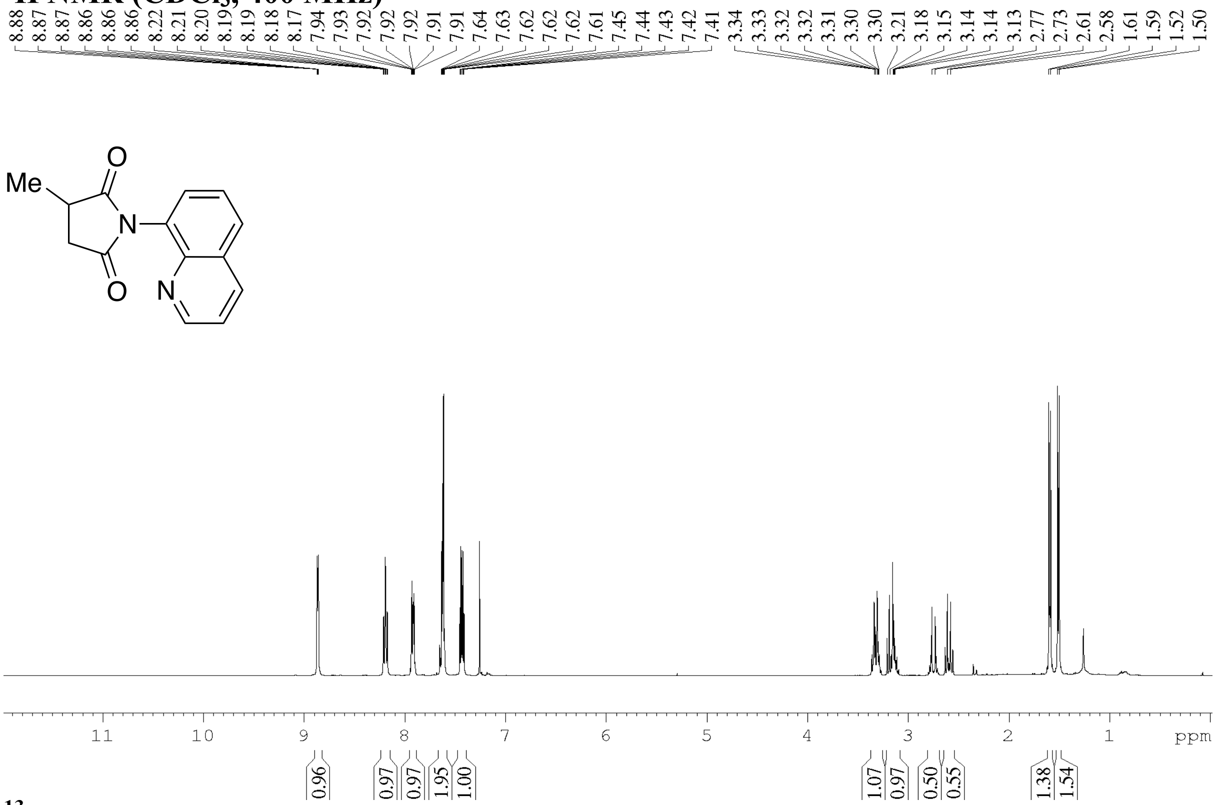

^{13}C NMR ($CDCl_3$, 100 MHz)

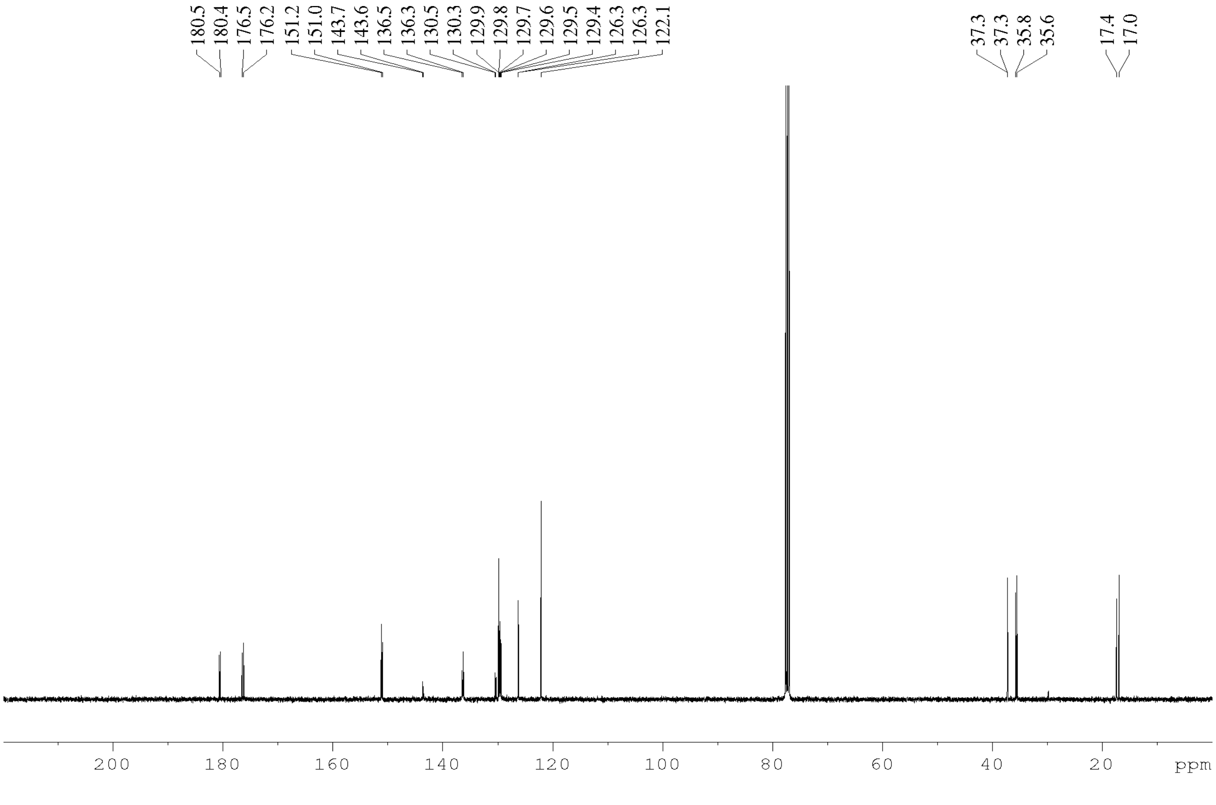

2-(2,5-dioxo-1-(quinolin-8-yl)pyrrolidin-3-yl)isoindoline-1,3-dione (7p)

^{1}H NMR ($CDCl_3$, 400 MHz)

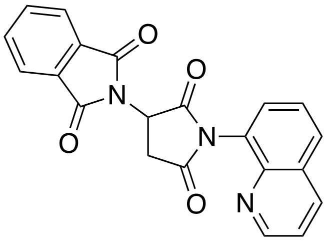

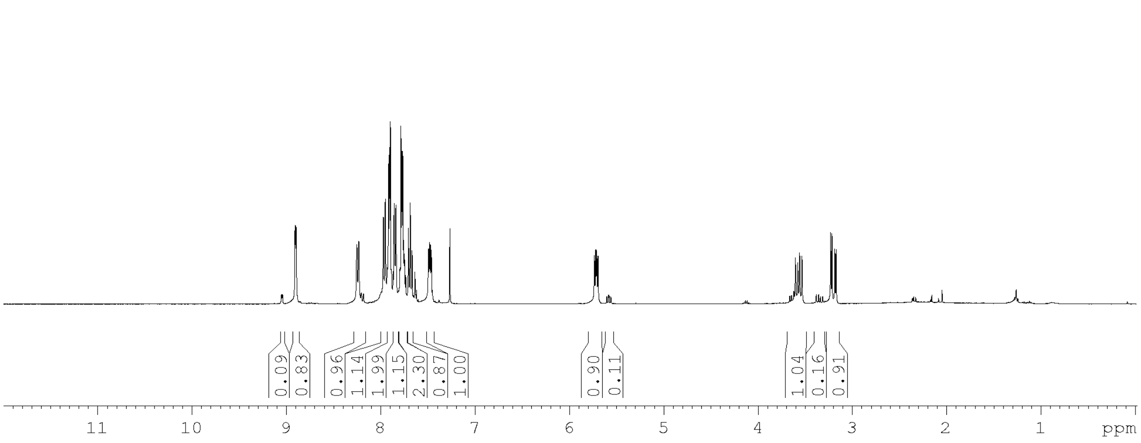

^{13}C NMR ($CDCl_3$, 100 MHz)

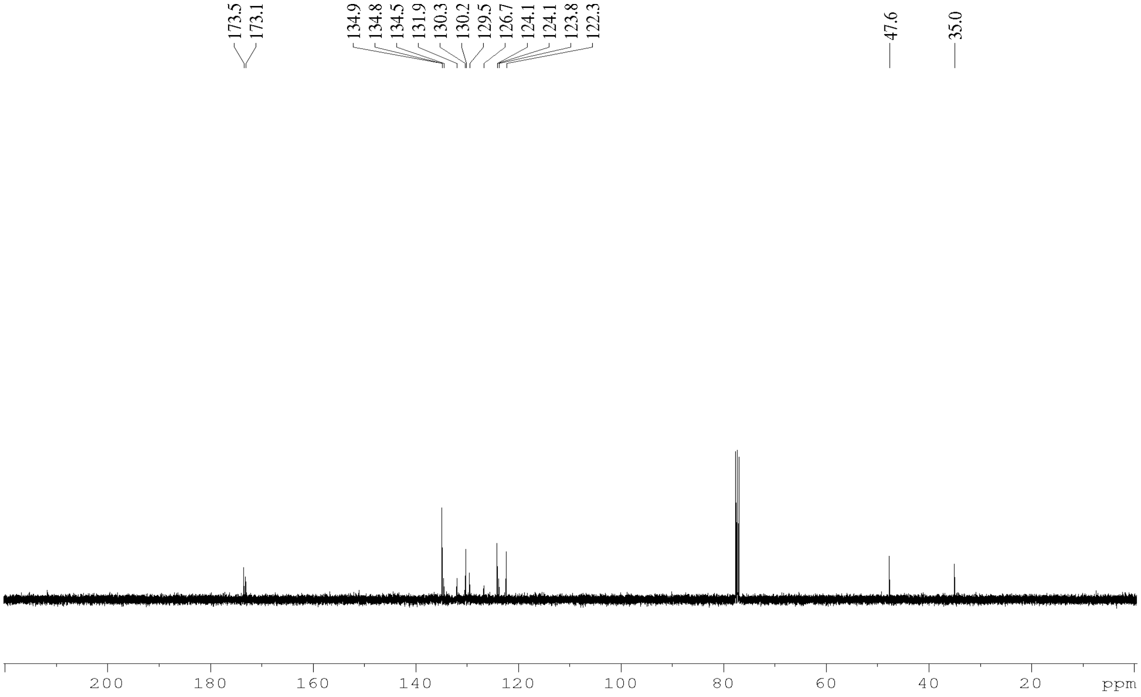

2-methyl-2-phenylsuccinic acid (8)

^{1}H NMR (MeOD, 400 MHz)

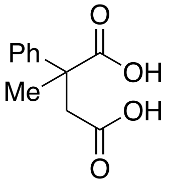

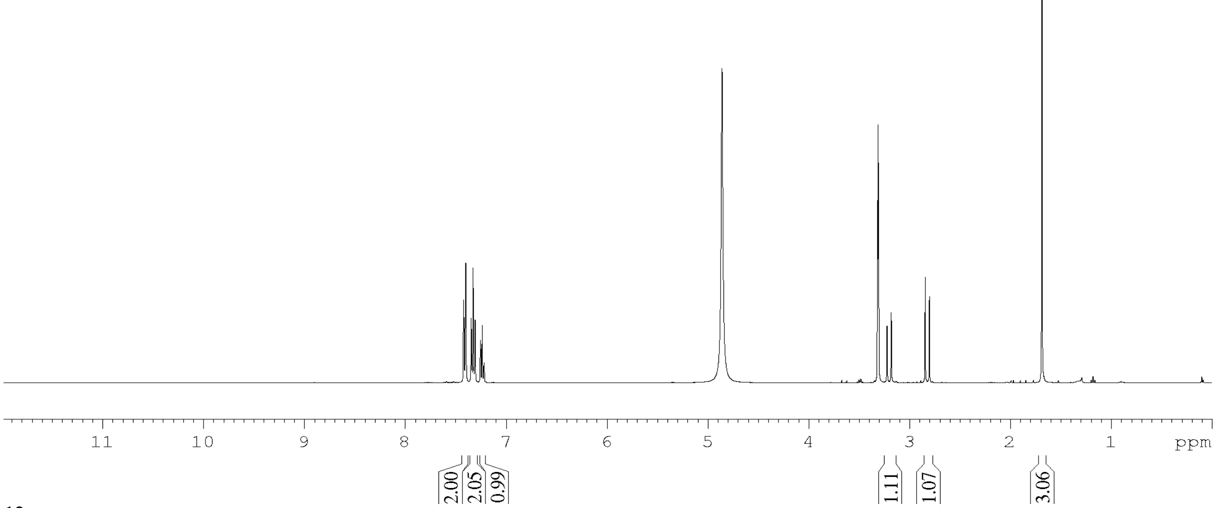

^{13}C NMR (MeOD, 100 MHz)

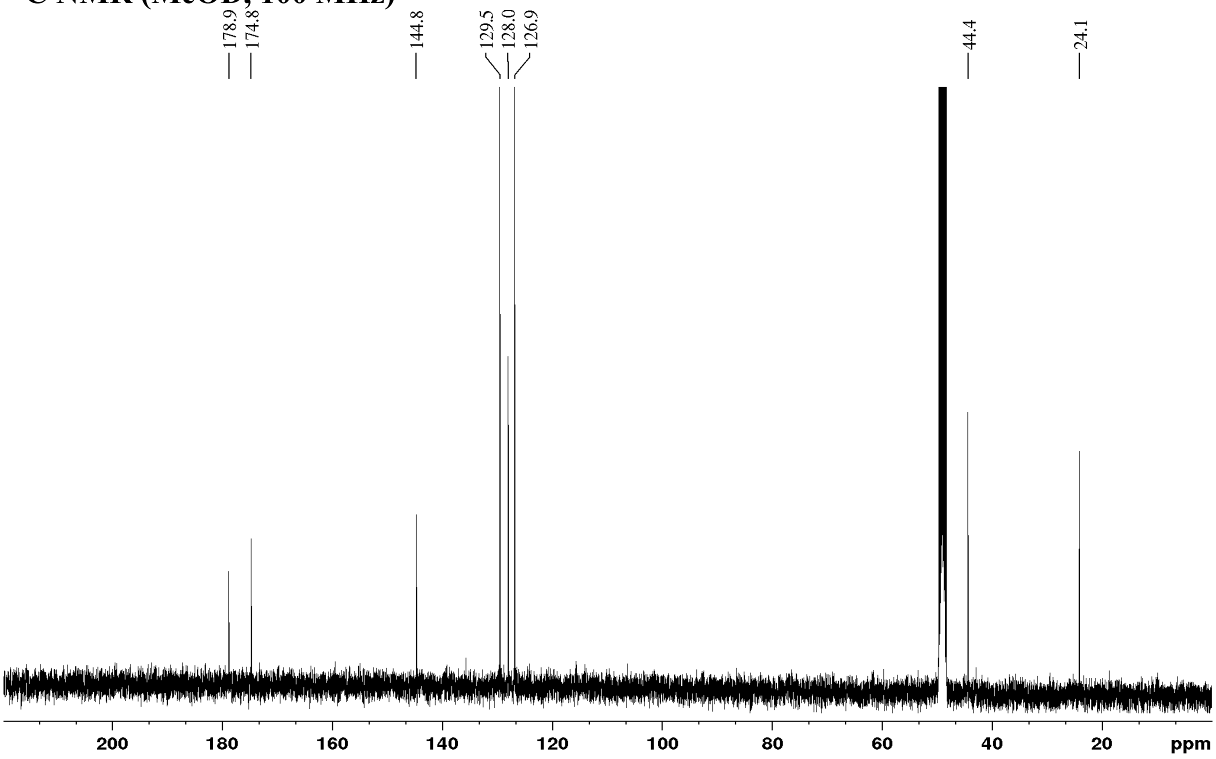

2-methyl-4-morpholino-4-oxo-2-phenyl-N-(quinolin-8-yl)butanamide (9)

^{1}H NMR ($CDCl_3$, 400 MHz)

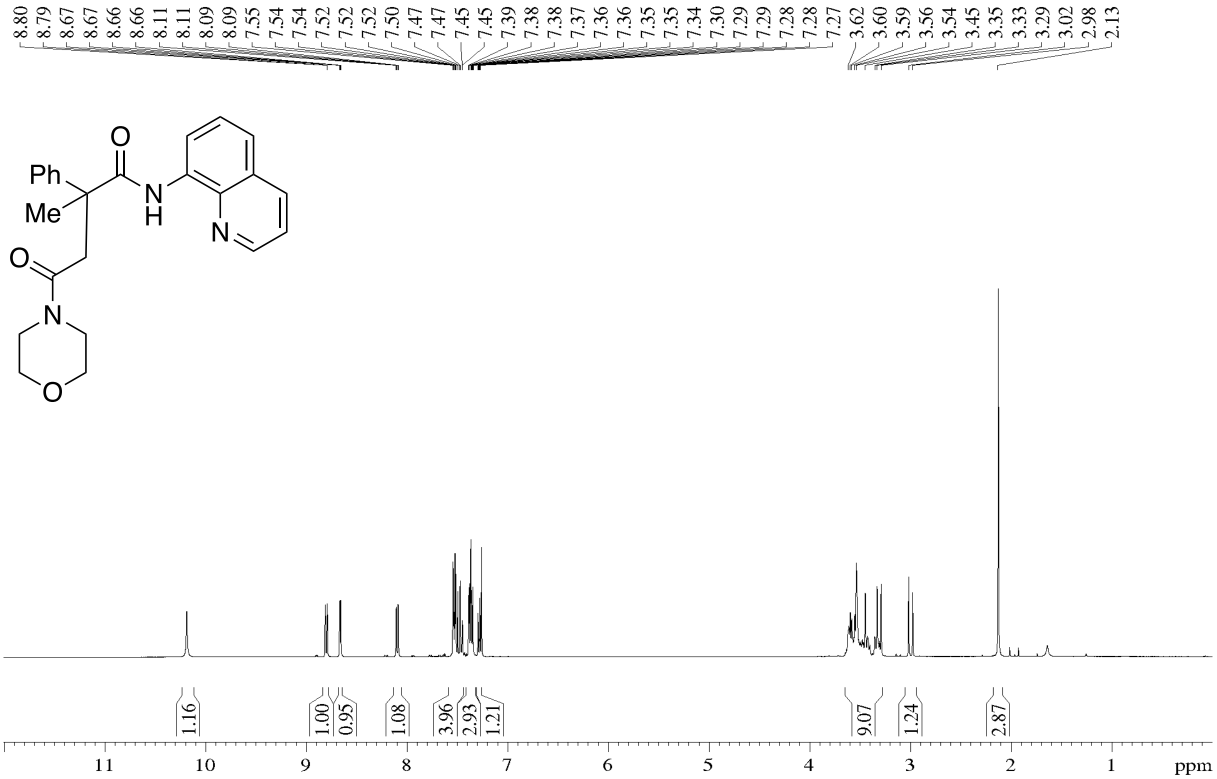

^{13}C NMR ($CDCl_3$, 100 MHz)

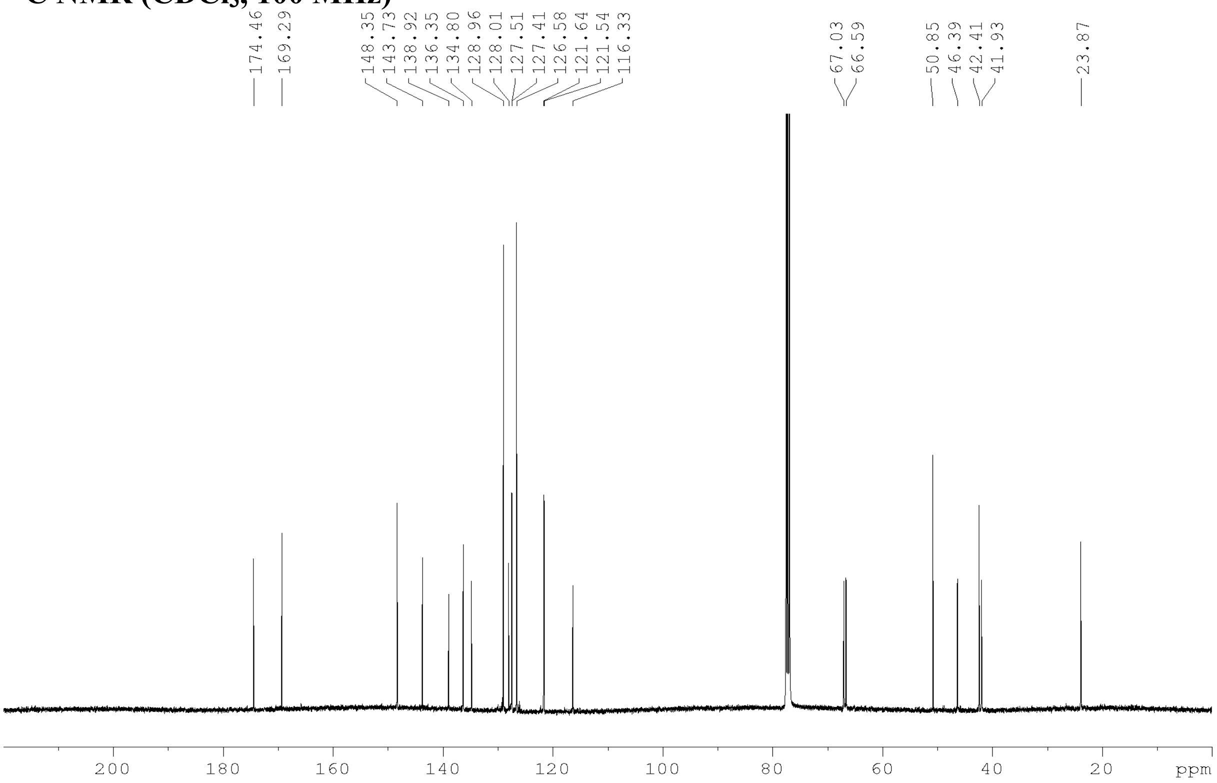

2-methyl-2-phenyl-4-(quinolin-8-ylamino)butan-1-ol (10)

^{1}H NMR ($CDCl_3$, 400 MHz)

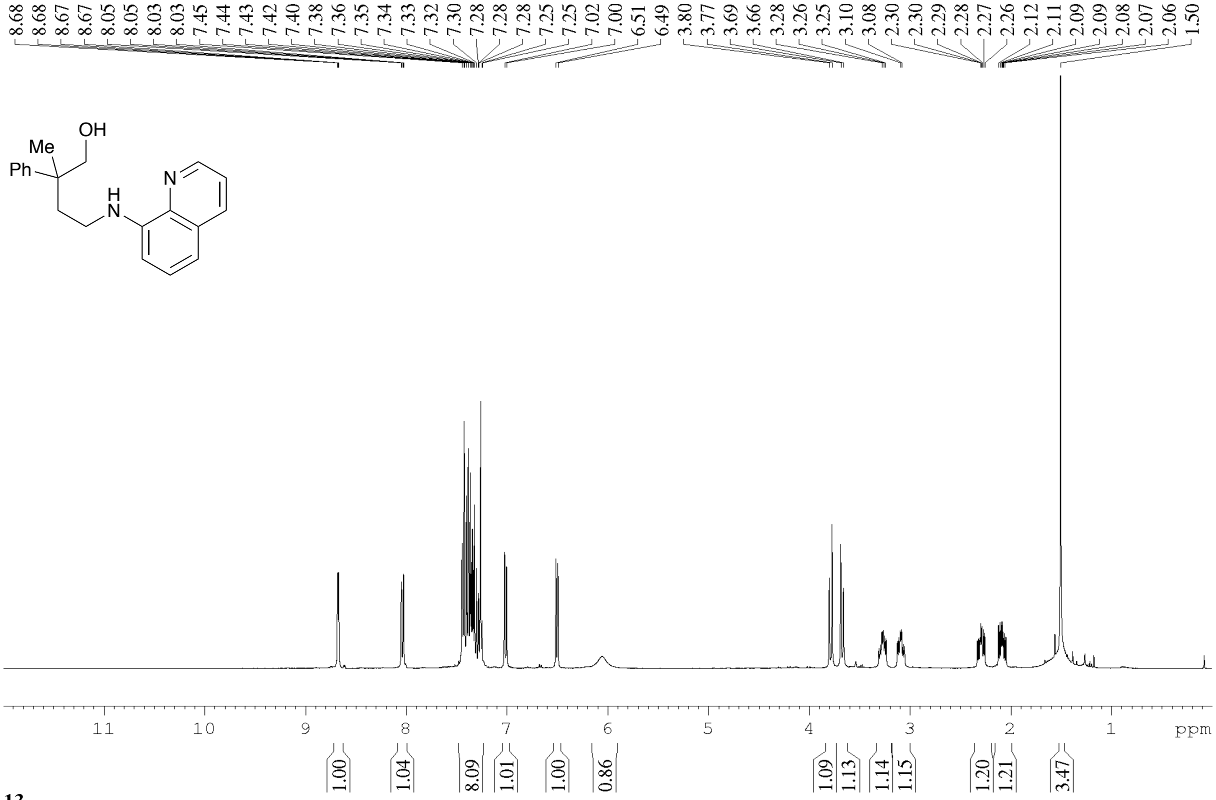

^{13}C NMR ($CDCl_3$, 100 MHz)

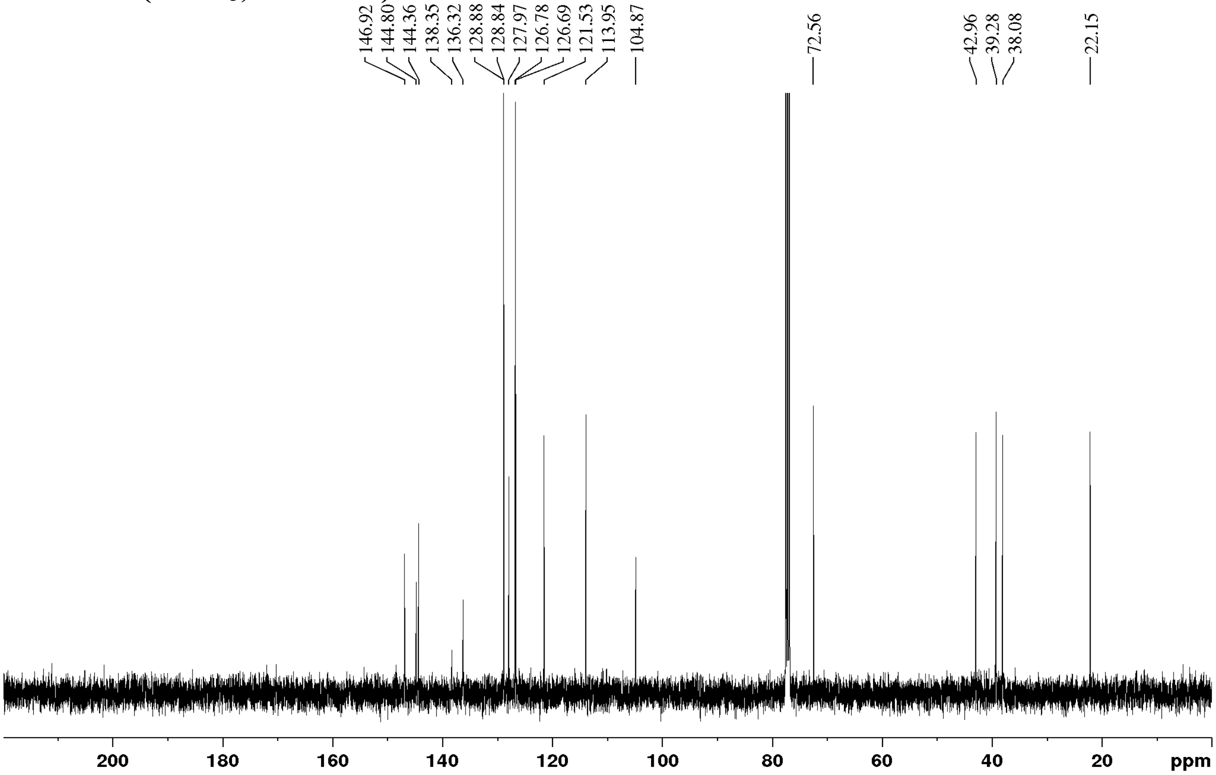

Supplement: Supplementary file 1 [file SC-008-C6SC05581H-s001.pdf]
